# 1,2-Diphenyl-*o*-carborane and Its Chromium Derivatives: Synthesis, Characterization, X-ray Structural Studies, and Biological Evaluations

**DOI:** 10.3390/molecules28134942

**Published:** 2023-06-23

**Authors:** Tae-Jin Ha, Dong-Kyung Im, Seung-Min Kim, Jong-Dae Lee

**Affiliations:** Department of Chemistry, College of Natural Sciences, Chosun University, 309 Pilmundaero, Gwangju 61452, Republic of Korea

**Keywords:** 1,2-diphenyl-*o*-carborane, chromium metal complexes, boron neutron capture therapy, biological evaluation, cytotoxicity

## Abstract

The objective of this study is to design and synthesize substituted η^6^-chromium(0) tricarbonyl metal complexes carrying *o*-carborane units as potential boron neutron capture therapy (BNCT) agents. In this study, 1,2-diphenyl-*o*-carborane (**1**) units were used as starting materials to generate biologically active species. We investigated how the structural changes of **1** substituted with chromium(0) tricarbonyl affect the biological properties, and 1-(Phenyl-η^6^-chromium(0) tricarbonyl)-2-phenyl-*o*-carborane (**2**) and 1,2-bis(phenyl-η^6^-chromium(0) tricarbonyl)-*o*-carborane (**3**) species were produced in moderate yields. The molecular structures of compounds **1**–**3** were identified and established by infrared (IR); ^1^H, ^11^B, and ^13^C nuclear magnetic resonance (NMR) and X-ray crystallography analyses. Crystal structures of 1,2-diphenyl-*o*-carborane and the corresponding chromium complexes **1**, **2**, and **3** were obtained. In an in vitro study using B16 and CT26 cancer cells containing the triphenyl-*o*-carboranyl chromium(0) complexes **Ph3C2BCr2** and **Ph3C2BCr3**, which we reported previously, compounds **2** and **3** accumulated at higher levels than compounds **Ph3C2BCr2** and **Ph3C2BCr3**. However, the phenylated *o*-carboranyl chromium complexes have been found to be more cytotoxic than *p*-boronophenylalanine (BPA).

## 1. Introduction

Boron neutron capture therapy (BNCT) is a promising treatment for a variety of central nervous system (CNS) disorders, especially brain tumors. For example, BNCT is currently used as an adjunct to surgery for the treatment of glioblastoma multiforme [1]. The effect of BNCT is to suppress brain micrometastases that cannot be treated surgically or for which other treatment methods are not available [2]. BNCT destroys cancer cells with energy from targeted radiation bursts by reacting boron isotopes delivered to malignant cells with neutrons. Upon uptake into the cells, ^10^B is externally irradiated with neutrons and becomes unstable [^11^B]*, decaying to lithium (^7^Li^3+^) and releasing high-energy particles (^4^He^2+^). Another advantage of boron is that many ^10^B atoms form a polyhedral cluster, such as B_10_H_10_^2−^ and B_12_H_12_^2−^, enabling a high concentration of boron to be present in the molecule so that a satisfactory therapeutic effect can be expected. The high selectivity of neutron capture by the boron atoms and the effectiveness of treatment using it makes it an acceptable alternative to other chemotherapy methods that destroy cancer cells via high toxicity levels. 

Compound *o*-carborane, namely 1,2-dicarba-*closo*-dodecaborane, C_2_B_10_H_12_, is a boron-rich compound with an icosahedral structure and a diameter similar to that of a rotating benzene ring, nearly 1 nm, with high symmetry and remarkable stability under various conditions [3]. This cluster contains ten boron and two carbon atoms, making it well-suited as a BNCT agent [4,5], and also has potential in other fields of drug discovery, molecular imaging, and targeted radionuclide therapy [6]. However, despite these advantages, the high toxicity and low water solubility of boron agents remains a significant problem impairing further clinical treatment [7].

**Figure 1:**
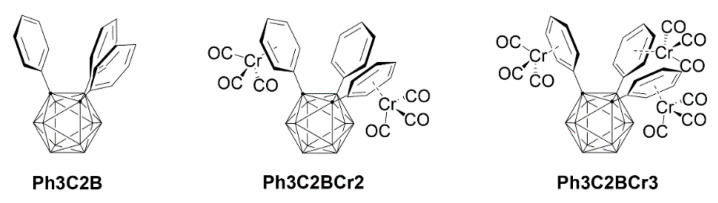
Molecular structure of 1,2,3-triphenyl-*o*-carborane and its chromium(0) tricarbonyl complexes **Ph3C2B**, **Ph3C2BCr2**, and **Ph3C2BCr3**.

Recently, as shown in Figure 1, we reported the successful synthesis and structural characterization of 1,2,3-triphenyl-*o*-carborane and the corresponding chromium complexes (**Ph3C2B**, **Ph3C2BCr2**, and **Ph3C2BCr3**) [8,9]. Among the transition-metal π-complexes, η^6^-arene chromium(0) tricarbonyl complexes have been the subject of extensive development due to their significant applications in organic synthesis [10,11]. Moreover, the use of metal carbonyls in bioorganometallic chemistry is being increasingly developed to enhance the potential of chromium complexes [12,13,14,15].

To the best of our knowledge, this is the first example of the application of η^6^-arene chromium(0) tricarbonyl-substituted *o*-carborane compounds in BNCT. Until now, few η^6^-arene chromium(0) tricarbonyl-substituted *o*-carborane complexes [16,17,18] have been structurally characterized. Moreover, many studies have reported the introduction of various aryl groups to carbons and/or borons in *o*-carborane. However, no biological characteristics have been reported yet for them. This study is significant because it demonstrates the potential of phenylated *o*-carboranes with η^6^-chromium(0) tricarbonyl groups as potential BNCT agents.

## 2. Results and Discussion

### 2.1. Synthesis of 1,2-Diphenyl-o-carborane and Corresponding Chromium Metal Complexes

**Scheme 1:**
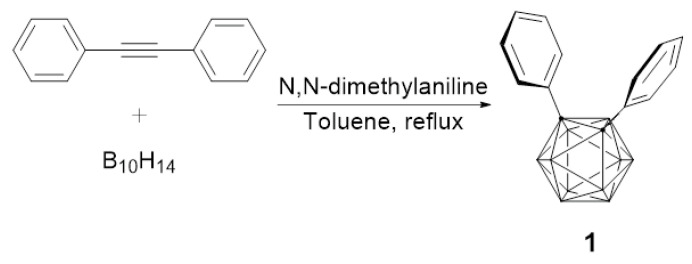
Preparation of 1, 2-diphenyl-*o*-carborane (**1**).

We previously reported the detailed synthesis of the compounds **Ph3C2B**, **Ph3C2BCr2**, and **Ph3C2BCr3** [8,9]. Compound **1** was synthesized by a procedure modified according to previous results [19,20], as shown in Scheme 1. Compound 1,2-Diphenyl-*o*-carborane (**1**) was prepared from decaborane (B_10_H_14_) and diphenylacetylene in moderate yield in toluene solvent using *N*,*N*-dimethylaniline as a base. As a result, we first synthesized icosahedral *o*-carborane with phenyl substituents on the two carbons (Scheme 1).

**Scheme 2:**
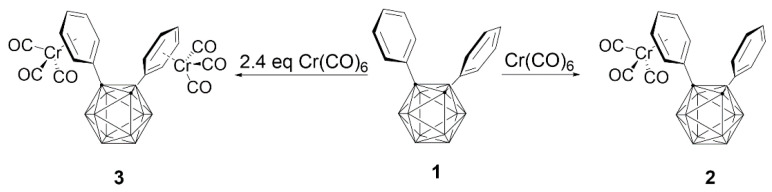
Preparation of mono- and bis-chromium complexes (**2** and **3**).

For comparison with previous results, we tried to confirm the electron-withdrawing ability of *o*-carborane by reacting chromium(0) hexacarbonyl [Cr(CO)_6_] with two phenyl groups introduced into *o*-carborane [21]. The reaction of **1** with Cr(CO)_6_ produced η^6^-phenyl-coordinated mono- and bis-chromium complexes (**2** and **3**) in moderate yields through stoichiometric reactions, as shown in Scheme 2. Interestingly, these results showed that 1,2-diphenyl-*o*-carborane (**1**) prefers stoichiometric reactions to 1,2,3-triphenyl-*o*-carborane.

### 2.2. IR and NMR Spectroscopy

The structure of 1,2-diphenyl-*o*-carborane (**1**) was proposed based on the assignments of the ^1^H, ^11^B, and ^13^C NMR resonances before confirmation by X-ray diffraction (XRD). The ^1^H NMR spectrum of compound **1** showed resonances at around δ 7.45~7.15 for the two phenyl rings. The ^11^B NMR spectrum showed resonances at around δ −11.38~−2.40. The ^13^C NMR spectrum showed resonances at around δ 130.55~85.16. The infrared (IR) spectra of compounds **2** and **3** showed characteristic absorption bands of C≡O units at 1965, 1890 (**2**), 1965, and 1892 cm^−1^ (**3**). These values are lower than those of (C_6_H_6_)Cr(CO)_3_ [22,23,24,25,26]. The IR and NMR spectra clearly indicate Cr coordination with the phenyl groups of **2** and **3**, with chemical shifts to the downfield region of δ 7.715–7.250 and 137.55–130.06 in the ^1^H and ^13^C NMR spectra, respectively (see Figures S1–S9 in Supplementary Materials).

### 2.3. X-ray Structural Studies of 1,2-Diphenyl-o-carborane and Corresponding Chromium Complexes

The selected crystallographic data and a summary of the intensity data collection parameters for **1**, **2**, and **3** are presented in Table 1 and Table 2, respectively. Detailed information on the structural determinations and structural features of compounds **1**, **2**, and **3** are provided in the Supplementary Materials and Appendix A. Additionally, the molecular structures and structural characteristics of **Ph3C2B**, **Ph3C2BCr2**, and **Ph3C2BCr3** are also included (Figures S1–S3 and Tables S10 and S11). Single-crystal X-ray structure determination revealed the structural authenticity of each compound and suggested alterations to the electronic structure based on the changes in the C–C distance of *o*-carborane cage [27]. The C1–C2 bond distance of **1** was 1.726(2) Å, which is significantly longer than the C1–C2 bond distance of the unsubstituted *o*-carborane [1.629(6) and 1.630(6) Å]. Moreover, our comparison of the C–C distances in compounds **1** and **Ph3C2B** (Table S11) showed that the bonds in **Ph3C2B** were slightly longer than those in compound **1**. Compound **Ph3C2B** contained further phenyl decorations at the boron atom while maintaining the active compound **1** platform [28,29].

**Figure 2:**
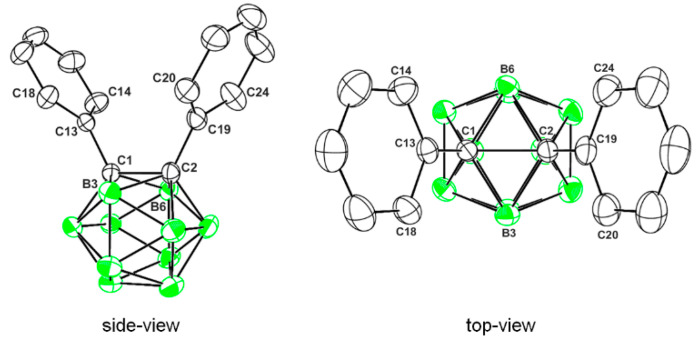
ORTEP drawing (30% probability for thermal ellipsoids) of **1** (the hydrogen atoms are omitted for clarity) (black = carbon, green = boron).

**Figure 3:**
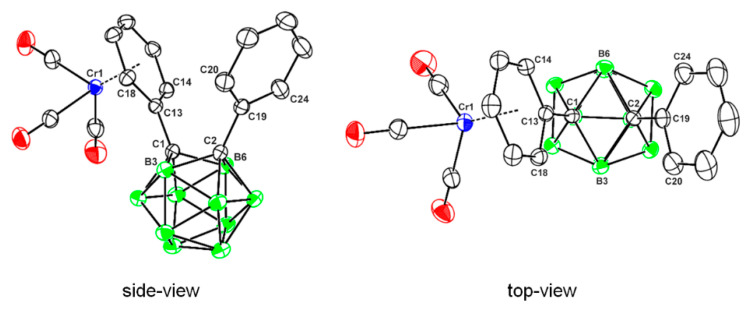
ORTEP drawing (30% probability for thermal ellipsoids) of **2** (the hydrogen atoms are omitted for clarity) (black = carbon, green = boron, blue = chromium, red = oxygen).

**Figure 4:**
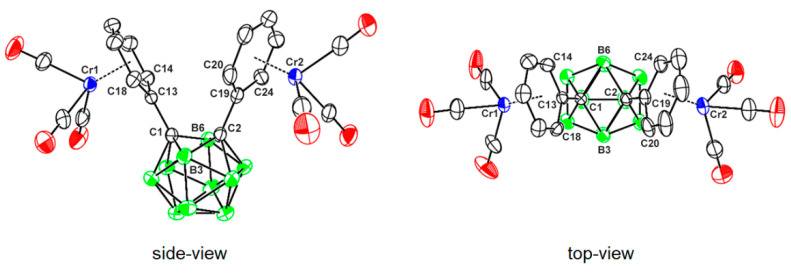
ORTEP drawing (30% probability for thermal ellipsoids) of **3** (the hydrogen atoms are omitted for clarity) (black = carbon, green = boron, blue = chromium, red = oxygen).

Single crystals of **1** and its chromium(0) tricarbonyl complexes, **2** and **3**, suitable for X-ray crystallography, were obtained from dichloromethane solutions by slow evaporation. The general structural characteristics of compound **1** and its chromium complex are shown in Table 2. The molecular structures of compounds **1**, **2**, and **3** are shown in Figure 2, Figure 3 and Figure 4. Compound **1** was reported in 1993 by Lewis and Welch (C_14_H_20_B_10_, *M*w = 296.41, monoclinic, *P*2_1_/*c*, *a* = 10.832(4), *b* = 24.890(13), *c* = 13.9243(21) Å, *β* = 111.881(21)°, *V* = 3483.6 Å^3^, *Z* = 8) [30]. By comparing the data, it can be seen that the bond lengths, angles, and dihedral angles were nearly identical (Figure 2).

As shown in Figure 3, the chromium atom of compound **2** was coordinated to the phenyl ring of the carbon atom of *o*-carborane via a π-bond, similar to the previous results [8,9]. The chromium metal adopted the typical three-legged “piano stool” geometry, with the expected geometrical parameters. The chromium metals were centered approximately over the phenyl rings, giving rise to Cr1–C_6_H_5_ face (centroid) distances of 1.702 Å. The average C–C bond length in the coordinated phenyl ring was 1.410 Å, 0.028 Å longer than the average bond length of 1.382 Å for C–C bonds within the non-coordinated phenyl ring. The Cr–C_Ph_ and Cr–CO bonds had average values of 2.210 and 1.856 Å, respectively, which were within the normal range [31]. The average C–O bond length in the chromium-coordinated carbonyl ligands was 1.143 Å, slightly shorter than that of the reported η^6^-arene chromium(0) tricarbonyl complexes [32,33,34,35,36]. As expected, the C1–C2 bond distance was 1.740(2) Å, slightly greater than compound **1**, and was essentially in the range of non-bonding character [37,38,39,40]. Furthermore, as shown in Table 2 and Table S6, the torsion angles of the two phenyl rings of **1** and **2** changed upon coordination with the chromium atom.

The X-ray crystal structure of **3** (Figure 4) reveals that the dichromium atoms adopted an η^6^-coordination with the two phenyl rings. The single crystal X-ray diffraction study of **3** revealed crystallization in the triclinic space group *P-*1. Interestingly, even though it was a triclinic space group, the APEX3 program identified the Z value as 8, which made it difficult to solve. For this reason, we attempted to obtain better results by changing the crystal system and the space group, but we were unable to obtain satisfactory results. In the end, this was considered to be recognized by four molecules as one molecule in the unit cell, and the structure was interpreted by adjusting the Z value to 2. The geometrical parameters of complex **3** were within the expected ranges (Table 2). The average C–C bond length in the chromium-coordinated phenyl rings was 1.406 Å, 0.03 Å longer than the average bond length of 1.376 Å for the C–C bonds in **1**. The Cr–C_Ph_ and Cr–CO bond lengths were within the normal range [31], with average values of 2.212 and 1.851 Å, respectively. The average C–O bond length in the chromium-coordinated carbonyl ligands was 1.138 Å, significantly shorter than the lengths of reported η^6^-arene chromium(0) tricarbonyl complexes [32,33,34,35,36]. As shown in Table 2, the chromium metals were centered approximately over the phenyl rings, giving rise to Cr1/Cr2–C_6_H_5_ face (centroid) distances of 1.687 and 1.705 Å, respectively. Interestingly, the C1–C2 bond length of **3** was shorter than that of **1** and **2**. It was shown that the contraction of this bond was due to a favorable phenyl ring π*, and carboranyl σ* orbital interactions did not occur in **3**, as has been observed in previous results [8,9]. As shown in Table 2 and Table S9, the torsion angles of the phenyl rings of **1** and **3** or **2** and **3** changed upon coordination with the chromium atoms.

### 2.4. Determination of IC_50_ and Incorporation of Boron into B16 and CT26 Cells

B16 mouse melanoma and CT26 colon carcinoma cells were treated with compounds **2**, **3**, **Ph3C2BCr2**, and **Ph3C2BCr3** for 72 h, after which the cell viability was determined using the MTT [3-(4,5-dimethylthiazol-2-yl)-2,5-diphenyltetrazolium bromide] assay. Compounds **2**, **3**, **Ph3C2BCr2**, and **Ph3C2BCr3** showed higher cytotoxicity than BPA (Table 3), with IC_50_ (the half maximal inhibitory concentration) values in the range of 0.091–0.736 μM. Interestingly, the 1,2,3-triphenyl-*o*-carboranyl chromium(0) tricarbonyl complexes (**Ph3C2BCr2** and **Ph3C2BCr3**) showed higher cytotoxicity to the B16 and CT26 cells than complexes **2** and **3**. The higher cytotoxicity of compounds **Ph3C2BCr2** and **Ph3C2BCr3** to B16 cells may be a result of the difference in the mechanism by which the phenyl groups and chromium(0) tricarbonyl moieties induce cell toxicity. Compounds **2**, **3**, **Ph3C2BCr2**, and **Ph3C2BCr3** exhibited similar activities in the CT26 and B16 cells, with IC_50_ values in the range of 0.089–0.833 μM.

We next examined the level of intracellular accumulation of compounds **2**, **3**, **Ph3C2BCr2**, and **Ph3C2BCr3** by determining their boron concentrations using ICP-OES. The intracellular boron uptake of compounds **2**, **3**, **Ph3C2BCr2**, and **Ph3C2BCr3** in B16 and CT26 cells was higher than that of BPA (Table 3). Boron uptake from both bis- and tris-chromium(0) tricarbonyl-substituted compounds, which included the 1,2,3-triphenyl-*o*-carboranyl chromium tricarbonyl complexes (i.e., **Ph3C2BCr2** and **Ph3C2BCr3**), was lower. These results show that the introduction of phenyl groups or chromium metals into the carborane backbone increases cytotoxicity rather than increasing boron accumulation in cancer cells.

## 3. Materials and Methods

### 3.1. General Procedure

All manipulations were performed under either a dry nitrogen or argon atmosphere using standard Schlenk techniques. Tetrahydrofuran (THF) was distilled from sodium and benzophenone under a nitrogen atmosphere. Elemental analyses were performed using a Carlo Erba Instruments CHNS-O EA 1108 analyzer. High-resolution tandem mass spectrometry (JMS-HX 110/110A, Jeol Ltd.) data were acquired at the Korean Basic Science Institute. ^1^H, ^11^B, and ^13^C NMR spectra were recorded using a Bruker 600 spectrometer operating at 600.1, 150.9, and 192.6 MHz, respectively. All ^11^B chemical shifts were referenced to BF_3_·O(C_2_H_5_)_2_ (0.0 ppm), with a negative sign indicating an upfield shift. All proton and carbon chemical shifts were measured relative to the internal residual CHCl_3_ in the lock solvent (99.9% CDCl_3_). Decaborane was purchased from Katchem. *N*,*N*-dimethylaniline, 1,2-diphenylacetylene, n-BuLi (2.5 M in hexane), and chromium hexacarbonyl [Cr(CO)_6_] were purchased from Aldrich Chemicals.

### 3.2. Crystal Structure Determination

Crystals of **1**, **2**, and **3** were obtained from toluene or CH_2_Cl_2_, sealed in glass capillaries under argon atmosphere, and mounted on a diffractometer. Preliminary examination and data collection were performed using a Bruker SMART CCD detector system single-crystal X-ray diffractometer equipped with a sealed-tube X-ray source (40 kV × 50 mA), using graphite-monochromated Mo-Kα radiation (λ = 0.71073 Å). The preliminary unit cell constants were determined using a set of forty-five narrow-frame (0.3° in ω) scans. Double-pass scanning was used to exclude noise. The collected frames were integrated using an orientation matrix determined from the narrow-frame scans. The SMART software package was used for data collection and SAINT was used for frame integration [41]. The final cell constants were determined by global refinement of the *xyz* centroids of the reflections harvested from the entire dataset. Structure solution and refinement were performed using the SHELXTL-PLUS software package [42].

### 3.3. Cell Viability Assay (MTT Assay)

B16 and CT26 cells were obtained from the Bioevaluation Center of the Korea Research Institute of Bioscience and Biotechnology, Korea. B16 and CT26 cells were cultured in Dulbecco’s modified Eagle’s medium (DMEM; Welgene, Gyeongsan-si, Republic of Korea) containing 10% fetal bovine serum (FBS; Welgene). The cells were cultured at 37 °C in an incubator with 5% CO_2_. The boron compounds were dissolved in dimethylsulfoxide (DMSO), and the resulting solution was diluted with Dulbecco’s modified Eagle’s medium (DMEM) (10% FBS), or *p*-boronophenylalanine (BPA) was directly dissolved in the same medium. In a 96-well culture plate (Falcon 3072), B16 mouse melanoma and CT26 colon carcinoma cells (5 × 10^3^ cells/well) were cultured in five wells with a medium containing the boron compounds at various concentrations. This was followed by incubation for 72 h at 37 °C in a CO_2_ incubator. DMSO is non-toxic at concentrations less than 0.5%, and control experiments confirmed the non-toxicity of DMSO at the concentrations used in the present experiments. After incubation, the medium was removed, the cells were washed three times with phosphate-buffered saline [PBS (–)], and the CellTiter 96^®^ Aqueous Non-Radioactive Cell Proliferation Assay (MTT) was used to count the cells on a microplate reader. The concentration that resulted in a cell culture with 50% of the number of cells in the corresponding untreated group (IC_50_) is summarized in Table 3.

### 3.4. In Vitro Boron Incorporation into B16 and CT26 Cancer Cells

B16 and CT26 cancer cells were cultured in Falcon 3025 dishes (150 mm in diameter). When the cell population increased to fill the dish (5 × 10^5^ cells/dish), the boron compounds and BPA (10 μM) were added to the dishes. The cells were then incubated for 3 h at 37 °C in DMEM (20 mL of 10% FBS). The cells were washed three times with Ca/Mg-free PBS (–), collected with a rubber policeman, digested with a mixture of 60% HClO_4_–30% H_2_O_2_ (1:2) solution (2 mL), and finally, decomposed for 1 h at 75 °C. After filtration through a membrane filter (Millipore, 0.22 mm), the boron concentration was determined using an inductively coupled plasma optical emission spectroscopy (ICP-OES) instrument (Avio 220 Max, PerkinElmer, Waltham, MA, USA). Each experiment was performed in triplicate.

### 3.5. Synthesis of 1,2-Diphenyl-o-carborane (***1***)

Compounds 1,2-Diphenylethyne (2.2 g, 12.0 mmol) and B_10_H_14_ (decaborane) (1.22 g, 10.0 mmol) were dissolved in dry toluene (100 mL) at room temperature under an argon atmosphere. *N,N*-dimethylaniline (2.78 mL, 24.0 mmol) was added to the reaction mixture, and the mixture was stirred at 110 °C for 24 h. After cooling, the solid residue was filtered off and the solvent was evaporated to dryness. The crude mixture was purified using silica gel column chromatography (hexane as the eluent, *R*_f_ = 0.15). Recrystallization from CH_2_Cl_2_ provided **1** as colorless crystals (2.31 g, 78%). HRMS: calculated for [^12^C_14_^1^H_20_^11^B_10_]^+^ 296.2568. Found: 296.2571. IR spectrum (KBr pellet, cm^−1^): ν(C_Ar_–H) 3024, ν(B–H) 2589, ν(C=C_Ar_) 1601, 1500. ^1^H NMR (CDCl_3_, 600 MHz) δ 7.45 (d, 4H, *J* = 7.8 Hz, Ph-*H*), 7.24 (t, 2H, *J* = 7.2 Hz, Ph-*H*), 7.15 (t, 4H, *J* = 7.8 Hz, Ph-*H*). ^11^B{^1^H} NMR (CDCl_3_, 192.6 MHz) δ −2.40, −9.09, −10.37, −11.38. ^13^C NMR (CDCl_3_, 150.9 MHz) δ 130.55, 130.10, 128.21 (*Ph*), 85.16 (*C*_cab_).

### 3.6. Synthesis of 1-(Phenyl-η^6^-chromium(0) tricarbonyl)-2-phenyl-o-carborane (***2***)

Compound **1** (0.3 g, 1.0 mmol) and 1.2 equiv of [Cr(CO)_6_] (0.26 g, 1.2 mmol) were dissolved in a mixture of THF (5 mL) and di-n-butyl ether (50 mL). The mixture was refluxed for 72 h. The resulting dark reddish solution was cooled to room temperature and filtered through Celite. The solvent was evaporated under reduced pressure. After evaporation, the crude reaction mixture was purified by column chromatography [CH_2_Cl_2_:hexane (1:1) as the eluent, *R*_f_ = 0.3~0.35] to give the chromium complex **2**, which was then recrystallized from toluene to obtain red crystals. Yield: 88% (0.38 g, 0.88 mmol). HRMS: calculated for [^12^C_17_^1^H_20_^11^B_10_^52^Cr_1_^16^O_3_]^+^ 432.1821. Found: 432.1824. IR spectrum (KBr pellet, cm^−1^): ν(C_Ar_–H) 3023, 3020, ν(B–H) 2588, ν(C≡O) 1965,1890. ^1^H NMR (CDCl_3_, 600.1 MHz) δ 7.627 (d, 3H, *J* = 7.2 Hz, Ph-*H*), 7.512 (t, 2H, *J* = 7.2 Hz, Ph-*H*), 7.454 (t, 1H, *J* = 7.2 H, Ph-*H*), 7.346 (t, 2H, *J* = 7.2 Hz, Ph-*H*), 7.250 (t, 2H, *J* = 7.8 Hz, Ph-H). ^11^B NMR (CDCl_3_, 192.6 MHz) δ −0.82, −2.88, −4.75, −9.77, −11.05, −14.04. ^13^C NMR (CDCl_3_, 150.9 MHz) δ 221.54 (Cr−*C*O), 137.34, 133.50, 132.19, 131.60, 131.51, 130.16, 130.06 (*Ph*), 87.54 (*C*_cab_).

### 3.7. Synthesis of 1,2-bis(phenyl-η^6^-chromium(0) tricarbonyl)-o-carborane (***3***)

A procedure analogous to the preparation of **2** was used to obtain red crystals. Yield: 57% (0.32 g, 0.57 mmol). HRMS: calculated for [^12^C_20_^1^H_20_^11^B_10_^52^Cr_2_^16^O_6_]^+^ 568.1073. Found: 568.1077. IR spectrum (KBr pellet, cm^−1^): ν(C_Ar_–H) 3021, ν(B–H) 2583, 2589; ν(C≡O) 1965, 1892. ^1^H NMR (CDCl_3_, 600.1 MHz) δ 7.715 (d, 4H, *J* = 7.8 Hz, Ph-*H*), 7.497 (t, 2H, *J* = 7.2 Hz, Ph-*H*), 7.406 (t,, 4H, *J* = 7.8 Hz, Ph-*H*). ^11^B NMR (CDCl_3_, 192.6 MHz) δ −2.50, −9.49, −11.07, −11.83. ^13^C NMR (CDCl_3_, 150.9 MHz) δ 228.85 (Cr-*C*O), 137.55, 136.15, 134.31 (*Ph*), 87.17 (*C*_cab_).

## 4. Conclusions

In conclusion, this study describes the synthesis, X-ray structures, and biological activities of a series of 1,2-diphenyl- and 1,2,3-triphenyl-*o*-carborane compounds and their corresponding chromium(0) tricarbonyl transition metal complexes, which can be easily stoichiometrically substituted with transition metals to produce highly active biological molecules for BNCT. We presented a general and versatile method for the sequential introduction of phenyl groups into *o*-carborane and stoichiometrically-coordinated transition metal complexes using chromium(0) hexacarbonyl. Interestingly, it was confirmed that the C1–C2 bond length of compound **1** was the longest in compound **2** and the shortest in compound **3**. We found that this affected the interaction of the π* orbital of the phenyl group with the σ* orbital of the carborane carbon by functioning as a strong π-receptor to form stable new types of organometallic complexes (**2** and **3**) through metal(Cr)-to-ligand(Ph) back-bonding interactions. The results clearly show the electron withdrawal properties of the *o*-carborane when the two phenyl groups are placed face-to-face with each other. Diphenyl- or triphenyl-*o*-carborane and the corresponding chromium complexes showed higher cytotoxicity than *p*-boronophenylalanine in B16 and CT26 cancer cell lines; however, boron accumulation was higher than that of *p*-boronophenylalanine. As a result, it was confirmed that as more chromium metal atoms and/or phenyl groups were substituted in the *o*-carborane backbone, cytotoxicity increased, but boron accumulation decreased.

## Figures and Tables

**Table 1:** Crystal data and structure refinement of **1–3**.

	1	2	3
Identification code	K120504	K130805	K131105
Empirical formula	C_14_H_20_B_10_	C_17_H_20_B_10_Cr_1_O_3_	C_20_H_20_B_10_Cr_2_O_6_
Formula weight	296.40	432.43	568.46
Temperature	293(2) K	293(2) K	293(2) K
Wavelength	0.71073 Å	0.71073 Å	0.71073 Å
Crystal system, space group	Monoclinic, *P*2_1_/n	Monoclinic, *P*2_1_/n	Triclinic, *P*ī
Unit cell dimensions	*a* = 10.859(1) Å	*a* = 10.621(3) Å	*a* = 17.540(2) Å, *α* = 105.746(2)°
	*b* = 24.953(3) Å, *β* = 111.854(2)°	*b* = 17.056(5) Å, *β* = 106.622(5)°	*b* = 18.060(2) Å, *β* = 110.226(2)°
	*c* = 13.938(2) Å	*c* = 12.174(4) Å	*c* = 19.484(3) Å, *γ* = 91.256(2)°
Volume	3505.3(8)	2113.2(1)	5528.0(1)
*Z*, *D*_calc_	8, 1.123	4, 1.359	2, 0.342
F(000)	1232.0	880.0	572
Crystal size	0.15, 0.13, 0.12	0.17, 0.15, 0.13	0.20, 0.20, 0.15
*θ* range for data collection	1.63 to 28.37	2.12 to 28.46	1.17 to 28.38
Limiting indices	−14 ≤ *h* ≤ 14, −33 ≤ *k* ≤ 32, −18 ≤ *l* ≤ 18	−14 ≤ *h* ≤ 14, −22 ≤ *k* ≤ 22, −16 ≤ *l* ≤ 16	−23 ≤ *h* ≤ 23, −24 ≤ *k* ≤ 24, −26 ≤ *l* ≤ 25
Reflections collected/unique	35826/8726 [R(int) = 0.0421]	28490/5305 [R(int) = 0.0238]	44259/18014 [R(int) = 0.0398]
Completeness to *θ* = 28.38	99.3%	99.2%	65.0%
Refinement method	Full-matrix least-squares on *F*^2^	Full-matrix least-squares on *F*^2^	Full-matrix least-squares on *F*^2^
Data/restraints/parameters	8726/0/581	5305/0/360	18014/0/1409
Goodness-of-fit on *F*^2^	1.008	1.053	0.902
Final *R* indices [*I* > 2*θ*(*I*)]	*^a^ R*_1_ = 0.0616, *^b^ wR*_2_ = 0.1545	*^a^ R*_1_ = 0.0332, *^b^ wR*_2_ = 0.0958	*^a^ R*_1_ = 0.0563, *^b^ wR*_2_ = 0.1505
*R* indices (all data)	*^a^ R*_1_ = 0.1037, *^b^ wR*_2_ = 0.1897	*^a^ R*_1_ = 0.0388, *^b^ wR*_2_ = 0.1018	*^a^ R*_1_ = 0.0877, *^b^ wR*_2_ = 0.1612
Largest diff. peak and hole	0.220 and −0.355 e.Å−3	0.364 and −0.242 e.Å−3	0.687 and −0.375 e.Å−3

*^a^ R*_1_ = ∑||*F*_o_| − |*F*_c_|| (based on reflections with *F*_o_^2^ > 2σ*F*^2^), *^b^ wR*_2_ = [∑[*w*(*F*_o_^2^-*F*_c_^2^)^2^]/∑[*w*(*F*_o_^2^)^2^]]^1/2^; *w* = 1/[σ^2^(*F*_o_^2^) + (0.095*P*)^2^]; *P* = [max(*F*_o_^2^, 0) + 2*F*_c_^2^]/3(also with *F*_o_^2^ > 2σ*F*^2^).

**Table 2:** Comparison of selected bond lengths (Å), angles (°), and torsion angles (°) for **1**–**3**.

	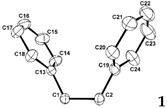	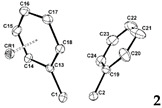	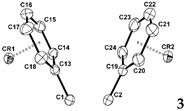
Ph_C−C_ (av)	1.376	1.400	1.406
Ph_C−C_ (av)−Cr		2.210	2.212
Cab_C−C_	1.726(2)	1.740(2)	1.724(4)
Cent−Cr		1.702	1.696(av)
Cr–CO		1.856(av)	1.851(av)
C1–C13	1.507(2)	1.499(2)	1.502(4)
C2–C19	1.501(2)	1.500(2)	1.510(4)
C13–C1–C2	118.3(1)	116.6(1)	116.4(2)
C19–C2–C1	119.0(1)	119.6(1)	116.1(2)
C1-C2-C19-C20	84.1(2)	86.1(2)	105.8(3)
C2-C1-C13-C14	81.7(2)	102.3(1)	112.4(3)

**Table 3:** Cytotoxicity (IC_50_) and boron accumulation of B16 melanoma and CT26 colon carcinoma cells.

Compounds	B16		CT26	
	Cytotoxicity IC_50_ (M) *^a^*	Boron Accumulation (ppm) *^b^*	Cytotoxicity IC_50_ (M) *^a^*	Boron Accumulation (ppm) *^b^*
**2**	0.736 × 10^−6^ (±0.01)	0.825 ± 0.003	0.833 × 10^−6^ (±0.03)	0.755 ± 0.009
**3**	0.681 × 10^−6^ (±0.04)	0.620 ± 0.002	0.314 × 10^−6^ (±0.07)	0.694 ± 0.002
**Ph3C2BCr2**	0.411 × 10^−6^ (±0.06)	0.384 ± 0.006	0.164 × 10^−6^ (±0.05)	0.402 ± 0.002
**Ph3C2BCr3**	0.091 × 10^−6^ (±0.03)	0.221 ± 0.001	0.089 × 10^−6^ (±0.08)	0.247 ± 0.001
BPA	4.871 × 10^−5^ (±0.03)	0.103 ± 0.002	3.862 × 10^−3^ (±0.04)	0.514 ± 0.001

*^a^* B16 melanoma and CT26 colon carcinoma cancer cells (5 × 10^3^ cells) were incubated for 72 h in the presence of compounds **2**, **3**, **Ph3C2BCr2**, and **Ph3C2BCr3**, and then the percentages of viable cells were determined by MTT assay. The drug concentrations required to inhibit cell viability by 50% (IC_50_) were determined from semi-logarithmic concentration–response plots, and the results represent the means ± s.d. of triplicate samples. *^b^* B16 and CT26 cells (5 × 10^5^ cells) were incubated for 3 h in the presence of compounds **2**, **3**, **Ph3C2BCr2**, and **Ph3C2BCr3** or BPA (10.8 ppm). After washing three times, the accumulated boron concentrations were determined by inductively coupled plasma optical emission spectroscopy (ICP-OES). The values shown are the means ± s.d. from three samples.

## Data Availability

Supplementary Materials are available online. Figures S1–S6: X-ray structures of compounds **1**, **2**, **3**, **Ph3C2B**, **Ph3C2BCr2** and **Ph3C2BCr3**, Tables S1–S6. Detailed information on the structural determinations and structural features of compounds **1**, **2**, **3**, **Ph3C2B**, **Ph3C2BCr2** and **Ph3C2BCr3** are provided in the Supplementary Materials.

## Supplementary Materials

The following supporting information can be downloaded at: https://www.mdpi.com/article/10.3390/molecules28134942/s1, Figures S1–S9: NMR spectra of compounds 1, 2, and 3; Figures S10–S12: Molecular structures of **Ph3C2B**, **Ph3C2BCr2**, and **Ph3C2BCr3**; Tables S1–S9: Bond lengths (Å), angles (°), and torsion angles (°) of compounds **1**, **2**, and **3**; Tables S10 and S11: Crystal data and structure refinement and comparison of selected bond lengths (Å), angles (°), and torsion angles (°) for **1**–**3** and **Ph3C2B**, **Ph3C2BCr2**, and **Ph3C2BCr3**.

## Appendix A

CCDC 2262119, 2262120, and 2262121 contain supplementary crystallographic data for compounds **1**, **2**, and **3**, respectively. These data can be obtained free of charge via www.ccdc.cam.ac.uk/conts/retrieving.html, (or from the Cambridge Crystallographic Data Centre, 12, Union Road, Cambridge CB2 1EZ, UK; Fax: +44-1223-336033; or deposit@ccdc.cam.ac.uk). Additional Supporting Information can be found online in the Supporting Information tab.
