# Supplementary material for: 1,2-Diphenyl-o-carborane and Its Chromium Derivatives: Synthesis, Characterization, X-ray Structural Studies, and Biological Evaluations
Source: Molecules. 2023 Jun 23;28(13):4942. doi: 10.3390/molecules28134942 (PMC10343451; doi:10.3390/molecules28134942)
Supplement: Supplementary file 1 [file molecules-28-04942-s001.zip › molecules-2454834-supplementary.pdf]

Supplementary Material

1,2-Diphenyl-o-carborane and its Chromium Derivatives: Synthesis, Characterization, X-ray Structural Studies, and Biological Evaluations

1. NMR spectra of compounds **1**, **2**, and **3** S2
2. Bond lengths, angles, and torsion angles of compound **1**. S7
3. Bond lengths, angles, and torsion angles of compound **2**. S22
4. Bond lengths, angles, and torsion angles of compound **3**. S33
5. Molecular structure of **Ph3C2B**. S79
6. Molecular structure of **Ph3C2BCr2**. S80
7. Molecular structure of **Ph3C2BCr3**. S81
8. Crystal data and structure refinement of **1** ~ **3**, **Ph3C2B**, **Ph3C2BCr2**, and **Ph3C2BCr3**. S82
9. Comparison of selected bond lengths (Å), angles (º), and torsion angles (º) for **1** ~ **3**, **Ph3C2B**, **Ph3C2BCr2**, and **Ph3C2BCr3**. S84

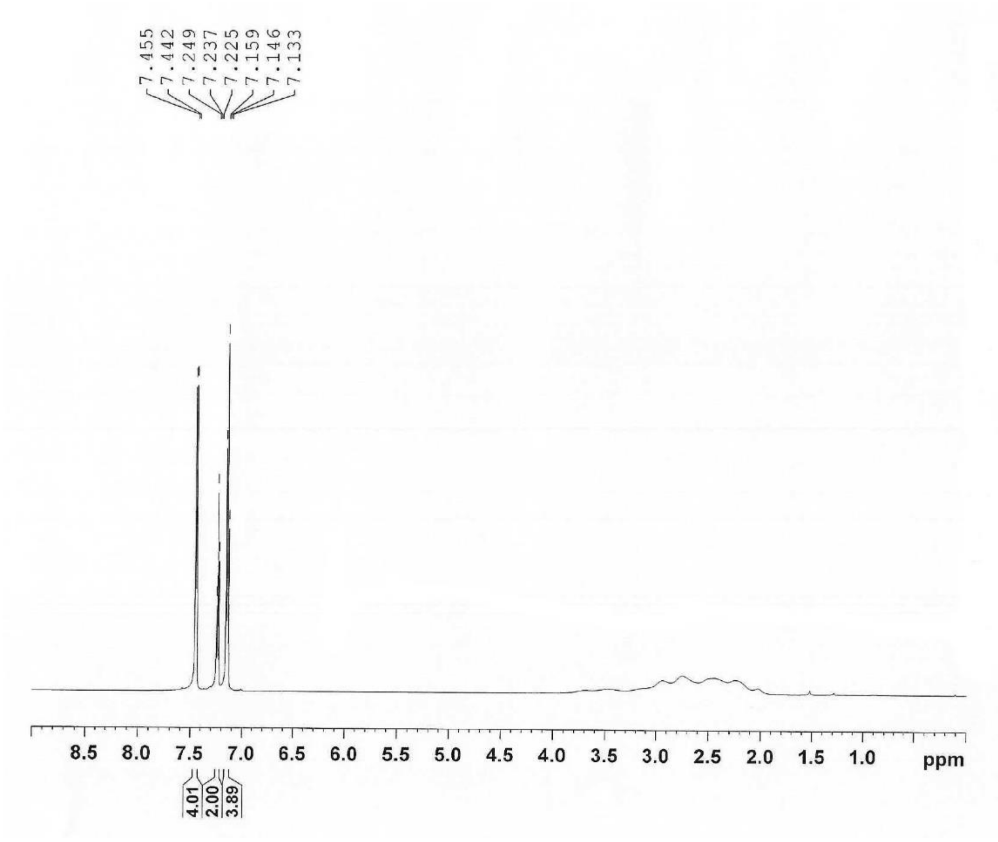

Figure S1. ^{1}H NMR spectrum of **1**.

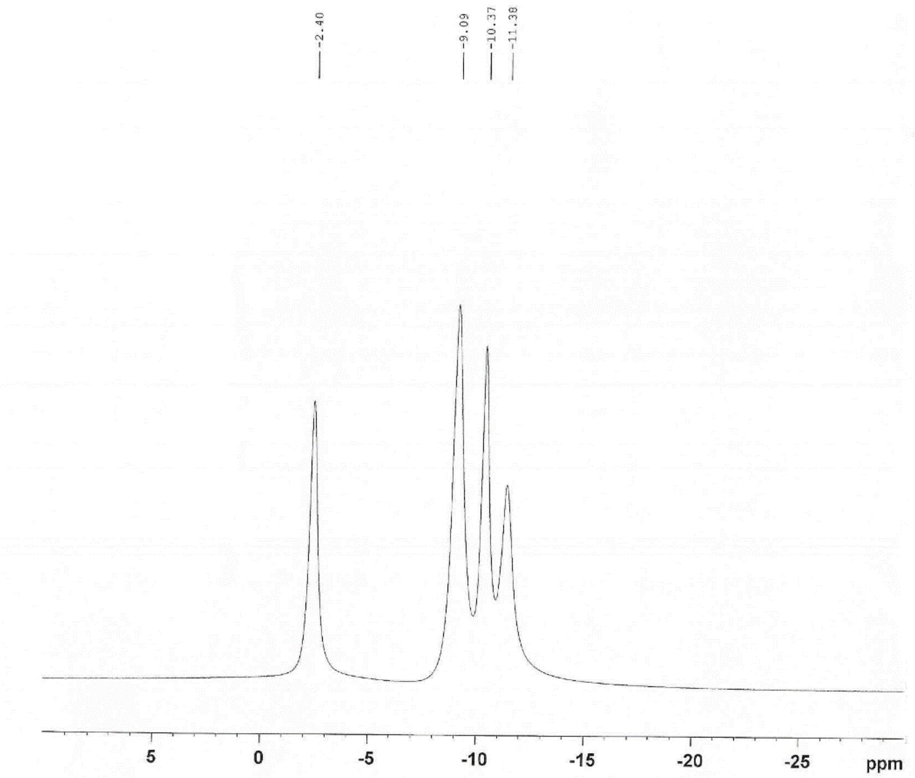

Figure S2. ^{11}B NMR spectrum of **1**.

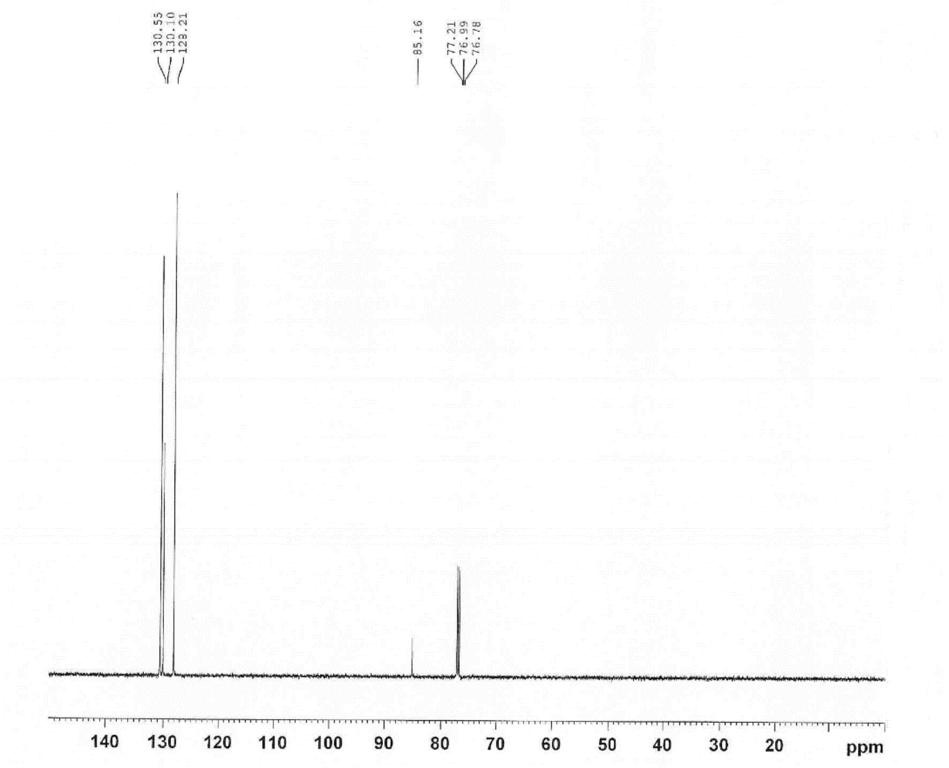

Figure S3. ^{13}C NMR spectrum of **1**.

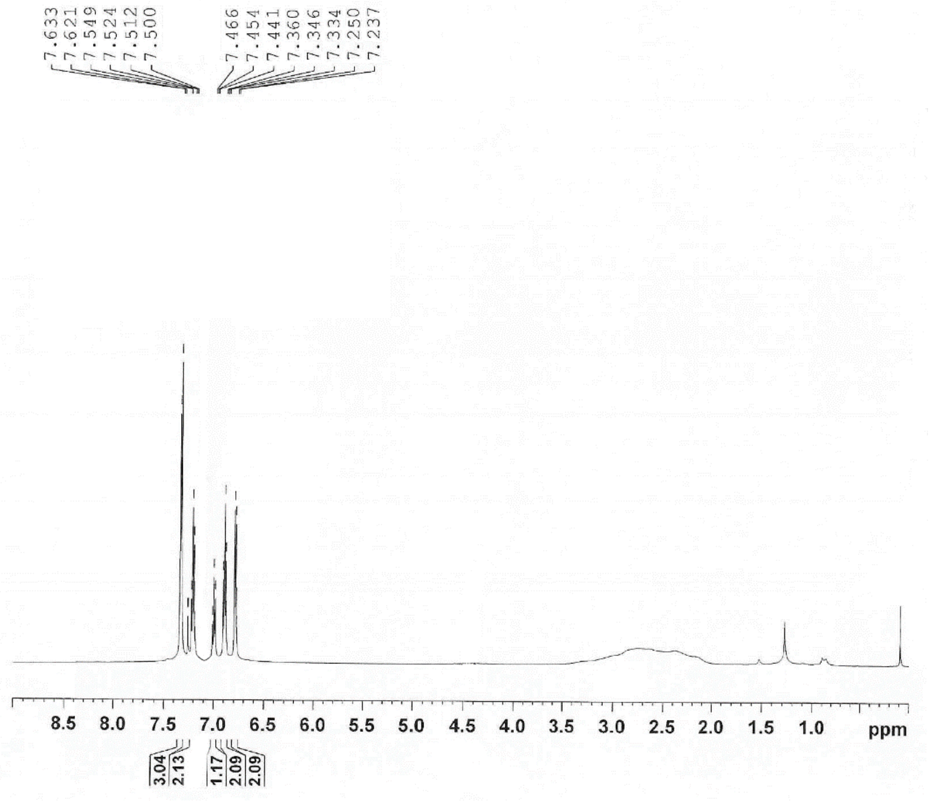

Figure S4. ^{1}H NMR spectrum of **2**.

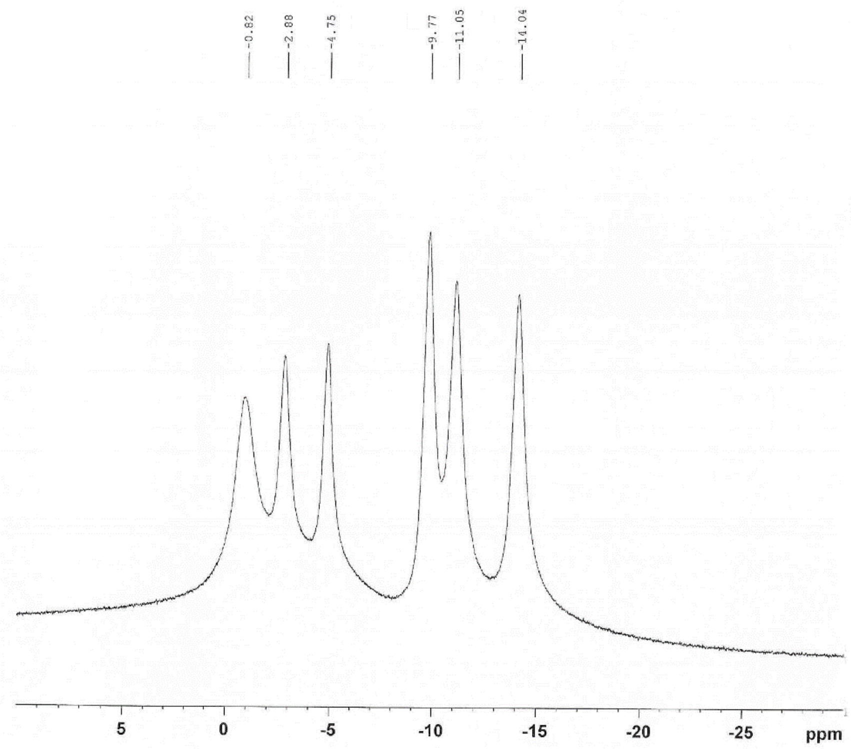

Figure S5. ^{11}B NMR spectrum of **2**.

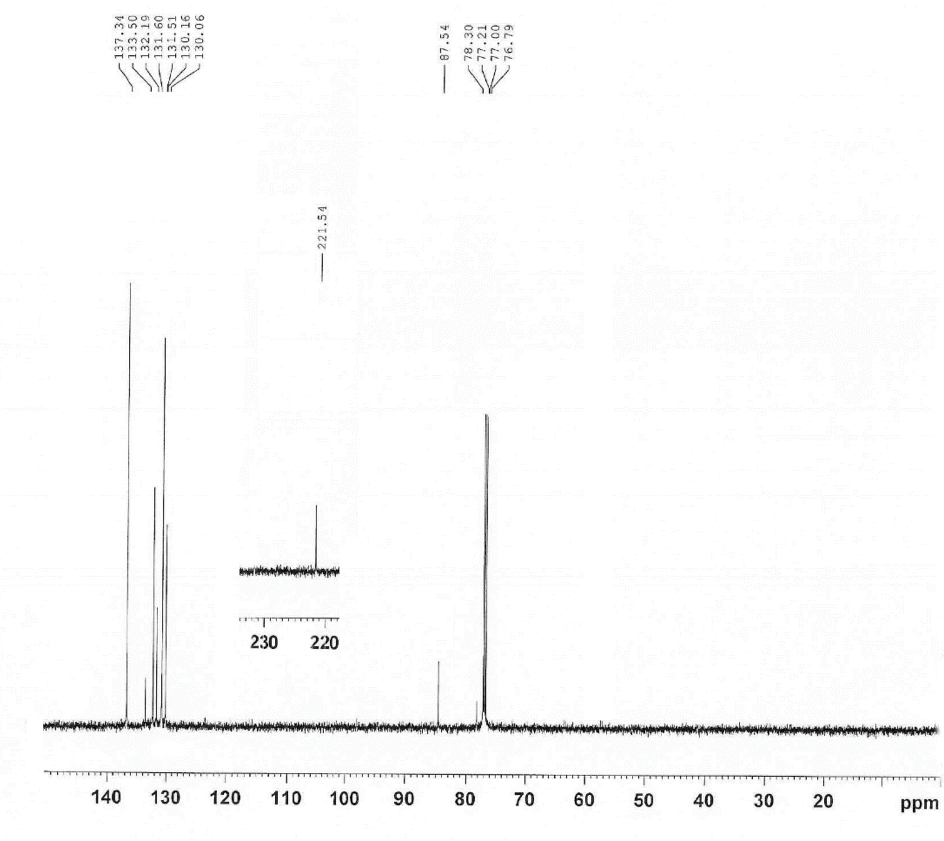

Figure S6. ^{13}C NMR spectrum of **2**.

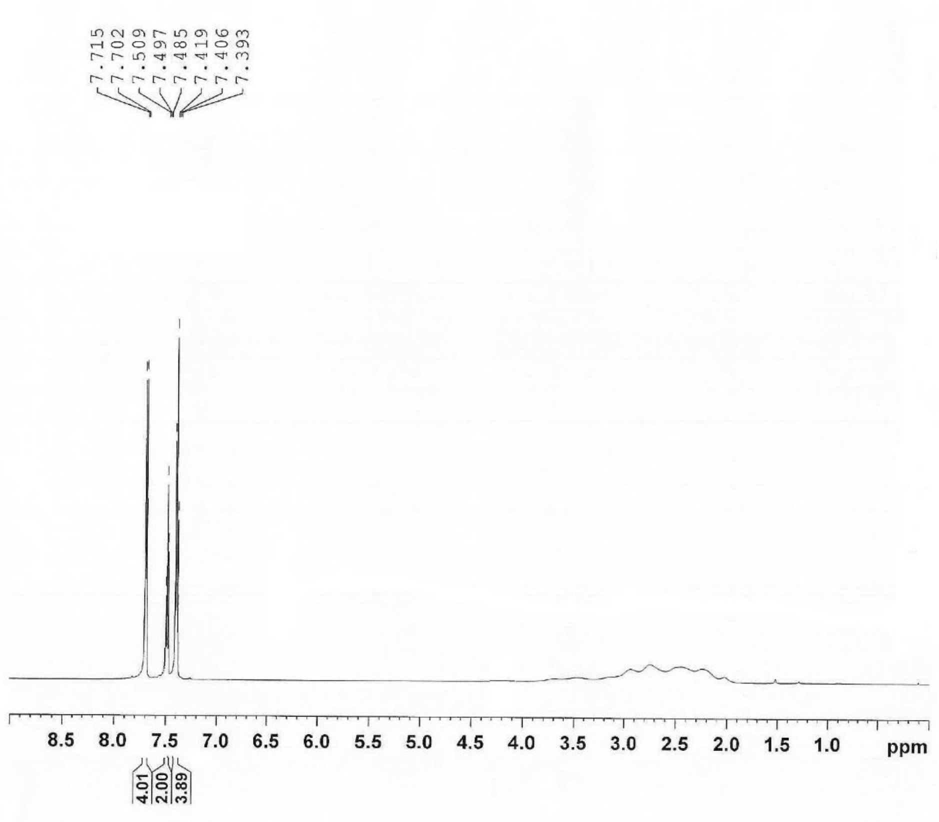

Figure S7. ^{1}H NMR spectrum of **3**.

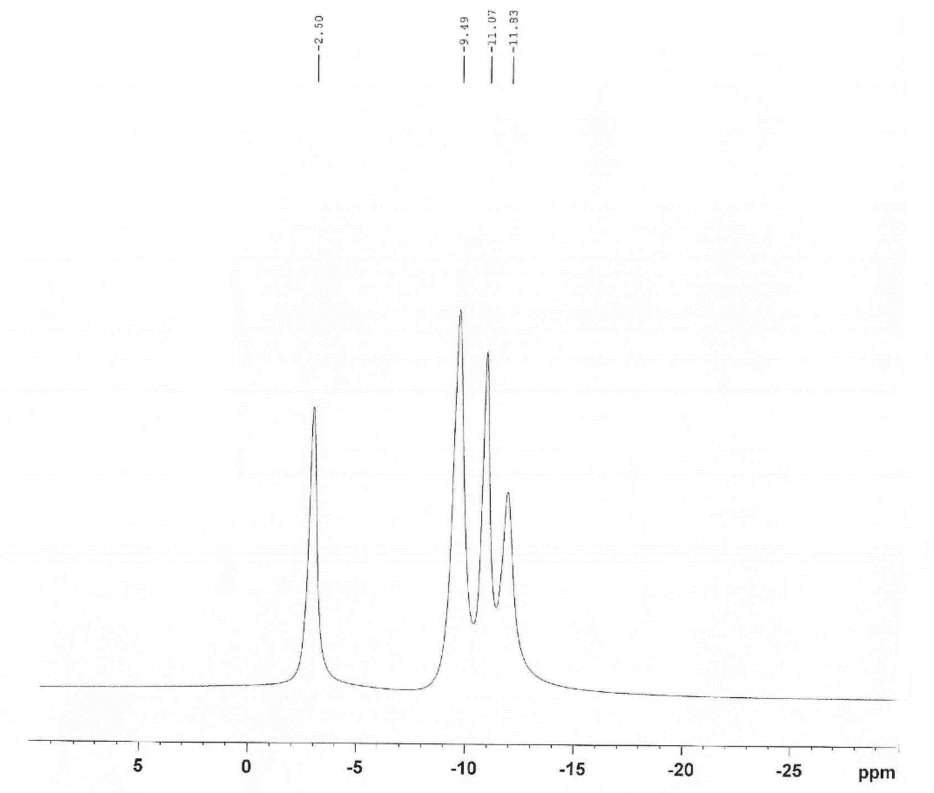

Figure S8. ^{11}B NMR spectrum of **3**.

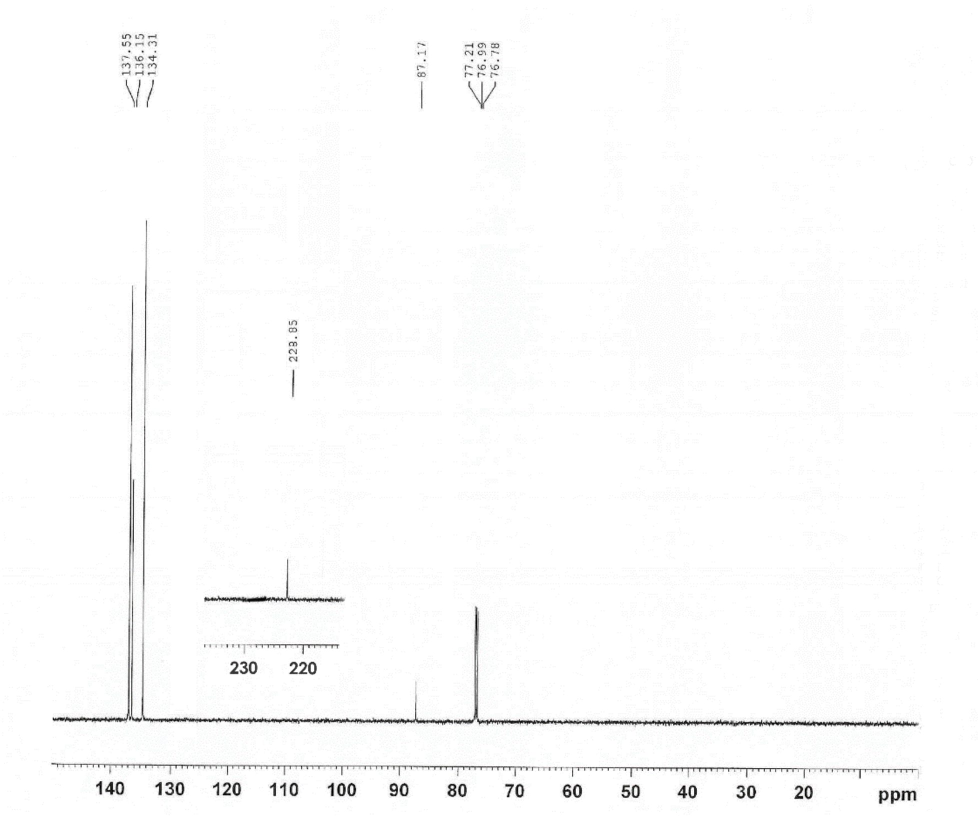

Figure S9. ^{13}C NMR spectrum of **3**.

Table S1. Bond lengths (Å) of **1**.

B3 C1	1.725(2)	B3A C1A	1.725(2)
B3 C2	1.730(2)	B3A C2A	1.728(3)
B3 B9	1.763(3)	B3A B9A	1.759(3)
B3 B8	1.775(3)	B3A B8A	1.773(3)
B3 B4	1.776(3)	B3A B4A	1.779(3)
B4 C1	1.709(2)	B4A C1A	1.708(3)
B4 B9	1.772(3)	B4A B10A	1.773(3)
B4 B5	1.775(3)	B4A B5A	1.773(3)
B4 B10	1.778(3)	B4A B9A	1.774(3)
B5 C1	1.715(2)	B5A C1A	1.706(3)
B5 B6	1.766(3)	B5A B11A	1.773(3)
B5 B10	1.773(3)	B5A B10A	1.776(3)
B5 B11	1.779(3)	B5A B6A	1.776(3)
B6 C2	1.726(2)	B6A C1A	1.724(2)
B6 C1	1.727(2)	B6A C2A	1.729(3)
B6 B11	1.764(3)	B6A B11A	1.761(3)
B6 B7	1.780(3)	B6A B7A	1.771(3)
B7 C2	1.714(2)	B7A C2A	1.711(3)
B7 B8	1.773(3)	B7A B8A	1.774(3)
B7 B12	1.780(3)	B7A B12A	1.774(4)
B7 B11	1.782(3)	B7A B11A	1.774(3)
B8 C2	1.712(2)	B8A C2A	1.713(3)
B8 B9	1.776(3)	B8A B9A	1.770(4)
B8 B12	1.777(3)	B8A B12A	1.773(4)
B9 B10	1.774(3)	B9A B12A	1.770(4)
B9 B12	1.775(3)	B9A B10A	1.777(3)
B10 B12	1.774(3)	B10A B12A	1.769(4)
B10 B11	1.776(3)	B10A B11A	1.776(3)
B11 B12	1.776(3)	B11A B12A	1.779(3)
C1 C13	1.507(2)	C1A C13A	1.504(2)
C1 C2	1.726(2)	C1A C2A	1.737(2)
C2 C19	1.501(2)	C2A C19A	1.510(2)
C13 C14	1.379(3)	C13A C18A	1.383(3)
C13 C18	1.380(3)	C13A C14A	1.386(3)
C14 C15	1.386(3)	C14A C15A	1.388(3)
C15 C16	1.362(4)	C15A C16A	1.366(4)
C16 C17	1.363(4)	C16A C17A	1.362(4)
C17 C18	1.382(3)	C17A C18A	1.392(3)
C19 C24	1.378(3)	C19A C20A	1.379(3)
C19 C20	1.380(3)	C19A C24A	1.383(3)
C20 C21	1.381(3)	C20A C21A	1.393(3)
C21 C22	1.360(4)	C21A C22A	1.357(4)
C22 C23	1.367(4)	C22A C23A	1.361(4)
C23 C24	1.393(3)	C23A C24A	1.397(3)

Table S2. Bond angles (º) of **1**.

C1 B3 C2	59.95(9)	C1A B3A C2A	60.40(10)
C1 B3 B9	105.53(14)	C1A B3A B9A	105.52(14)
C2 B3 B9	105.62(13)	C2A B3A B9A	105.87(15)
C1 B3 B8	106.77(13)	C1A B3A B8A	106.98(14)
C2 B3 B8	58.45(10)	C2A B3A B8A	58.57(12)
B9 B3 B8	60.27(11)	B9A B3A B8A	60.13(14)
C1 B3 B4	58.40(10)	C1A B3A B4A	58.29(10)
C2 B3 B4	106.69(13)	C2A B3A B4A	107.17(14)
B9 B3 B4	60.08(12)	B9A B3A B4A	60.16(13)
B8 B3 B4	108.60(14)	B8A B3A B4A	108.64(16)
C1 B4 B9	105.86(13)	C1A B4A B10A	105.53(15)
C1 B4 B5	58.94(11)	C1A B4A B5A	58.64(11)
B9 B4 B5	107.76(15)	B10A B4A B5A	60.11(13)
C1 B4 B3	59.30(10)	C1A B4A B9A	105.65(15)
B9 B4 B3	59.60(11)	B10A B4A B9A	60.16(13)
B5 B4 B3	107.60(14)	B5A B4A B9A	108.05(16)
C1 B4 B10	105.70(14)	C1A B4A B3A	59.27(10)
B9 B4 B10	59.96(13)	B10A B4A B3A	107.35(15)
B5 B4 B10	59.87(13)	B5A B4A B3A	107.45(14)
B3 B4 B10	107.45(14)	B9A B4A B3A	59.36(12)
C1 B5 B6	59.50(10)	C1A B5A B11A	105.85(14)
C1 B5 B10	105.66(14)	C1A B5A B4A	58.75(11)
B6 B5 B10	107.46(15)	B11A B5A B4A	107.98(15)
C1 B5 B4	58.60(10)	C1A B5A B10A	105.48(14)
B6 B5 B4	107.57(14)	B11A B5A B10A	60.06(13)
B10 B5 B4	60.16(12)	B4A B5A B10A	59.93(12)
C1 B5 B11	106.15(14)	C1A B5A B6A	59.31(11)
B6 B5 B11	59.67(12)	B11A B5A B6A	59.48(12)
B10 B5 B11	60.00(13)	B4A B5A B6A	107.49(14)
B4 B5 B11	108.13(15)	B10A B5A B6A	107.26(15)
C2 B6 C1	59.99(9)	C1A B6A C2A	60.40(10)
C2 B6 B11	106.05(14)	C1A B6A B11A	105.58(15)
C1 B6 B11	106.28(14)	C2A B6A B11A	106.08(15)
C2 B6 B5	107.21(13)	C1A B6A B7A	106.94(14)
C1 B6 B5	58.79(10)	C2A B6A B7A	58.53(11)
B11 B6 B5	60.54(12)	B11A B6A B7A	60.32(13)
C2 B6 B7	58.50(10)	C1A B6A B5A	58.29(11)
C1 B6 B7	107.02(13)	C2A B6A B5A	107.23(14)
B11 B6 B7	60.38(12)	B11A B6A B5A	60.16(13)
B5 B6 B7	109.13(14)	B7A B6A B5A	108.71(16)
C2 B7 B8	58.78(10)	C2A B7A B6A	59.50(11)
C2 B7 B6	59.17(10)	C2A B7A B8A	58.85(11)
B8 B7 B6	107.17(13)	B6A B7A B8A	107.65(15)
C2 B7 B12	105.54(14)	C2A B7A B12A	105.79(16)
B8 B7 B12	60.00(12)	B6A B7A B12A	107.45(16)
B6 B7 B12	106.85(14)	B8A B7A B12A	59.97(14)
C2 B7 B11	105.76(13)	C2A B7A B11A	106.24(15)

B8 B7 B11	107.78(14)	B6A B7A B11A	59.56(13)
B6 B7 B11	59.36(11)	B8A B7A B11A	108.17(17)
B12 B7 B11	59.82(12)	B12A B7A B11A	60.18(14)
C2 B8 B7	58.88(10)	C2A B8A B9A	106.03(15)
C2 B8 B3	59.46(10)	C2A B8A B12A	105.75(15)
B7 B8 B3	107.94(13)	B9A B8A B12A	59.95(15)
C2 B8 B9	105.83(13)	C2A B8A B3A	59.38(11)
B7 B8 B9	108.10(15)	B9A B8A B3A	59.54(13)
B3 B8 B9	59.53(11)	B12A B8A B3A	107.28(17)
C2 B8 B12	105.78(14)	C2A B8A B7A	58.74(11)
B7 B8 B12	60.21(12)	B9A B8A B7A	107.85(17)
B3 B8 B12	107.56(14)	B12A B8A B7A	60.02(14)
B9 B8 B12	59.96(12)	B3A B8A B7A	107.37(14)
C2 B8 H8	116.9(9)	B3A B9A B8A	60.33(13)
B3 B9 B4	60.32(11)	B3A B9A B12A	108.04(16)
B3 B9 B10	108.22(14)	B8A B9A B12A	60.12(14)
B4 B9 B10	60.20(12)	B3A B9A B4A	60.48(12)
B3 B9 B12	108.15(14)	B8A B9A B4A	109.05(15)
B4 B9 B12	108.44(15)	B12A B9A B4A	108.09(16)
B10 B9 B12	59.97(13)	B3A B9A B10A	108.03(15)
B3 B9 B8	60.20(11)	B8A B9A B10A	108.28(17)
B4 B9 B8	108.73(14)	B12A B9A B10A	59.81(14)
B10 B9 B8	108.17(15)	B4A B9A B10A	59.89(13)
B12 B9 B8	60.02(12)	B12A B10A B4A	108.21(16)
B5 B10 B12	108.28(15)	B12A B10A B5A	108.29(16)
B5 B10 B9	107.74(15)	B4A B10A B5A	59.96(12)
B12 B10 B9	60.06(13)	B12A B10A B11A	60.25(14)
B5 B10 B11	60.16(12)	B4A B10A B11A	107.87(15)
B12 B10 B11	60.04(13)	B5A B10A B11A	59.89(13)
B9 B10 B11	107.83(15)	B12A B10A B9A	59.90(14)
B5 B10 B4	59.97(12)	B4A B10A B9A	59.95(13)
B12 B10 B4	108.21(15)	B5A B10A B9A	107.77(15)
B9 B10 B4	59.84(12)	B11A B10A B9A	107.74(17)
B11 B10 B4	108.11(14)	B6A B11A B5A	60.36(12)
B6 B11 B10	107.41(14)	B6A B11A B7A	60.12(12)
B6 B11 B12	107.75(15)	B5A B11A B7A	108.70(15)
B10 B11 B12	59.91(13)	B6A B11A B10A	107.94(15)
B6 B11 B5	59.78(12)	B5A B11A B10A	60.05(13)
B10 B11 B5	59.84(12)	B7A B11A B10A	107.95(17)
B12 B11 B5	107.91(15)	B6A B11A B12A	107.67(16)
B6 B11 B7	60.26(11)	B5A B11A B12A	107.95(16)
B10 B11 B7	108.11(16)	B7A B11A B12A	59.90(14)
B12 B11 B7	60.05(12)	B10A B11A B12A	59.67(14)
B5 B11 B7	108.43(14)	B10A B12A B9A	60.29(14)
B10 B12 B9	59.97(13)	B10A B12A B8A	108.51(16)
B10 B12 B11	60.04(13)	B9A B12A B8A	59.93(14)
B9 B12 B11	107.76(15)	B10A B12A B7A	108.28(16)
B10 B12 B8	108.17(15)	B9A B12A B7A	107.83(16)
B9 B12 B8	60.02(12)	B8A B12A B7A	60.02(13)

B11 B12 B8	107.87(14)	B10A B12A B11A	60.08(14)
B10 B12 B7	108.29(15)	B9A B12A B11A	107.92(16)
B9 B12 B7	107.81(14)	B8A B12A B11A	107.99(16)
B11 B12 B7	60.14(12)	B7A B12A B11A	59.91(14)
B8 B12 B7	59.79(11)	C13A C1A B5A	120.48(13)
C13 C1 B4	122.54(13)	C13A C1A B4A	120.73(14)
C13 C1 B5	121.65(13)	B5A C1A B4A	62.61(12)
B4 C1 B5	62.46(12)	C13A C1A B6A	118.49(14)
C13 C1 B3	118.84(13)	B5A C1A B6A	62.39(12)
B4 C1 B3	62.29(11)	B4A C1A B6A	113.06(14)
B5 C1 B3	112.83(13)	C13A C1A B3A	118.73(13)
C13 C1 C2	118.31(12)	B5A C1A B3A	113.19(13)
B4 C1 C2	109.96(12)	B4A C1A B3A	62.44(11)
B5 C1 C2	109.51(12)	B6A C1A B3A	110.89(13)
B3 C1 C2	60.18(9)	C13A C1A C2A	119.73(12)
C13 C1 B6	117.57(13)	B5A C1A C2A	110.12(13)
B4 C1 B6	112.48(13)	B4A C1A C2A	110.05(12)
B5 C1 B6	61.71(11)	B6A C1A C2A	59.93(10)
B3 C1 B6	111.00(12)	B3A C1A C2A	59.87(10)
C2 C1 B6	59.96(10)	C19A C2A B7A	121.64(14)
C19 C2 B8	121.61(13)	C19A C2A B8A	121.76(15)
C19 C2 B7	121.36(13)	B7A C2A B8A	62.41(13)
B8 C2 B7	62.34(11)	C19A C2A B3A	118.77(14)
C19 C2 B6	118.61(13)	B7A C2A B3A	112.46(14)
B8 C2 B6	112.54(13)	B8A C2A B3A	62.05(12)
B7 C2 B6	62.32(11)	C19A C2A B6A	118.47(15)
C19 C2 C1	118.99(12)	B7A C2A B6A	61.96(12)
B8 C2 C1	109.60(12)	B8A C2A B6A	112.49(14)
B7 C2 C1	110.12(12)	B3A C2A B6A	110.54(13)
B6 C2 C1	60.05(10)	C19A C2A C1A	119.56(13)
C19 C2 B3	118.35(13)	B7A C2A C1A	109.07(13)
B8 C2 B3	62.09(11)	B8A C2A C1A	109.21(13)
B7 C2 B3	112.85(13)	B3A C2A C1A	59.73(10)
B6 C2 B3	110.82(12)	B6A C2A C1A	59.66(10)
C1 C2 B3	59.87(9)	C18A C13A C14A	118.55(17)
C14 C13 C18	118.06(17)	C18A C13A C1A	120.72(16)
C14 C13 C1	121.72(16)	C14A C13A C1A	120.49(16)
C18 C13 C1	120.20(16)	C13A C14A C15A	120.2(2)
C13 C14 C15	120.5(2)	C17A C16A C15A	119.8(2)
C15 C16 C17	119.2(2)	C16A C17A C18A	120.4(2)
C16 C17 C18	120.7(2)	C13A C18A C17A	120.4(2)
C24 C19 C20	118.71(19)	C20A C19A C24A	118.2(2)
C24 C19 C2	120.75(17)	C20A C19A C2A	121.45(17)
C20 C19 C2	120.48(17)	C24A C19A C2A	120.23(19)
C19 C20 C21	120.7(3)	C19A C20A C21A	120.8(2)
C22 C21 C20	120.4(3)	C22A C21A C20A	120.5(3)
C21 C22 C23	119.7(2)	C21A C22A C23A	119.5(2)
C22 C23 C24	120.6(3)	C22A C23A C24A	120.8(3)
C19 C24 C23	119.9(2)	C19A C24A C23A	120.1(3)

Table S3. Torsional angles (º) of **1**.

C2 B3 B4 C1	37.05(12)	C2A B3A B4A C1A	37.01(12)
B9 B3 B4 C1	135.78(14)	B9A B3A B4A C1A	135.80(16)
B8 B3 B4 C1	98.65(14)	B8A B3A B4A C1A	98.86(15)
C1 B3 B4 B9	-135.78(14)	C1A B3A B4A B10A	-98.09(15)
C2 B3 B4 B9	-98.74(15)	C2A B3A B4A B10A	-61.08(17)
B8 B3 B4 B9	-37.13(14)	B9A B3A B4A B10A	37.71(15)
C1 B3 B4 B5	-35.13(12)	B8A B3A B4A B10A	0.77(19)
C2 B3 B4 B5	1.92(18)	C1A B3A B4A B5A	-34.77(13)
B9 B3 B4 B5	100.65(16)	C2A B3A B4A B5A	2.24(18)
B8 B3 B4 B5	63.52(17)	B9A B3A B4A B5A	101.03(17)
C1 B3 B4 B10	-98.24(15)	B8A B3A B4A B5A	64.09(18)
C2 B3 B4 B10	-61.19(17)	C1A B3A B4A B9A	-135.80(16)
B9 B3 B4 B10	37.54(14)	C2A B3A B4A B9A	-98.79(16)
B8 B3 B4 B10	0.42(18)	B8A B3A B4A B9A	-36.94(15)
B9 B4 B5 C1	98.16(14)	B10A B4A B5A C1A	135.40(15)
B3 B4 B5 C1	35.28(12)	B9A B4A B5A C1A	97.68(16)
B10 B4 B5 C1	135.63(14)	B3A B4A B5A C1A	35.03(13)
C1 B4 B5 B6	-35.20(13)	C1A B4A B5A B11A	-97.98(15)
B9 B4 B5 B6	62.96(18)	B10A B4A B5A B11A	37.42(15)
B3 B4 B5 B6	0.08(19)	B9A B4A B5A B11A	-0.3(2)
B10 B4 B5 B6	100.43(16)	B3A B4A B5A B11A	-62.95(18)
C1 B4 B5 B10	-135.63(14)	C1A B4A B5A B10A	-135.40(15)
B9 B4 B5 B10	-37.47(14)	B9A B4A B5A B10A	-37.72(15)
B3 B4 B5 B10	-100.35(15)	B3A B4A B5A B10A	-100.37(16)
C1 B4 B5 B11	-98.22(15)	C1A B4A B5A B6A	-35.21(13)
B9 B4 B5 B11	-0.06(19)	B10A B4A B5A B6A	100.19(16)
B3 B4 B5 B11	-62.94(18)	B9A B4A B5A B6A	62.47(18)
B10 B4 B5 B11	37.41(14)	B3A B4A B5A B6A	-0.18(19)
C1 B5 B6 C2	-36.88(12)	B11A B5A B6A C1A	135.92(15)
B10 B5 B6 C2	61.35(17)	B4A B5A B6A C1A	34.97(13)
B4 B5 B6 C2	-2.06(18)	B10A B5A B6A C1A	98.08(15)
B11 B5 B6 C2	99.06(15)	C1A B5A B6A C2A	-36.92(12)
B10 B5 B6 C1	98.23(15)	B11A B5A B6A C2A	99.00(16)
B4 B5 B6 C1	34.82(12)	B4A B5A B6A C2A	-1.95(19)
B11 B5 B6 C1	135.94(15)	B10A B5A B6A C2A	61.16(18)
C1 B5 B6 B11	-135.94(15)	C1A B5A B6A B11A	-135.92(15)
B10 B5 B6 B11	-37.71(14)	B4A B5A B6A B11A	-100.95(16)
B4 B5 B6 B11	-101.12(16)	B10A B5A B6A B11A	-37.84(15)
C1 B5 B6 B7	-98.75(14)	C1A B5A B6A B7A	-98.76(15)
B10 B5 B6 B7	-0.52(19)	B11A B5A B6A B7A	37.16(15)
B4 B5 B6 B7	-63.93(17)	B4A B5A B6A B7A	-63.79(18)
B11 B5 B6 B7	37.19(14)	B10A B5A B6A B7A	-0.68(19)
C1 B6 B7 C2	36.77(12)	C1A B6A B7A C2A	37.66(13)
B11 B6 B7 C2	136.16(14)	B11A B6A B7A C2A	136.27(16)
B5 B6 B7 C2	98.90(14)	B5A B6A B7A C2A	99.17(15)
C2 B6 B7 B8	-35.30(12)	C1A B6A B7A B8A	2.5(2)
C1 B6 B7 B8	1.47(18)	C2A B6A B7A B8A	-35.17(14)

B11 B6 B7 B8	100.86(15)	B11A B6A B7A B8A	101.10(18)
B5 B6 B7 B8	63.60(17)	B5A B6A B7A B8A	64.01(19)
C2 B6 B7 B12	-98.35(15)	C1A B6A B7A B12A	-60.73(19)
C1 B6 B7 B12	-61.58(17)	C2A B6A B7A B12A	-98.39(17)
B11 B6 B7 B12	37.81(14)	B11A B6A B7A B12A	37.88(15)
B5 B6 B7 B12	0.55(19)	B5A B6A B7A B12A	0.8(2)
C2 B6 B7 B11	-136.16(14)	C1A B6A B7A B11A	-98.61(16)
C1 B6 B7 B11	-99.39(15)	C2A B6A B7A B11A	-136.27(16)
B5 B6 B7 B11	-37.26(14)	B5A B6A B7A B11A	-37.09(15)
B6 B7 B8 C2	35.47(12)	C1A B3A B8A C2A	-37.63(13)
B12 B7 B8 C2	135.37(14)	B9A B3A B8A C2A	-136.10(15)
B11 B7 B8 C2	98.01(14)	B4A B3A B8A C2A	-99.15(15)
C2 B7 B8 B3	-34.99(12)	C1A B3A B8A B9A	98.46(16)
B6 B7 B8 B3	0.48(18)	C2A B3A B8A B9A	136.10(15)
B12 B7 B8 B3	100.38(15)	B4A B3A B8A B9A	36.95(15)
B11 B7 B8 B3	63.01(17)	C1A B3A B8A B12A	60.77(19)
C2 B7 B8 B9	-97.93(14)	C2A B3A B8A B12A	98.41(17)
B6 B7 B8 B9	-62.46(17)	B9A B3A B8A B12A	-37.69(16)
B12 B7 B8 B9	37.44(14)	B4A B3A B8A B12A	-0.7(2)
B11 B7 B8 B9	0.08(18)	C1A B3A B8A B7A	-2.4(2)
C2 B7 B8 B12	-135.37(14)	C2A B3A B8A B7A	35.22(14)
B6 B7 B8 B12	-99.90(15)	B9A B3A B8A B7A	-100.88(18)
B11 B7 B8 B12	-37.36(13)	B4A B3A B8A B7A	-63.93(19)
C1 B3 B8 C2	-36.99(12)	B6A B7A B8A C2A	35.44(14)
B9 B3 B8 C2	-135.61(14)	B12A B7A B8A C2A	135.79(16)
B4 B3 B8 C2	-98.57(14)	B11A B7A B8A C2A	98.37(16)
C1 B3 B8 B7	-2.24(18)	C2A B7A B8A B9A	-98.27(16)
C2 B3 B8 B7	34.75(12)	B6A B7A B8A B9A	-62.83(19)
B9 B3 B8 B7	-100.86(16)	B12A B7A B8A B9A	37.52(15)
B4 B3 B8 B7	-63.81(17)	B11A B7A B8A B9A	0.1(2)
C1 B3 B8 B9	98.63(15)	C2A B7A B8A B12A	-135.79(16)
C2 B3 B8 B9	135.61(14)	B6A B7A B8A B12A	-100.35(18)
B4 B3 B8 B9	37.05(14)	B11A B7A B8A B12A	-37.42(15)
C1 B3 B8 B12	61.32(16)	C2A B7A B8A B3A	-35.49(14)
C2 B3 B8 B12	98.31(14)	B6A B7A B8A B3A	0.0(2)
B9 B3 B8 B12	-37.30(14)	B12A B7A B8A B3A	100.30(18)
B4 B3 B8 B12	-0.26(18)	B11A B7A B8A B3A	62.9(2)
C1 B3 B9 B4	38.06(12)	C1A B3A B9A B8A	-100.96(15)
C2 B3 B9 B4	100.55(14)	C2A B3A B9A B8A	-37.97(14)
B8 B3 B9 B4	138.79(15)	B4A B3A B9A B8A	-138.96(16)
C1 B3 B9 B10	0.22(18)	C1A B3A B9A B12A	-63.01(19)
C2 B3 B9 B10	62.71(17)	C2A B3A B9A B12A	0.0(2)
B8 B3 B9 B10	100.95(16)	B8A B3A B9A B12A	37.95(16)
B4 B3 B9 B10	-37.84(14)	B4A B3A B9A B12A	-101.00(17)
C1 B3 B9 B12	-63.25(17)	C1A B3A B9A B4A	37.99(13)
C2 B3 B9 B12	-0.77(18)	C2A B3A B9A B4A	100.99(15)
B8 B3 B9 B12	37.47(14)	B8A B3A B9A B4A	138.96(16)
B4 B3 B9 B12	-101.32(16)	C1A B3A B9A B10A	0.2(2)
C1 B3 B9 B8	-100.73(14)	C2A B3A B9A B10A	63.23(19)

C2 B3 B9 B8	-38.24(12)	B8A B3A B9A B10A	101.20(18)
B4 B3 B9 B8	-138.79(15)	B4A B3A B9A B10A	-37.76(15)
C1 B4 B9 B3	-38.56(13)	C2A B8A B9A B3A	38.38(13)
B5 B4 B9 B3	-100.39(15)	B12A B8A B9A B3A	137.59(16)
B10 B4 B9 B3	-137.82(15)	B7A B8A B9A B3A	100.05(16)
C1 B4 B9 B10	99.25(15)	C2A B8A B9A B12A	-99.21(17)
B5 B4 B9 B10	37.43(14)	B3A B8A B9A B12A	-137.59(16)
B3 B4 B9 B10	137.82(15)	B7A B8A B9A B12A	-37.54(15)
C1 B4 B9 B12	62.27(18)	C2A B8A B9A B4A	1.2(2)
B5 B4 B9 B12	0.44(19)	B12A B8A B9A B4A	100.40(18)
B3 B4 B9 B12	100.83(15)	B3A B8A B9A B4A	-37.19(15)
B10 B4 B9 B12	-36.99(14)	B7A B8A B9A B4A	62.9(2)
C1 B4 B9 B8	-1.4(2)	C2A B8A B9A B10A	-62.39(19)
B5 B4 B9 B8	-63.25(19)	B12A B8A B9A B10A	36.81(15)
B3 B4 B9 B8	37.13(14)	B3A B8A B9A B10A	-100.78(16)
B10 B4 B9 B8	-100.68(17)	B7A B8A B9A B10A	-0.7(2)
C2 B8 B9 B3	38.77(13)	C1A B4A B9A B3A	-38.49(14)
B7 B8 B9 B3	100.58(14)	B10A B4A B9A B3A	-137.70(16)
B12 B8 B9 B3	138.13(15)	B5A B4A B9A B3A	-99.99(15)
C2 B8 B9 B4	1.6(2)	C1A B4A B9A B8A	-1.4(2)
B7 B8 B9 B4	63.40(19)	B10A B4A B9A B8A	-100.57(18)
B3 B8 B9 B4	-37.18(14)	B5A B4A B9A B8A	-62.9(2)
B12 B8 B9 B4	100.95(17)	B3A B4A B9A B8A	37.13(15)
C2 B8 B9 B10	-62.25(18)	C1A B4A B9A B12A	62.4(2)
B7 B8 B9 B10	-0.44(19)	B10A B4A B9A B12A	-36.77(16)
B3 B8 B9 B10	-101.02(15)	B5A B4A B9A B12A	0.9(2)
B12 B8 B9 B10	37.11(14)	B3A B4A B9A B12A	100.92(18)
C2 B8 B9 B12	-99.36(15)	C1A B4A B9A B10A	99.21(16)
B7 B8 B9 B12	-37.55(14)	B5A B4A B9A B10A	37.70(15)
B3 B8 B9 B12	-138.13(15)	B3A B4A B9A B10A	137.70(16)
C1 B5 B10 B12	62.61(18)	C1A B4A B10A B12A	-62.55(19)
B6 B5 B10 B12	0.3(2)	B5A B4A B10A B12A	-101.04(18)
B4 B5 B10 B12	100.91(17)	B9A B4A B10A B12A	36.84(16)
B11 B5 B10 B12	-37.28(15)	B3A B4A B10A B12A	-0.5(2)
C1 B5 B10 B9	-0.89(19)	C1A B4A B10A B5A	38.49(13)
B6 B5 B10 B9	-63.22(18)	B9A B4A B10A B5A	137.88(17)
B4 B5 B10 B9	37.41(14)	B3A B4A B10A B5A	100.53(15)
B11 B5 B10 B9	-100.78(16)	C1A B4A B10A B11A	1.2(2)
C1 B5 B10 B11	99.89(15)	B5A B4A B10A B11A	-37.31(15)
B6 B5 B10 B11	37.56(14)	B9A B4A B10A B11A	100.57(18)
B4 B5 B10 B11	138.19(15)	B3A B4A B10A B11A	63.22(19)
C1 B5 B10 B4	-38.30(12)	C1A B4A B10A B9A	-99.39(16)
B6 B5 B10 B4	-100.63(15)	B5A B4A B10A B9A	-137.88(17)
B11 B5 B10 B4	-138.19(15)	B3A B4A B10A B9A	-37.35(15)
B3 B9 B10 B5	0.42(19)	C1A B5A B10A B12A	62.37(19)
B4 B9 B10 B5	-37.47(14)	B11A B5A B10A B12A	-37.27(16)
B12 B9 B10 B5	101.28(16)	B4A B5A B10A B12A	100.90(18)
B8 B9 B10 B5	64.15(18)	B6A B5A B10A B12A	0.3(2)
B3 B9 B10 B12	-100.87(15)	C1A B5A B10A B4A	-38.53(13)

B4 B9 B10 B12	-138.76(15)	B11A B5A B10A B4A	-138.17(16)
B8 B9 B10 B12	-37.13(14)	B6A B5A B10A B4A	-100.59(15)
B3 B9 B10 B11	-63.10(18)	C1A B5A B10A B11A	99.64(16)
B4 B9 B10 B11	-100.99(16)	B4A B5A B10A B11A	138.17(16)
B12 B9 B10 B11	37.76(14)	B6A B5A B10A B11A	37.58(14)
B8 B9 B10 B11	0.63(19)	C1A B5A B10A B9A	-1.0(2)
B3 B9 B10 B4	37.89(13)	B11A B5A B10A B9A	-100.61(18)
B12 B9 B10 B4	138.76(15)	B4A B5A B10A B9A	37.56(16)
B8 B9 B10 B4	101.62(15)	B6A B5A B10A B9A	-63.0(2)
C1 B4 B10 B5	38.48(13)	B3A B9A B10A B12A	-100.80(18)
B9 B4 B10 B5	137.99(15)	B8A B9A B10A B12A	-36.95(15)
B3 B4 B10 B5	100.61(15)	B4A B9A B10A B12A	-138.82(16)
C1 B4 B10 B12	-62.54(18)	B3A B9A B10A B4A	38.02(15)
B9 B4 B10 B12	36.97(14)	B8A B9A B10A B4A	101.88(16)
B5 B4 B10 B12	-101.02(17)	B12A B9A B10A B4A	138.82(16)
B3 B4 B10 B12	-0.4(2)	B3A B9A B10A B5A	0.5(2)
C1 B4 B10 B9	-99.51(14)	B8A B9A B10A B5A	64.3(2)
B5 B4 B10 B9	-137.99(15)	B12A B9A B10A B5A	101.25(18)
B3 B4 B10 B9	-37.38(13)	B4A B9A B10A B5A	-37.57(15)
C1 B4 B10 B11	1.00(19)	B3A B9A B10A B11A	-62.8(2)
B9 B4 B10 B11	100.52(17)	B8A B9A B10A B11A	1.1(2)
B5 B4 B10 B11	-37.47(15)	B12A B9A B10A B11A	38.04(15)
B3 B4 B10 B11	63.13(19)	B4A B9A B10A B11A	-100.79(16)
C2 B6 B11 B10	-63.39(18)	C1A B6A B11A B5A	-37.91(13)
C1 B6 B11 B10	-0.66(19)	C2A B6A B11A B5A	-100.96(15)
B5 B6 B11 B10	37.62(14)	B7A B6A B11A B5A	-138.81(16)
B7 B6 B11 B10	-101.31(17)	C1A B6A B11A B7A	100.90(15)
C2 B6 B11 B12	-0.22(18)	C2A B6A B11A B7A	37.86(14)
C1 B6 B11 B12	62.51(17)	B5A B6A B11A B7A	138.81(16)
B5 B6 B11 B12	100.80(16)	C1A B6A B11A B10A	0.1(2)
B7 B6 B11 B12	-38.13(14)	C2A B6A B11A B10A	-62.95(19)
C2 B6 B11 B5	-101.02(14)	B7A B6A B11A B10A	-100.81(18)
C1 B6 B11 B5	-38.29(13)	B5A B6A B11A B10A	38.01(15)
B7 B6 B11 B5	-138.93(15)	C1A B6A B11A B12A	63.10(19)
C2 B6 B11 B7	37.92(12)	C2A B6A B11A B12A	0.0(2)
C1 B6 B11 B7	100.65(14)	B7A B6A B11A B12A	-37.81(16)
B5 B6 B11 B7	138.93(15)	B5A B6A B11A B12A	101.01(18)
B5 B10 B11 B6	-37.60(14)	C1A B5A B11A B6A	38.45(13)
B12 B10 B11 B6	100.81(16)	B4A B5A B11A B6A	100.11(15)
B9 B10 B11 B6	63.04(19)	B10A B5A B11A B6A	137.46(16)
B4 B10 B11 B6	-0.2(2)	C1A B5A B11A B7A	1.4(2)
B5 B10 B11 B12	-138.41(15)	B4A B5A B11A B7A	63.0(2)
B9 B10 B11 B12	-37.77(14)	B10A B5A B11A B7A	100.39(18)
B4 B10 B11 B12	-101.01(17)	B6A B5A B11A B7A	-37.07(15)
B12 B10 B11 B5	138.41(15)	C1A B5A B11A B10A	-99.01(16)
B9 B10 B11 B5	100.64(16)	B4A B5A B11A B10A	-37.36(15)
B4 B10 B11 B5	37.39(14)	B6A B5A B11A B10A	-137.46(16)
B5 B10 B11 B7	-101.22(15)	C1A B5A B11A B12A	-62.08(19)
B12 B10 B11 B7	37.19(14)	B4A B5A B11A B12A	-0.4(2)

B9 B10 B11 B7	-0.58(19)	B10A B5A B11A B12A	36.93(15)
B4 B10 B11 B7	-63.83(19)	B6A B5A B11A B12A	-100.53(17)
C1 B5 B11 B6	38.59(13)	C2A B7A B11A B6A	-38.35(14)
B10 B5 B11 B6	137.64(16)	B8A B7A B11A B6A	-100.21(16)
B4 B5 B11 B6	100.16(15)	B12A B7A B11A B6A	-137.54(16)
C1 B5 B11 B10	-99.05(15)	C2A B7A B11A B5A	-1.2(2)
B6 B5 B11 B10	-137.64(16)	B6A B7A B11A B5A	37.18(15)
B4 B5 B11 B10	-37.48(14)	B8A B7A B11A B5A	-63.0(2)
C1 B5 B11 B12	-61.92(18)	B12A B7A B11A B5A	-100.37(18)
B6 B5 B11 B12	-100.51(16)	C2A B7A B11A B10A	62.45(19)
B10 B5 B11 B12	37.13(14)	B6A B7A B11A B10A	100.79(16)
B4 B5 B11 B12	-0.35(19)	B8A B7A B11A B10A	0.6(2)
C1 B5 B11 B7	1.6(2)	B12A B7A B11A B10A	-36.75(15)
B6 B5 B11 B7	-36.96(14)	C2A B7A B11A B12A	99.19(17)
B10 B5 B11 B7	100.68(17)	B6A B7A B11A B12A	137.54(16)
B4 B5 B11 B7	63.21(19)	B8A B7A B11A B12A	37.33(15)
C2 B7 B11 B6	-38.17(13)	B12A B10A B11A B6A	100.39(17)
B8 B7 B11 B6	-99.81(14)	B4A B10A B11A B6A	-0.8(2)
B12 B7 B11 B6	-137.25(15)	B5A B10A B11A B6A	-38.14(15)
C2 B7 B11 B10	61.95(17)	B9A B10A B11A B6A	62.5(2)
B8 B7 B11 B10	0.31(18)	B12A B10A B11A B5A	138.53(16)
B6 B7 B11 B10	100.12(15)	B4A B10A B11A B5A	37.35(14)
B12 B7 B11 B10	-37.13(14)	B9A B10A B11A B5A	100.65(16)
C2 B7 B11 B12	99.08(15)	B12A B10A B11A B7A	36.85(15)
B8 B7 B11 B12	37.44(13)	B4A B10A B11A B7A	-64.3(2)
B6 B7 B11 B12	137.25(15)	B5A B10A B11A B7A	-101.68(16)
C2 B7 B11 B5	-1.42(19)	B9A B10A B11A B7A	-1.0(2)
B8 B7 B11 B5	-63.06(18)	B4A B10A B11A B12A	-101.18(18)
B6 B7 B11 B5	36.75(14)	B5A B10A B11A B12A	-138.53(16)
B12 B7 B11 B5	-100.50(17)	B9A B10A B11A B12A	-37.88(15)
B5 B10 B12 B9	-100.38(16)	B4A B10A B12A B9A	-36.87(15)
B11 B10 B12 B9	-137.71(15)	B5A B10A B12A B9A	-100.36(17)
B4 B10 B12 B9	-36.87(14)	B11A B10A B12A B9A	-137.47(16)
B5 B10 B12 B11	37.33(14)	B4A B10A B12A B8A	0.1(2)
B9 B10 B12 B11	137.71(15)	B5A B10A B12A B8A	-63.4(2)
B4 B10 B12 B11	100.84(16)	B11A B10A B12A B8A	-100.55(18)
B5 B10 B12 B8	-63.25(19)	B9A B10A B12A B8A	36.92(16)
B9 B10 B12 B8	37.13(14)	B4A B10A B12A B7A	63.7(2)
B11 B10 B12 B8	-100.58(15)	B5A B10A B12A B7A	0.2(2)
B4 B10 B12 B8	0.3(2)	B11A B10A B12A B7A	-36.94(15)
B5 B10 B12 B7	0.1(2)	B9A B10A B12A B7A	100.54(18)
B9 B10 B12 B7	100.44(16)	B4A B10A B12A B11A	100.61(17)
B11 B10 B12 B7	-37.27(14)	B5A B10A B12A B11A	37.11(15)
B4 B10 B12 B7	63.6(2)	B9A B10A B12A B11A	137.47(16)
B3 B9 B12 B10	100.98(16)	B3A B9A B12A B10A	100.79(17)
B4 B9 B12 B10	37.09(14)	B8A B9A B12A B10A	138.83(16)
B8 B9 B12 B10	138.54(15)	B4A B9A B12A B10A	36.81(15)
B3 B9 B12 B11	63.24(18)	B3A B9A B12A B8A	-38.05(15)
B4 B9 B12 B11	-0.66(19)	B4A B9A B12A B8A	-102.02(17)

B10 B9 B12 B11	-37.75(14)	B10A B9A B12A B8A	-138.83(16)
B8 B9 B12 B11	100.79(15)	B3A B9A B12A B7A	-0.5(2)
B3 B9 B12 B8	-37.55(13)	B8A B9A B12A B7A	37.54(15)
B4 B9 B12 B8	-101.45(15)	B4A B9A B12A B7A	-64.5(2)
B10 B9 B12 B8	-138.54(15)	B10A B9A B12A B7A	-101.30(18)
B3 B9 B12 B7	-0.3(2)	B3A B9A B12A B11A	62.8(2)
B4 B9 B12 B7	-64.15(18)	B8A B9A B12A B11A	100.83(18)
B10 B9 B12 B7	-101.24(16)	B4A B9A B12A B11A	-1.2(2)
B8 B9 B12 B7	37.29(14)	B10A B9A B12A B11A	-38.00(16)
B6 B11 B12 B10	-100.24(16)	C2A B8A B12A B10A	62.6(2)
B5 B11 B12 B10	-37.10(14)	B9A B8A B12A B10A	-37.08(16)
B7 B11 B12 B10	-138.47(15)	B3A B8A B12A B10A	0.4(2)
B6 B11 B12 B9	-62.52(18)	B7A B8A B12A B10A	100.88(18)
B10 B11 B12 B9	37.72(14)	C2A B8A B12A B9A	99.69(16)
B5 B11 B12 B9	0.62(19)	B3A B8A B12A B9A	37.50(15)
B7 B11 B12 B9	-100.75(15)	B7A B8A B12A B9A	137.96(16)
B6 B11 B12 B8	0.9(2)	C2A B8A B12A B7A	-38.27(14)
B10 B11 B12 B8	101.09(16)	B9A B8A B12A B7A	-137.96(16)
B5 B11 B12 B8	63.99(18)	B3A B8A B12A B7A	-100.46(16)
B7 B11 B12 B8	-37.37(13)	C2A B8A B12A B11A	-1.0(2)
B6 B11 B12 B7	38.23(14)	B9A B8A B12A B11A	-100.70(18)
B10 B11 B12 B7	138.47(15)	B3A B8A B12A B11A	-63.2(2)
B5 B11 B12 B7	101.37(15)	B7A B8A B12A B11A	37.26(16)
C2 B8 B12 B10	62.33(18)	C2A B7A B12A B10A	-62.9(2)
B7 B8 B12 B10	101.01(16)	B6A B7A B12A B10A	-0.6(2)
B3 B8 B12 B10	0.00(19)	B8A B7A B12A B10A	-101.27(18)
B9 B8 B12 B10	-37.11(14)	B11A B7A B12A B10A	37.01(16)
C2 B8 B12 B9	99.44(14)	C2A B7A B12A B9A	0.8(2)
B7 B8 B12 B9	138.13(15)	B6A B7A B12A B9A	63.2(2)
B3 B8 B12 B9	37.11(13)	B8A B7A B12A B9A	-37.50(15)
C2 B8 B12 B11	-1.16(18)	B11A B7A B12A B9A	100.78(18)
B7 B8 B12 B11	37.53(14)	C2A B7A B12A B8A	38.33(14)
B3 B8 B12 B11	-63.49(18)	B6A B7A B12A B8A	100.68(16)
B9 B8 B12 B11	-100.60(16)	B11A B7A B12A B8A	138.28(16)
C2 B8 B12 B7	-38.68(12)	C2A B7A B12A B11A	-99.96(16)
B3 B8 B12 B7	-101.02(14)	B6A B7A B12A B11A	-37.60(15)
B9 B8 B12 B7	-138.13(15)	B8A B7A B12A B11A	-138.28(16)
C2 B7 B12 B10	-62.24(18)	B6A B11A B12A B10A	-100.84(17)
B8 B7 B12 B10	-100.82(16)	B5A B11A B12A B10A	-37.10(15)
B6 B7 B12 B10	-0.4(2)	B7A B11A B12A B10A	-138.74(16)
B11 B7 B12 B10	37.23(15)	B6A B11A B12A B9A	-62.7(2)
C2 B7 B12 B9	1.19(19)	B5A B11A B12A B9A	1.0(2)
B8 B7 B12 B9	-37.39(14)	B7A B11A B12A B9A	-100.64(18)
B6 B7 B12 B9	63.05(18)	B10A B11A B12A B9A	38.10(16)
B11 B7 B12 B9	100.66(16)	B6A B11A B12A B8A	0.6(2)
C2 B7 B12 B11	-99.47(15)	B5A B11A B12A B8A	64.3(2)
B8 B7 B12 B11	-138.05(15)	B7A B11A B12A B8A	-37.30(16)
B6 B7 B12 B11	-37.60(13)	B10A B11A B12A B8A	101.44(18)
C2 B7 B12 B8	38.58(12)	B6A B11A B12A B7A	37.90(15)

B6 B7 B12 B8	100.44(14)	B5A B11A B12A B7A	101.64(16)
B11 B7 B12 B8	138.05(15)	B10A B11A B12A B7A	138.74(16)
B9 B4 C1 C13	146.84(15)	B11A B5A C1A C13A	-147.01(15)
B5 B4 C1 C13	-111.68(16)	B4A B5A C1A C13A	111.27(16)
B3 B4 C1 C13	108.14(16)	B10A B5A C1A C13A	150.36(15)
B10 B4 C1 C13	-150.59(15)	B6A B5A C1A C13A	-108.48(16)
B9 B4 C1 B5	-101.48(16)	B11A B5A C1A B4A	101.72(16)
B3 B4 C1 B5	-140.19(14)	B10A B5A C1A B4A	39.09(14)
B10 B4 C1 B5	-38.92(14)	B6A B5A C1A B4A	140.25(15)
B9 B4 C1 B3	38.70(13)	B11A B5A C1A B6A	-38.53(14)
B5 B4 C1 B3	140.19(14)	B4A B5A C1A B6A	-140.25(15)
B10 B4 C1 B3	101.27(15)	B10A B5A C1A B6A	-101.16(16)
B9 B4 C1 C2	0.73(18)	B11A B5A C1A B3A	63.81(18)
B5 B4 C1 C2	102.21(13)	B4A B5A C1A B3A	-37.91(14)
B3 B4 C1 C2	-37.98(12)	B10A B5A C1A B3A	1.2(2)
B10 B4 C1 C2	63.29(16)	B6A B5A C1A B3A	102.34(15)
B9 B4 C1 B6	-64.05(17)	B11A B5A C1A C2A	-1.07(18)
B5 B4 C1 B6	37.43(13)	B4A B5A C1A C2A	-102.79(14)
B3 B4 C1 B6	-102.75(14)	B10A B5A C1A C2A	-63.70(17)
B10 B4 C1 B6	-1.48(18)	B6A B5A C1A C2A	37.46(13)
B6 B5 C1 C13	-106.60(16)	B10A B4A C1A C13A	-150.07(15)
B10 B5 C1 C13	152.07(15)	B5A B4A C1A C13A	-110.89(16)
B4 B5 C1 C13	113.03(16)	B9A B4A C1A C13A	147.22(15)
B11 B5 C1 C13	-145.27(15)	B3A B4A C1A C13A	108.69(16)
B6 B5 C1 B4	140.37(14)	B10A B4A C1A B5A	-39.19(14)
B10 B5 C1 B4	39.04(13)	B9A B4A C1A B5A	-101.89(17)
B11 B5 C1 B4	101.70(16)	B3A B4A C1A B5A	-140.43(15)
B6 B5 C1 B3	102.41(14)	B10A B4A C1A B6A	-1.17(19)
B10 B5 C1 B3	1.09(18)	B5A B4A C1A B6A	38.01(14)
B4 B5 C1 B3	-37.96(13)	B9A B4A C1A B6A	-63.88(18)
B11 B5 C1 B3	63.74(18)	B3A B4A C1A B6A	-102.41(15)
B6 B5 C1 C2	37.45(12)	B10A B4A C1A B3A	101.24(16)
B10 B5 C1 C2	-63.88(16)	B5A B4A C1A B3A	140.43(15)
B4 B5 C1 C2	-102.92(13)	B9A B4A C1A B3A	38.53(14)
B11 B5 C1 C2	-1.22(18)	B10A B4A C1A C2A	63.72(17)
B10 B5 C1 B6	-101.33(15)	B5A B4A C1A C2A	102.91(14)
B4 B5 C1 B6	-140.37(14)	B9A B4A C1A C2A	1.01(18)
B11 B5 C1 B6	-38.67(13)	B3A B4A C1A C2A	-37.52(13)
C2 B3 C1 C13	107.96(15)	C2A B6A C1A C13A	-109.74(15)
B9 B3 C1 C13	-152.71(14)	B11A B6A C1A C13A	150.34(15)
B8 B3 C1 C13	144.28(14)	B7A B6A C1A C13A	-146.56(15)
B4 B3 C1 C13	-113.85(16)	B5A B6A C1A C13A	111.55(15)
C2 B3 C1 B4	-138.19(13)	C2A B6A C1A B5A	138.71(14)
B9 B3 C1 B4	-38.86(13)	B11A B6A C1A B5A	38.79(14)
B8 B3 C1 B4	-101.87(15)	B7A B6A C1A B5A	101.89(16)
C2 B3 C1 B5	-100.16(14)	C2A B6A C1A B4A	100.61(14)
B9 B3 C1 B5	-0.83(18)	B11A B6A C1A B4A	0.69(19)
B8 B3 C1 B5	-63.84(17)	B7A B6A C1A B4A	63.79(18)
B4 B3 C1 B5	38.03(14)	B5A B6A C1A B4A	-38.10(14)

B9 B3 C1 C2	99.33(14)	C2A B6A C1A B3A	32.68(13)
B8 B3 C1 C2	36.32(11)	B11A B6A C1A B3A	-67.24(17)
B4 B3 C1 C2	138.19(13)	B7A B6A C1A B3A	-4.14(19)
C2 B3 C1 B6	-33.06(12)	B5A B6A C1A B3A	-106.03(15)
B9 B3 C1 B6	66.27(16)	B11A B6A C1A C2A	-99.92(15)
B8 B3 C1 B6	3.26(18)	B7A B6A C1A C2A	-36.82(13)
B4 B3 C1 B6	105.13(15)	B5A B6A C1A C2A	-138.71(14)
C2 B6 C1 C13	-108.42(14)	C2A B3A C1A C13A	109.62(15)
B11 B6 C1 C13	152.14(14)	B9A B3A C1A C13A	-150.67(15)
B5 B6 C1 C13	113.04(15)	B8A B3A C1A C13A	146.43(15)
B7 B6 C1 C13	-144.54(14)	B4A B3A C1A C13A	-111.79(16)
C2 B6 C1 B4	100.80(13)	C2A B3A C1A B5A	-100.61(14)
B11 B6 C1 B4	1.37(18)	B9A B3A C1A B5A	-0.90(19)
B5 B6 C1 B4	-37.74(14)	B8A B3A C1A B5A	-63.79(18)
B7 B6 C1 B4	64.69(17)	B4A B3A C1A B5A	37.98(14)
C2 B6 C1 B5	138.54(14)	C2A B3A C1A B4A	-138.59(14)
B11 B6 C1 B5	39.11(14)	B9A B3A C1A B4A	-38.88(15)
B7 B6 C1 B5	102.42(15)	B8A B3A C1A B4A	-101.77(17)
C2 B6 C1 B3	33.14(12)	C2A B3A C1A B6A	-32.70(13)
B11 B6 C1 B3	-66.29(17)	B9A B3A C1A B6A	67.01(18)
B5 B6 C1 B3	-105.40(15)	B8A B3A C1A B6A	4.11(19)
B7 B6 C1 B3	-2.97(18)	B4A B3A C1A B6A	105.89(15)
B11 B6 C1 C2	-99.43(14)	B9A B3A C1A C2A	99.71(15)
B5 B6 C1 C2	-138.54(14)	B8A B3A C1A C2A	36.82(13)
B7 B6 C1 C2	-36.12(12)	B4A B3A C1A C2A	138.59(14)
B7 B8 C2 C19	111.55(16)	B6A B7A C2A C19A	107.83(18)
B3 B8 C2 C19	-107.76(16)	B8A B7A C2A C19A	-112.06(18)
B9 B8 C2 C19	-146.56(15)	B12A B7A C2A C19A	-150.91(17)
B12 B8 C2 C19	150.86(15)	B11A B7A C2A C19A	146.21(17)
B3 B8 C2 B7	140.70(14)	B6A B7A C2A B8A	-140.11(16)
B9 B8 C2 B7	101.89(16)	B12A B7A C2A B8A	-38.85(16)
B12 B8 C2 B7	39.31(13)	B11A B7A C2A B8A	-101.73(18)
B7 B8 C2 B6	-38.25(13)	B6A B7A C2A B3A	-102.13(15)
B3 B8 C2 B6	102.45(14)	B8A B7A C2A B3A	37.98(15)
B9 B8 C2 B6	63.65(17)	B12A B7A C2A B3A	-0.9(2)
B12 B8 C2 B6	1.07(17)	B11A B7A C2A B3A	-63.75(19)
B7 B8 C2 C1	-103.04(13)	B8A B7A C2A B6A	140.11(16)
B3 B8 C2 C1	37.66(12)	B12A B7A C2A B6A	101.25(17)
B9 B8 C2 C1	-1.14(17)	B11A B7A C2A B6A	38.37(15)
B12 B8 C2 C1	-63.72(15)	B6A B7A C2A C1A	-37.86(13)
B7 B8 C2 B3	-140.70(14)	B8A B7A C2A C1A	102.25(15)
B9 B8 C2 B3	-38.80(13)	B12A B7A C2A C1A	63.39(18)
B12 B8 C2 B3	-101.38(15)	B11A B7A C2A C1A	0.51(19)
B8 B7 C2 C19	-111.94(16)	B9A B8A C2A C19A	-146.65(16)
B6 B7 C2 C19	108.27(16)	B12A B8A C2A C19A	150.75(17)
B12 B7 C2 C19	-151.10(15)	B3A B8A C2A C19A	-108.20(17)
B11 B7 C2 C19	146.53(15)	B7A B8A C2A C19A	111.88(18)
B6 B7 C2 B8	-139.79(14)	B9A B8A C2A B7A	101.46(18)
B12 B7 C2 B8	-39.16(13)	B12A B8A C2A B7A	38.87(16)

B11 B7 C2 B8	-101.53(15)	B3A B8A C2A B7A	139.92(16)
B8 B7 C2 B6	139.79(14)	B9A B8A C2A B3A	-38.46(15)
B12 B7 C2 B6	100.63(15)	B12A B8A C2A B3A	-101.05(18)
B11 B7 C2 B6	38.26(13)	B7A B8A C2A B3A	-139.92(16)
B8 B7 C2 C1	102.20(13)	B9A B8A C2A B6A	63.68(19)
B6 B7 C2 C1	-37.59(12)	B12A B8A C2A B6A	1.1(2)
B12 B7 C2 C1	63.04(16)	B3A B8A C2A B6A	102.14(15)
B11 B7 C2 C1	0.67(17)	B7A B8A C2A B6A	-37.78(15)
B8 B7 C2 B3	37.40(13)	B9A B8A C2A C1A	-0.56(19)
B6 B7 C2 B3	-102.39(14)	B12A B8A C2A C1A	-63.15(18)
B12 B7 C2 B3	-1.76(18)	B3A B8A C2A C1A	37.90(13)
B11 B7 C2 B3	-64.13(17)	B7A B8A C2A C1A	-102.02(15)
C1 B6 C2 C19	108.84(14)	C1A B3A C2A C19A	-109.34(15)
B11 B6 C2 C19	-151.33(14)	B9A B3A C2A C19A	151.55(15)
B5 B6 C2 C19	145.19(14)	B8A B3A C2A C19A	112.85(17)
B7 B6 C2 C19	-112.54(15)	B4A B3A C2A C19A	-145.42(15)
C1 B6 C2 B8	-100.37(13)	C1A B3A C2A B7A	99.67(14)
B11 B6 C2 B8	-0.54(18)	B9A B3A C2A B7A	0.56(19)
B5 B6 C2 B8	-64.02(16)	B8A B3A C2A B7A	-38.13(15)
B7 B6 C2 B8	38.25(13)	B4A B3A C2A B7A	63.59(17)
C1 B6 C2 B7	-138.63(13)	C1A B3A C2A B8A	137.81(15)
B11 B6 C2 B7	-38.79(13)	B9A B3A C2A B8A	38.70(15)
B5 B6 C2 B7	-102.27(15)	B4A B3A C2A B8A	101.72(16)
B11 B6 C2 C1	99.83(14)	C1A B3A C2A B6A	32.52(13)
B5 B6 C2 C1	36.36(12)	B9A B3A C2A B6A	-66.59(17)
B7 B6 C2 C1	138.63(13)	B8A B3A C2A B6A	-105.29(16)
C1 B6 C2 B3	-32.98(12)	B4A B3A C2A B6A	-3.56(18)
B11 B6 C2 B3	66.85(16)	B9A B3A C2A C1A	-99.11(15)
B5 B6 C2 B3	3.38(17)	B8A B3A C2A C1A	-137.81(15)
B7 B6 C2 B3	105.64(14)	B4A B3A C2A C1A	-36.08(12)
C13 C1 C2 C19	-1.0(2)	C1A B6A C2A C19A	109.44(15)
B4 C1 C2 C19	146.72(14)	B11A B6A C2A C19A	-151.48(15)
B5 C1 C2 C19	-146.43(14)	B7A B6A C2A C19A	-112.79(17)
B3 C1 C2 C19	107.82(16)	B5A B6A C2A C19A	145.44(14)
B6 C1 C2 C19	-108.21(16)	C1A B6A C2A B7A	-137.77(15)
C13 C1 C2 B8	-147.46(14)	B11A B6A C2A B7A	-38.69(15)
B4 C1 C2 B8	0.27(17)	B5A B6A C2A B7A	-101.77(17)
B5 C1 C2 B8	67.12(16)	C1A B6A C2A B8A	-99.80(15)
B3 C1 C2 B8	-38.63(13)	B11A B6A C2A B8A	-0.72(19)
B6 C1 C2 B8	105.33(14)	B7A B6A C2A B8A	37.97(16)
C13 C1 C2 B7	145.77(14)	B5A B6A C2A B8A	-63.80(18)
B4 C1 C2 B7	-66.50(16)	C1A B6A C2A B3A	-32.54(13)
B5 C1 C2 B7	0.35(17)	B11A B6A C2A B3A	66.53(17)
B3 C1 C2 B7	-105.40(14)	B7A B6A C2A B3A	105.22(16)
B6 C1 C2 B7	38.56(13)	B5A B6A C2A B3A	3.45(18)
C13 C1 C2 B6	107.21(15)	B11A B6A C2A C1A	99.08(15)
B4 C1 C2 B6	-105.06(14)	B7A B6A C2A C1A	137.77(15)
B5 C1 C2 B6	-38.21(13)	B5A B6A C2A C1A	36.00(12)
B3 C1 C2 B6	-143.96(14)	C13A C1A C2A C19A	0.1(2)

C13 C1 C2 B3	-108.83(15)	B5A C1A C2A C19A	-146.16(15)
B4 C1 C2 B3	38.90(13)	B4A C1A C2A C19A	146.66(16)
B5 C1 C2 B3	105.75(14)	B6A C1A C2A C19A	-107.64(17)
B6 C1 C2 B3	143.96(14)	B3A C1A C2A C19A	108.04(17)
C1 B3 C2 C19	-108.88(14)	C13A C1A C2A B7A	146.59(15)
B9 B3 C2 C19	151.95(14)	B5A C1A C2A B7A	0.36(18)
B8 B3 C2 C19	112.84(16)	B4A C1A C2A B7A	-66.81(17)
B4 B3 C2 C19	-145.24(14)	B6A C1A C2A B7A	38.88(14)
C1 B3 C2 B8	138.28(13)	B3A C1A C2A B7A	-105.44(16)
B9 B3 C2 B8	39.11(13)	C13A C1A C2A B8A	-146.89(15)
B4 B3 C2 B8	101.92(15)	B5A C1A C2A B8A	66.88(16)
C1 B3 C2 B7	100.78(14)	B4A C1A C2A B8A	-0.30(18)
B9 B3 C2 B7	1.60(18)	B6A C1A C2A B8A	105.40(15)
B8 B3 C2 B7	-37.50(14)	B3A C1A C2A B8A	-38.92(14)
B4 B3 C2 B7	64.42(16)	C13A C1A C2A B3A	-107.97(16)
C1 B3 C2 B6	33.05(12)	B5A C1A C2A B3A	105.80(15)
B9 B3 C2 B6	-66.12(16)	B4A C1A C2A B3A	38.62(13)
B8 B3 C2 B6	-105.23(14)	B6A C1A C2A B3A	144.32(14)
B4 B3 C2 B6	-3.31(17)	C13A C1A C2A B6A	107.71(16)
B9 B3 C2 C1	-99.18(14)	B5A C1A C2A B6A	-38.52(14)
B8 B3 C2 C1	-138.28(13)	B4A C1A C2A B6A	-105.69(15)
B4 B3 C2 C1	-36.36(12)	B3A C1A C2A B6A	-144.32(14)
B4 C1 C13 C14	-61.8(2)	B5A C1A C13A C18A	52.5(2)
B5 C1 C13 C14	-137.24(19)	B4A C1A C13A C18A	126.78(18)
B3 C1 C13 C14	12.1(2)	B6A C1A C13A C18A	-20.5(2)
C2 C1 C13 C14	81.7(2)	B3A C1A C13A C18A	-159.96(16)
B6 C1 C13 C14	150.59(18)	C2A C1A C13A C18A	-90.21(19)
B4 C1 C13 C18	116.6(2)	B5A C1A C13A C14A	-121.75(19)
B5 C1 C13 C18	41.1(2)	B4A C1A C13A C14A	-47.5(2)
B3 C1 C13 C18	-169.60(18)	B6A C1A C13A C14A	165.25(16)
C2 C1 C13 C18	-100.0(2)	B3A C1A C13A C14A	25.8(2)
B6 C1 C13 C18	-31.1(2)	C2A C1A C13A C14A	95.53(19)
C18 C13 C14 C15	1.5(3)	C18A C13A C14A C15A	-0.1(3)
C1 C13 C14 C15	179.9(2)	C1A C13A C14A C15A	174.29(19)
C13 C14 C15 C16	-0.1(4)	C13A C14A C15A C16A	0.4(4)
C14 C15 C16 C17	-1.5(4)	C14A C15A C16A C17A	0.0(4)
C15 C16 C17 C18	1.6(4)	C15A C16A C17A C18A	-0.6(4)
C14 C13 C18 C17	-1.4(3)	C14A C13A C18A C17A	-0.5(3)
C1 C13 C18 C17	-179.8(2)	C1A C13A C18A C17A	-174.89(18)
C16 C17 C18 C13	-0.2(4)	C16A C17A C18A C13A	0.9(3)
B8 C2 C19 C24	43.6(2)	B7A C2A C19A C20A	-51.2(2)
B7 C2 C19 C24	118.35(19)	B8A C2A C19A C20A	-126.2(2)
B6 C2 C19 C24	-168.34(16)	B3A C2A C19A C20A	160.60(17)
C1 C2 C19 C24	-98.70(19)	B6A C2A C19A C20A	21.7(2)
B3 C2 C19 C24	-29.4(2)	C1A C2A C19A C20A	91.1(2)
B8 C2 C19 C20	-133.58(19)	B7A C2A C19A C24A	125.0(2)
B7 C2 C19 C20	-58.8(2)	B8A C2A C19A C24A	49.9(2)
B6 C2 C19 C20	14.5(2)	B3A C2A C19A C24A	-23.3(2)
C1 C2 C19 C20	84.1(2)	B6A C2A C19A C24A	-162.13(18)

B3 C2 C19 C20	153.43(17)	C1A C2A C19A C24A	-92.8(2)
C24 C19 C20 C21	0.7(3)	C24A C19A C20A C21A	0.5(3)
C2 C19 C20 C21	178.0(2)	C2A C19A C20A C21A	176.75(19)
C19 C20 C21 C22	-0.1(4)	C19A C20A C21A C22A	0.3(4)
C20 C21 C22 C23	-0.7(4)	C20A C21A C22A C23A	-0.8(4)
C21 C22 C23 C24	0.9(4)	C21A C22A C23A C24A	0.4(4)
C20 C19 C24 C23	-0.5(3)	C20A C19A C24A C23A	-0.9(3)
C2 C19 C24 C23	-177.79(19)	C2A C19A C24A C23A	-177.2(2)
C22 C23 C24 C19	-0.3(4)	C22A C23A C24A C19A	0.5(4)

Table S4. Bond lengths (Å) of **2**.

Cr1 C26	1.8537(16)	B6 B7	1.774(2)
Cr1 C27	1.8566(17)	B7 C2	1.707(2)
Cr1 C25	1.8578(18)	B7 B11	1.773(2)
Cr1 C18	2.1963(15)	B7 B12	1.774(2)
Cr1 C14	2.1970(15)	B7 B8	1.778(2)
Cr1 C16	2.2046(15)	B8 C2	1.711(2)
Cr1 C15	2.2162(15)	B8 B12	1.772(2)
Cr1 C17	2.2184(15)	B8 B9	1.775(3)
Cr1 C13	2.2252(13)	B9 B10	1.784(3)
O1 C25	1.143(2)	B9 B12	1.786(3)
O2 C26	1.144(2)	B10 B12	1.776(3)
O3 C27	1.143(2)	B10 B11	1.777(2)
B3 C2	1.724(2)	B11 B12	1.778(2)
B3 C1	1.7320(19)	C1 C13	1.4994(18)
B3 B9	1.763(2)	C1 C2	1.7404(18)
B3 B4	1.779(2)	C2 C19	1.5004(19)
B3 B8	1.781(2)	C13 C14	1.4185(18)
B4 C1	1.7095(19)	C13 C18	1.4192(18)
B4 B5	1.776(2)	C14 C15	1.408(2)
B4 B10	1.777(2)	C15 C16	1.406(2)
B4 B9	1.779(2)	C16 C17	1.400(2)
B5 C1	1.711(2)	C17 C18	1.405(2)
B5 B11	1.776(2)	C19 C24	1.386(2)
B5 B6	1.777(2)	C19 C20	1.393(2)
B5 B10	1.780(2)	C20 C21	1.388(3)
B6 C1	1.7205(18)	C21 C22	1.368(4)
B6 C2	1.7282(19)	C22 C23	1.373(4)
B6 B11	1.764(2)	C23 C24	1.386(3)

Table S5. Bond angles (º) of **2**.

C26 Cr1 C27	86.73(7)	B3 B9 B10	107.78(11)
C26 Cr1 C25	88.02(8)	B8 B9 B10	107.85(12)
C27 Cr1 C25	88.96(8)	B4 B9 B10	59.85(10)
C26 Cr1 C18	134.38(7)	B3 B9 B12	107.65(11)
C27 Cr1 C18	87.41(6)	B8 B9 B12	59.69(10)
C25 Cr1 C18	137.05(7)	B4 B9 B12	107.75(12)
C26 Cr1 C14	136.72(6)	B10 B9 B12	59.67(10)
C27 Cr1 C14	136.06(6)	B12 B10 B11	60.05(10)
C25 Cr1 C14	86.97(7)	B12 B10 B4	108.28(12)
C18 Cr1 C14	67.17(5)	B11 B10 B4	107.68(11)
C26 Cr1 C16	86.92(7)	B12 B10 B5	108.28(12)
C27 Cr1 C16	135.89(7)	B11 B10 B5	59.89(9)
C25 Cr1 C16	134.34(8)	B4 B10 B5	59.91(9)
C18 Cr1 C16	67.03(6)	B12 B10 B9	60.23(10)
C14 Cr1 C16	67.02(6)	B11 B10 B9	107.86(12)
C26 Cr1 C15	102.48(6)	B4 B10 B9	59.95(10)
C27 Cr1 C15	166.63(6)	B5 B10 B9	107.96(11)
C25 Cr1 C15	100.90(7)	B6 B11 B7	60.19(9)
C18 Cr1 C15	79.22(6)	B6 B11 B5	60.27(9)
C14 Cr1 C15	37.20(5)	B7 B11 B5	108.89(11)
C16 Cr1 C15	37.08(6)	B6 B11 B10	107.88(11)
C26 Cr1 C17	101.01(7)	B7 B11 B10	108.13(12)
C27 Cr1 C17	102.43(7)	B5 B11 B10	60.13(9)
C25 Cr1 C17	165.76(7)	B6 B11 B12	107.82(11)
C18 Cr1 C17	37.12(5)	B7 B11 B12	59.94(10)
C14 Cr1 C17	78.90(5)	B5 B11 B12	108.39(11)
C16 Cr1 C17	36.91(6)	B10 B11 B12	59.94(10)
C15 Cr1 C17	66.57(6)	B8 B12 B7	60.18(9)
C26 Cr1 C13	166.49(6)	B8 B12 B10	108.30(12)
C27 Cr1 C13	101.87(6)	B7 B12 B10	108.16(11)
C25 Cr1 C13	102.40(7)	B8 B12 B11	108.05(11)
C18 Cr1 C13	37.44(5)	B7 B12 B11	59.90(9)
C14 Cr1 C13	37.41(5)	B10 B12 B11	60.02(10)
C16 Cr1 C13	79.64(5)	B8 B12 B9	59.83(10)
C15 Cr1 C13	67.38(5)	B7 B12 B9	107.79(11)
C17 Cr1 C13	67.16(5)	B10 B12 B9	60.10(10)
C2 B3 C1	60.47(7)	B11 B12 B9	107.73(12)
C2 B3 B9	105.78(11)	C13 C1 B4	122.72(10)
C1 B3 B9	105.80(11)	C13 C1 B5	124.39(10)
C2 B3 B4	106.98(10)	B4 C1 B5	62.56(9)
C1 B3 B4	58.26(8)	C13 C1 B6	118.74(10)
B9 B3 B4	60.31(10)	B4 C1 B6	112.92(10)
C2 B3 B8	58.42(8)	B5 C1 B6	62.38(8)
C1 B3 B8	107.06(10)	C13 C1 B3	116.11(10)
B9 B3 B8	60.10(10)	B4 C1 B3	62.24(9)
B4 B3 B8	108.56(11)	B5 C1 B3	112.96(10)
C1 B4 B5	58.77(8)	B6 C1 B3	110.71(10)

C1 B4 B10	105.69(11)	C13 C1 C2	116.58(10)
B5 B4 B10	60.12(9)	B4 C1 C2	109.40(10)
C1 B4 B3	59.50(8)	B5 C1 C2	109.77(10)
B5 B4 B3	107.70(11)	B6 C1 C2	59.91(8)
B10 B4 B3	107.35(12)	B3 C1 C2	59.54(8)
C1 B4 B9	106.06(11)	C19 C2 B7	121.66(11)
B5 B4 B9	108.31(11)	C19 C2 B8	120.55(11)
B10 B4 B9	60.20(10)	B7 C2 B8	62.68(9)
B3 B4 B9	59.40(9)	C19 C2 B3	117.64(11)
C1 B5 B11	105.72(11)	B7 C2 B3	113.11(10)
C1 B5 B4	58.67(8)	B8 C2 B3	62.45(9)
B11 B5 B4	107.80(11)	C19 C2 B6	119.38(10)
C1 B5 B6	59.06(8)	B7 C2 B6	62.17(9)
B11 B5 B6	59.55(9)	B8 C2 B6	112.94(10)
B4 B5 B6	107.12(11)	B3 C2 B6	110.73(10)
C1 B5 B10	105.49(11)	C19 C2 C1	119.60(10)
B11 B5 B10	59.98(9)	B7 C2 C1	109.42(10)
B4 B5 B10	59.97(9)	B8 C2 C1	109.87(10)
B6 B5 B10	107.19(11)	B3 C2 C1	59.99(8)
C1 B6 C2	60.62(7)	B6 C2 C1	59.47(7)
C1 B6 B11	105.82(10)	C14 C13 C18	117.83(12)
C2 B6 B11	105.61(10)	C14 C13 C1	120.21(11)
C1 B6 B7	107.27(10)	C18 C13 C1	121.96(11)
C2 B6 B7	58.32(8)	C14 C13 Cr1	70.21(8)
B11 B6 B7	60.15(9)	C18 C13 Cr1	70.18(8)
C1 B6 B5	58.56(8)	C1 C13 Cr1	130.99(9)
C2 B6 B5	107.30(10)	C15 C14 C13	121.30(13)
B11 B6 B5	60.17(9)	C15 C14 Cr1	72.14(8)
B7 B6 B5	108.78(10)	C13 C14 Cr1	72.37(7)
C2 B7 B11	106.16(11)	C16 C15 C14	119.48(13)
C2 B7 B6	59.50(8)	C16 C15 Cr1	71.01(9)
B11 B7 B6	59.67(9)	C14 C15 Cr1	70.66(8)
C2 B7 B12	105.66(11)	C17 C16 C15	120.30(13)
B11 B7 B12	60.16(10)	C17 C16 Cr1	72.08(9)
B6 B7 B12	107.59(11)	C15 C16 Cr1	71.91(9)
C2 B7 B8	58.77(8)	C16 C17 C18	120.03(13)
B11 B7 B8	108.02(12)	C16 C17 Cr1	71.01(9)
B6 B7 B8	107.66(10)	C18 C17 Cr1	70.59(8)
B12 B7 B8	59.86(10)	C17 C18 C13	120.97(13)
C2 B8 B12	105.56(11)	C17 C18 Cr1	72.29(8)
C2 B8 B9	105.84(11)	C13 C18 Cr1	72.39(7)
B12 B8 B9	60.48(10)	C24 C19 C20	118.78(15)
C2 B8 B7	58.55(8)	C24 C19 C2	120.42(14)
B12 B8 B7	59.96(9)	C20 C19 C2	120.63(13)
B9 B8 B7	108.13(12)	C21 C20 C19	120.42(19)
C2 B8 B3	59.14(8)	C22 C21 C20	120.0(2)
B12 B8 B3	107.50(12)	C21 C22 C23	120.10(18)
B9 B8 B3	59.46(10)	C22 C23 C24	120.6(2)
B7 B8 B3	107.13(10)	C23 C24 C19	120.1(2)

B3 B9 B8	60.44(9)	O1 C25 Cr1	179.6(2)
B3 B9 B4	60.29(9)	O2 C26 Cr1	178.97(15)
B8 B9 B4	108.82(11)	O3 C27 Cr1	178.73(15)

Table S6. Torsional angles (º) of **2**.

C2 B3 B4 C1	37.36(9)	B11 B5 C1 B4	101.56(12)
B9 B3 B4 C1	136.17(11)	B6 B5 C1 B4	140.07(11)
B8 B3 B4 C1	98.99(11)	B10 B5 C1 B4	39.04(10)
C2 B3 B4 B5	2.41(14)	B11 B5 C1 B6	-38.50(10)
C1 B3 B4 B5	-34.95(10)	B4 B5 C1 B6	-140.07(11)
B9 B3 B4 B5	101.22(12)	B10 B5 C1 B6	-101.03(11)
B8 B3 B4 B5	64.04(13)	B11 B5 C1 B3	63.73(13)
C2 B3 B4 B10	-60.97(13)	B4 B5 C1 B3	-37.84(11)
C1 B3 B4 B10	-98.32(11)	B6 B5 C1 B3	102.23(11)
B9 B3 B4 B10	37.84(11)	B10 B5 C1 B3	1.20(14)
B8 B3 B4 B10	0.67(14)	B11 B5 C1 C2	-0.63(13)
C2 B3 B4 B9	-98.81(12)	B4 B5 C1 C2	-102.19(11)
C1 B3 B4 B9	-136.17(11)	B6 B5 C1 C2	37.88(9)
B8 B3 B4 B9	-37.18(10)	B10 B5 C1 C2	-63.15(12)
B10 B4 B5 C1	135.49(11)	C2 B6 C1 C13	-105.79(12)
B3 B4 B5 C1	35.26(10)	B11 B6 C1 C13	154.92(11)
B9 B4 B5 C1	98.06(12)	B7 B6 C1 C13	-142.06(11)
C1 B4 B5 B11	-97.92(11)	B5 B6 C1 C13	116.10(12)
B10 B4 B5 B11	37.58(11)	C2 B6 C1 B4	99.90(11)
B3 B4 B5 B11	-62.66(13)	B11 B6 C1 B4	0.60(14)
B9 B4 B5 B11	0.14(15)	B7 B6 C1 B4	63.62(13)
C1 B4 B5 B6	-35.17(9)	B5 B6 C1 B4	-38.21(11)
B10 B4 B5 B6	100.32(12)	C2 B6 C1 B5	138.11(10)
B3 B4 B5 B6	0.08(14)	B11 B6 C1 B5	38.81(10)
B9 B4 B5 B6	62.88(13)	B7 B6 C1 B5	101.83(11)
C1 B4 B5 B10	-135.49(11)	C2 B6 C1 B3	32.27(10)
B3 B4 B5 B10	-100.23(12)	B11 B6 C1 B3	-67.02(13)
B9 B4 B5 B10	-37.44(11)	B7 B6 C1 B3	-4.00(14)
B11 B5 B6 C1	135.96(11)	B5 B6 C1 B3	-105.83(11)
B4 B5 B6 C1	35.01(9)	B11 B6 C1 C2	-99.29(11)
B10 B5 B6 C1	98.08(11)	B7 B6 C1 C2	-36.28(9)
C1 B5 B6 C2	-37.55(9)	B5 B6 C1 C2	-138.11(10)
B11 B5 B6 C2	98.42(11)	C2 B3 C1 C13	106.85(11)
B4 B5 B6 C2	-2.54(13)	B9 B3 C1 C13	-153.68(11)
B10 B5 B6 C2	60.53(13)	B4 B3 C1 C13	-114.98(12)
C1 B5 B6 B11	-135.96(11)	B8 B3 C1 C13	143.39(11)
B4 B5 B6 B11	-100.96(12)	C2 B3 C1 B4	-138.17(11)
B10 B5 B6 B11	-37.88(11)	B9 B3 C1 B4	-38.70(11)
C1 B5 B6 B7	-99.19(11)	B8 B3 C1 B4	-101.63(12)
B11 B5 B6 B7	36.78(10)	C2 B3 C1 B5	-100.20(11)
B4 B5 B6 B7	-64.18(13)	B9 B3 C1 B5	-0.73(14)
B10 B5 B6 B7	-1.10(14)	B4 B3 C1 B5	37.97(11)
C1 B6 B7 C2	37.29(9)	B8 B3 C1 B5	-63.66(14)
B11 B6 B7 C2	135.96(11)	C2 B3 C1 B6	-32.41(10)
B5 B6 B7 C2	99.17(11)	B9 B3 C1 B6	67.05(13)
C1 B6 B7 B11	-98.67(11)	B4 B3 C1 B6	105.75(11)
C2 B6 B7 B11	-135.96(11)	B8 B3 C1 B6	4.12(14)

B5 B6 B7 B11	-36.79(10)	B9 B3 C1 C2	99.47(11)
C1 B6 B7 B12	-60.87(13)	B4 B3 C1 C2	138.17(11)
C2 B6 B7 B12	-98.16(12)	B8 B3 C1 C2	36.54(10)
B11 B6 B7 B12	37.80(11)	B11 B7 C2 C19	147.70(12)
B5 B6 B7 B12	1.01(14)	B6 B7 C2 C19	109.04(13)
C1 B6 B7 B8	2.27(14)	B12 B7 C2 C19	-149.47(12)
C2 B6 B7 B8	-35.02(10)	B8 B7 C2 C19	-110.71(13)
B11 B6 B7 B8	100.94(12)	B11 B7 C2 B8	-101.59(12)
B5 B6 B7 B8	64.15(13)	B6 B7 C2 B8	-140.25(11)
B11 B7 B8 C2	98.34(11)	B12 B7 C2 B8	-38.76(11)
B6 B7 B8 C2	35.33(10)	B11 B7 C2 B3	-63.45(13)
B12 B7 B8 C2	135.80(12)	B6 B7 C2 B3	-102.11(11)
C2 B7 B8 B12	-135.80(12)	B12 B7 C2 B3	-0.63(15)
B11 B7 B8 B12	-37.46(11)	B8 B7 C2 B3	38.14(11)
B6 B7 B8 B12	-100.48(12)	B11 B7 C2 B6	38.66(10)
C2 B7 B8 B9	-97.84(12)	B12 B7 C2 B6	101.48(12)
B11 B7 B8 B9	0.51(14)	B8 B7 C2 B6	140.25(11)
B6 B7 B8 B9	-62.51(14)	B11 B7 C2 C1	1.33(13)
B12 B7 B8 B9	37.97(11)	B6 B7 C2 C1	-37.33(9)
C2 B7 B8 B3	-35.13(10)	B12 B7 C2 C1	64.15(13)
B11 B7 B8 B3	63.21(14)	B8 B7 C2 C1	102.92(11)
B6 B7 B8 B3	0.20(15)	B12 B8 C2 C19	151.19(12)
B12 B7 B8 B3	100.67(13)	B9 B8 C2 C19	-145.73(12)
C1 B3 B8 C2	-37.45(9)	B7 B8 C2 C19	112.40(13)
B9 B3 B8 C2	-136.20(12)	B3 B8 C2 C19	-107.44(13)
B4 B3 B8 C2	-98.93(11)	B12 B8 C2 B7	38.79(11)
C2 B3 B8 B12	98.01(12)	B9 B8 C2 B7	101.87(12)
C1 B3 B8 B12	60.55(14)	B3 B8 C2 B7	140.16(11)
B9 B3 B8 B12	-38.19(11)	B12 B8 C2 B3	-101.37(13)
B4 B3 B8 B12	-0.93(14)	B9 B8 C2 B3	-38.29(11)
C2 B3 B8 B9	136.20(12)	B7 B8 C2 B3	-140.16(11)
C1 B3 B8 B9	98.75(12)	B12 B8 C2 B6	0.90(15)
B4 B3 B8 B9	37.27(11)	B9 B8 C2 B6	63.98(14)
C2 B3 B8 B7	34.88(10)	B7 B8 C2 B6	-37.89(11)
C1 B3 B8 B7	-2.57(15)	B3 B8 C2 B6	102.27(11)
B9 B3 B8 B7	-101.32(13)	B12 B8 C2 C1	-63.41(13)
B4 B3 B8 B7	-64.05(14)	B9 B8 C2 C1	-0.33(14)
C2 B3 B9 B8	-37.79(10)	B7 B8 C2 C1	-102.20(11)
C1 B3 B9 B8	-100.90(11)	B3 B8 C2 C1	37.96(10)
B4 B3 B9 B8	-138.64(11)	C1 B3 C2 C19	-109.96(12)
C2 B3 B9 B4	100.85(11)	B9 B3 C2 C19	150.53(11)
C1 B3 B9 B4	37.74(10)	B4 B3 C2 C19	-146.33(11)
B8 B3 B9 B4	138.64(11)	B8 B3 C2 C19	111.96(13)
C2 B3 B9 B10	63.06(14)	C1 B3 C2 B7	99.85(11)
C1 B3 B9 B10	-0.05(15)	B9 B3 C2 B7	0.34(14)
B4 B3 B9 B10	-37.79(11)	B4 B3 C2 B7	63.48(13)
B8 B3 B9 B10	100.84(13)	B8 B3 C2 B7	-38.23(11)
C2 B3 B9 B12	0.09(15)	C1 B3 C2 B8	138.08(11)
C1 B3 B9 B12	-63.02(14)	B9 B3 C2 B8	38.57(11)

B4 B3 B9 B12	-100.76(13)	B4 B3 C2 B8	101.71(12)
B8 B3 B9 B12	37.88(11)	C1 B3 C2 B6	32.25(9)
C2 B8 B9 B3	38.14(10)	B9 B3 C2 B6	-67.25(13)
B12 B8 B9 B3	137.34(11)	B4 B3 C2 B6	-4.12(14)
B7 B8 B9 B3	99.60(11)	B8 B3 C2 B6	-105.83(11)
C2 B8 B9 B4	0.82(15)	B9 B3 C2 C1	-99.51(12)
B12 B8 B9 B4	100.01(13)	B4 B3 C2 C1	-36.37(9)
B7 B8 B9 B4	62.27(14)	B8 B3 C2 C1	-138.08(11)
B3 B8 B9 B4	-37.33(11)	C1 B6 C2 C19	109.03(12)
C2 B8 B9 B10	-62.59(14)	B11 B6 C2 C19	-151.33(11)
B12 B8 B9 B10	36.61(11)	B7 B6 C2 C19	-112.57(13)
B7 B8 B9 B10	-1.13(15)	B5 B6 C2 C19	145.66(11)
B3 B8 B9 B10	-100.73(12)	C1 B6 C2 B7	-138.40(10)
C2 B8 B9 B12	-99.20(12)	B11 B6 C2 B7	-38.76(10)
B7 B8 B9 B12	-37.74(11)	B5 B6 C2 B7	-101.77(11)
B3 B8 B9 B12	-137.34(11)	C1 B6 C2 B8	-100.30(11)
C1 B4 B9 B3	-38.39(10)	B11 B6 C2 B8	-0.66(14)
B5 B4 B9 B3	-100.16(12)	B7 B6 C2 B8	38.10(11)
B10 B4 B9 B3	-137.56(12)	B5 B6 C2 B8	-63.67(13)
C1 B4 B9 B8	-0.99(15)	C1 B6 C2 B3	-32.45(10)
B5 B4 B9 B8	-62.76(15)	B11 B6 C2 B3	67.20(12)
B10 B4 B9 B8	-100.16(13)	B7 B6 C2 B3	105.95(11)
B3 B4 B9 B8	37.39(11)	B5 B6 C2 B3	4.19(13)
C1 B4 B9 B10	99.17(12)	B11 B6 C2 C1	99.64(11)
B5 B4 B9 B10	37.40(11)	B7 B6 C2 C1	138.40(10)
B3 B4 B9 B10	137.56(12)	B5 B6 C2 C1	36.63(9)
C1 B4 B9 B12	62.21(14)	C13 C1 C2 C19	0.69(15)
B5 B4 B9 B12	0.45(15)	B4 C1 C2 C19	145.48(12)
B10 B4 B9 B12	-36.96(11)	B5 C1 C2 C19	-147.62(11)
B3 B4 B9 B12	100.60(13)	B6 C1 C2 C19	-108.67(12)
C1 B4 B10 B12	-62.47(14)	B3 C1 C2 C19	106.75(13)
B5 B4 B10 B12	-100.98(13)	C13 C1 C2 B7	147.87(11)
B3 B4 B10 B12	-0.15(15)	B4 C1 C2 B7	-67.34(12)
B9 B4 B10 B12	37.33(11)	B5 C1 C2 B7	-0.45(13)
C1 B4 B10 B11	1.00(15)	B6 C1 C2 B7	38.51(10)
B5 B4 B10 B11	-37.51(11)	B3 C1 C2 B7	-106.08(11)
B3 B4 B10 B11	63.32(14)	C13 C1 C2 B8	-145.09(11)
B9 B4 B10 B11	100.81(13)	B4 C1 C2 B8	-0.30(13)
C1 B4 B10 B5	38.51(10)	B5 C1 C2 B8	66.59(12)
B3 B4 B10 B5	100.83(11)	B6 C1 C2 B8	105.55(11)
B9 B4 B10 B5	138.31(12)	B3 C1 C2 B8	-39.04(10)
C1 B4 B10 B9	-99.81(12)	C13 C1 C2 B3	-106.05(12)
B5 B4 B10 B9	-138.31(12)	B4 C1 C2 B3	38.74(10)
B3 B4 B10 B9	-37.49(10)	B5 C1 C2 B3	105.63(11)
C1 B5 B10 B12	62.56(14)	B6 C1 C2 B3	144.59(11)
B11 B5 B10 B12	-36.92(11)	C13 C1 C2 B6	109.36(11)
B4 B5 B10 B12	100.97(13)	B4 C1 C2 B6	-105.85(11)
B6 B5 B10 B12	0.77(15)	B5 C1 C2 B6	-38.96(10)
C1 B5 B10 B11	99.47(11)	B3 C1 C2 B6	-144.59(11)

B4 B5 B10 B11	137.89(12)	B4 C1 C13 C14	-37.41(17)
B6 B5 B10 B11	37.69(10)	B5 C1 C13 C14	-114.46(14)
C1 B5 B10 B4	-38.41(10)	B6 C1 C13 C14	170.92(11)
B11 B5 B10 B4	-137.89(12)	B3 C1 C13 C14	35.04(16)
B6 B5 B10 B4	-100.20(11)	C2 C1 C13 C14	102.33(13)
C1 B5 B10 B9	-1.17(15)	B4 C1 C13 C18	142.57(13)
B11 B5 B10 B9	-100.65(13)	B5 C1 C13 C18	65.52(17)
B4 B5 B10 B9	37.24(11)	B6 C1 C13 C18	-9.10(17)
B6 B5 B10 B9	-62.96(14)	B3 C1 C13 C18	-144.98(12)
B3 B9 B10 B12	-100.45(13)	C2 C1 C13 C18	-77.69(15)
B8 B9 B10 B12	-36.62(11)	B4 C1 C13 Cr1	51.85(16)
B4 B9 B10 B12	-138.44(11)	B5 C1 C13 Cr1	-25.21(17)
B3 B9 B10 B11	-62.51(15)	B6 C1 C13 Cr1	-99.82(13)
B8 B9 B10 B11	1.32(15)	B3 C1 C13 Cr1	124.30(11)
B4 B9 B10 B11	-100.50(12)	C2 C1 C13 Cr1	-168.42(8)
B12 B9 B10 B11	37.94(11)	C26 Cr1 C13 C14	-71.8(3)
B3 B9 B10 B4	37.99(11)	C27 Cr1 C13 C14	159.44(9)
B8 B9 B10 B4	101.82(12)	C25 Cr1 C13 C14	67.89(10)
B12 B9 B10 B4	138.44(11)	C18 Cr1 C13 C14	-131.08(12)
B3 B9 B10 B5	0.77(16)	C16 Cr1 C13 C14	-65.53(8)
B8 B9 B10 B5	64.60(14)	C15 Cr1 C13 C14	-28.90(8)
B4 B9 B10 B5	-37.22(11)	C17 Cr1 C13 C14	-101.92(9)
B12 B9 B10 B5	101.22(12)	C26 Cr1 C13 C18	59.2(3)
C1 B6 B11 B7	101.13(11)	C27 Cr1 C13 C18	-69.48(10)
C2 B6 B11 B7	37.90(10)	C25 Cr1 C13 C18	-161.03(10)
B5 B6 B11 B7	139.19(11)	C14 Cr1 C13 C18	131.08(12)
C1 B6 B11 B5	-38.05(10)	C16 Cr1 C13 C18	65.55(9)
C2 B6 B11 B5	-101.29(11)	C15 Cr1 C13 C18	102.18(9)
B7 B6 B11 B5	-139.19(11)	C17 Cr1 C13 C18	29.15(8)
C1 B6 B11 B10	0.07(14)	C26 Cr1 C13 C1	174.8(2)
C2 B6 B11 B10	-63.17(13)	C27 Cr1 C13 C1	46.13(12)
B7 B6 B11 B10	-101.07(12)	C25 Cr1 C13 C1	-45.43(13)
B5 B6 B11 B10	38.12(11)	C18 Cr1 C13 C1	115.61(14)
C1 B6 B11 B12	63.37(13)	C14 Cr1 C13 C1	-113.31(14)
C2 B6 B11 B12	0.14(14)	C16 Cr1 C13 C1	-178.84(12)
B7 B6 B11 B12	-37.76(11)	C15 Cr1 C13 C1	-142.21(12)
B5 B6 B11 B12	101.43(12)	C17 Cr1 C13 C1	144.76(13)
C2 B7 B11 B6	-38.58(9)	C18 C13 C14 C15	1.95(19)
B12 B7 B11 B6	-137.66(11)	C1 C13 C14 C15	-178.07(12)
B8 B7 B11 B6	-100.33(11)	Cr1 C13 C14 C15	55.26(12)
C2 B7 B11 B5	-1.72(14)	C18 C13 C14 Cr1	-53.31(11)
B6 B7 B11 B5	36.86(10)	C1 C13 C14 Cr1	126.67(11)
B12 B7 B11 B5	-100.79(12)	C26 Cr1 C14 C15	28.64(13)
B8 B7 B11 B5	-63.47(13)	C27 Cr1 C14 C15	-162.15(10)
C2 B7 B11 B10	62.06(13)	C25 Cr1 C14 C15	112.51(10)
B6 B7 B11 B10	100.64(12)	C18 Cr1 C14 C15	-102.65(9)
B12 B7 B11 B10	-37.01(11)	C16 Cr1 C14 C15	-28.99(9)
B8 B7 B11 B10	0.31(14)	C17 Cr1 C14 C15	-65.69(9)
C2 B7 B11 B12	99.07(12)	C13 Cr1 C14 C15	-132.46(12)

B6 B7 B11 B12	137.66(11)	C26 Cr1 C14 C13	161.10(10)
B8 B7 B11 B12	37.33(10)	C27 Cr1 C14 C13	-29.69(13)
C1 B5 B11 B6	38.27(9)	C25 Cr1 C14 C13	-115.03(10)
B4 B5 B11 B6	99.78(11)	C18 Cr1 C14 C13	29.81(8)
B10 B5 B11 B6	137.35(11)	C16 Cr1 C14 C13	103.47(9)
C1 B5 B11 B7	1.45(14)	C15 Cr1 C14 C13	132.46(12)
B4 B5 B11 B7	62.95(14)	C17 Cr1 C14 C13	66.77(8)
B6 B5 B11 B7	-36.82(10)	C13 C14 C15 C16	-1.9(2)
B10 B5 B11 B7	100.53(12)	Cr1 C14 C15 C16	53.52(12)
C1 B5 B11 B10	-99.08(12)	C13 C14 C15 Cr1	-55.37(12)
B4 B5 B11 B10	-37.57(11)	C26 Cr1 C15 C16	67.41(10)
B6 B5 B11 B10	-137.35(11)	C27 Cr1 C15 C16	-65.3(3)
C1 B5 B11 B12	-62.19(13)	C25 Cr1 C15 C16	157.79(10)
B4 B5 B11 B12	-0.68(15)	C18 Cr1 C15 C16	-65.98(9)
B6 B5 B11 B12	-100.46(12)	C14 Cr1 C15 C16	-132.25(13)
B10 B5 B11 B12	36.89(11)	C17 Cr1 C15 C16	-29.32(8)
B12 B10 B11 B6	100.65(12)	C13 Cr1 C15 C16	-103.20(9)
B4 B10 B11 B6	-0.67(15)	C26 Cr1 C15 C14	-160.33(9)
B5 B10 B11 B6	-38.19(10)	C27 Cr1 C15 C14	66.9(3)
B9 B10 B11 B6	62.63(14)	C25 Cr1 C15 C14	-69.96(10)
B12 B10 B11 B7	37.02(11)	C18 Cr1 C15 C14	66.27(8)
B4 B10 B11 B7	-64.30(14)	C16 Cr1 C15 C14	132.25(13)
B5 B10 B11 B7	-101.82(11)	C17 Cr1 C15 C14	102.93(9)
B9 B10 B11 B7	-1.01(15)	C13 Cr1 C15 C14	29.05(8)
B12 B10 B11 B5	138.84(12)	C14 C15 C16 C17	2.2(2)
B4 B10 B11 B5	37.52(11)	Cr1 C15 C16 C17	55.54(13)
B9 B10 B11 B5	100.81(12)	C14 C15 C16 Cr1	-53.35(12)
B4 B10 B11 B12	-101.32(13)	C26 Cr1 C16 C17	112.96(10)
B5 B10 B11 B12	-138.84(12)	C27 Cr1 C16 C17	30.86(13)
B9 B10 B11 B12	-38.02(11)	C25 Cr1 C16 C17	-162.84(11)
C2 B8 B12 B7	-38.12(10)	C18 Cr1 C16 C17	-28.63(8)
B9 B8 B12 B7	-137.78(12)	C14 Cr1 C16 C17	-102.49(9)
B3 B8 B12 B7	-100.05(11)	C15 Cr1 C16 C17	-131.57(13)
C2 B8 B12 B10	62.76(15)	C13 Cr1 C16 C17	-65.57(9)
B9 B8 B12 B10	-36.91(11)	C26 Cr1 C16 C15	-115.47(10)
B7 B8 B12 B10	100.88(13)	C27 Cr1 C16 C15	162.43(10)
B3 B8 B12 B10	0.83(15)	C25 Cr1 C16 C15	-31.27(13)
C2 B8 B12 B11	-0.77(15)	C18 Cr1 C16 C15	102.94(9)
B9 B8 B12 B11	-100.43(13)	C14 Cr1 C16 C15	29.08(8)
B7 B8 B12 B11	37.35(11)	C17 Cr1 C16 C15	131.57(13)
B3 B8 B12 B11	-62.70(14)	C13 Cr1 C16 C15	66.00(9)
C2 B8 B12 B9	99.66(12)	C15 C16 C17 C18	-2.7(2)
B7 B8 B12 B9	137.78(12)	Cr1 C16 C17 C18	52.78(13)
B3 B8 B12 B9	37.73(10)	C15 C16 C17 Cr1	-55.46(13)
C2 B7 B12 B8	38.25(10)	C26 Cr1 C17 C16	-69.51(10)
B11 B7 B12 B8	138.18(12)	C27 Cr1 C17 C16	-158.56(9)
B6 B7 B12 B8	100.60(12)	C25 Cr1 C17 C16	59.0(3)
C2 B7 B12 B10	-62.88(14)	C18 Cr1 C17 C16	133.03(13)
B11 B7 B12 B10	37.06(11)	C14 Cr1 C17 C16	66.35(9)

B6 B7 B12 B10	-0.52(15)	C15 Cr1 C17 C16	29.45(9)
B8 B7 B12 B10	-101.13(13)	C13 Cr1 C17 C16	103.64(10)
C2 B7 B12 B11	-99.93(11)	C26 Cr1 C17 C18	157.46(9)
B6 B7 B12 B11	-37.58(10)	C27 Cr1 C17 C18	68.41(10)
B8 B7 B12 B11	-138.18(12)	C25 Cr1 C17 C18	-74.0(3)
C2 B7 B12 B9	0.65(15)	C14 Cr1 C17 C18	-66.67(9)
B11 B7 B12 B9	100.58(13)	C16 Cr1 C17 C18	-133.03(13)
B6 B7 B12 B9	63.00(15)	C15 Cr1 C17 C18	-103.58(10)
B8 B7 B12 B9	-37.60(11)	C13 Cr1 C17 C18	-29.39(8)
B11 B10 B12 B8	-100.72(12)	C16 C17 C18 C13	2.8(2)
B4 B10 B12 B8	-0.42(16)	Cr1 C17 C18 C13	55.80(12)
B5 B10 B12 B8	-63.88(15)	C16 C17 C18 Cr1	-52.98(13)
B9 B10 B12 B8	36.79(11)	C14 C13 C18 C17	-2.4(2)
B11 B10 B12 B7	-37.00(11)	C1 C13 C18 C17	177.59(13)
B4 B10 B12 B7	63.30(15)	Cr1 C13 C18 C17	-55.76(12)
B5 B10 B12 B7	-0.16(16)	C14 C13 C18 Cr1	53.33(11)
B9 B10 B12 B7	100.51(13)	C1 C13 C18 Cr1	-126.65(11)
B4 B10 B12 B11	100.30(12)	C26 Cr1 C18 C17	-31.76(13)
B5 B10 B12 B11	36.85(11)	C27 Cr1 C18 C17	-114.63(10)
B9 B10 B12 B11	137.51(11)	C25 Cr1 C18 C17	159.69(11)
B11 B10 B12 B9	-137.51(11)	C14 Cr1 C18 C17	102.13(10)
B4 B10 B12 B9	-37.21(11)	C16 Cr1 C18 C17	28.48(9)
B5 B10 B12 B9	-100.67(12)	C15 Cr1 C18 C17	65.22(9)
B6 B11 B12 B8	0.39(16)	C13 Cr1 C18 C17	131.92(13)
B7 B11 B12 B8	-37.48(11)	C26 Cr1 C18 C13	-163.68(9)
B5 B11 B12 B8	64.16(15)	C27 Cr1 C18 C13	113.45(9)
B10 B11 B12 B8	101.14(13)	C25 Cr1 C18 C13	27.77(14)
B6 B11 B12 B7	37.87(10)	C14 Cr1 C18 C13	-29.79(8)
B5 B11 B12 B7	101.64(12)	C16 Cr1 C18 C13	-103.44(9)
B10 B11 B12 B7	138.62(12)	C15 Cr1 C18 C13	-66.70(9)
B6 B11 B12 B10	-100.75(12)	C17 Cr1 C18 C13	-131.92(13)
B7 B11 B12 B10	-138.62(12)	B7 C2 C19 C24	118.35(15)
B5 B11 B12 B10	-36.98(11)	B8 C2 C19 C24	43.54(19)
B6 B11 B12 B9	-62.82(14)	B3 C2 C19 C24	-29.17(18)
B7 B11 B12 B9	-100.69(12)	B6 C2 C19 C24	-168.05(13)
B5 B11 B12 B9	0.95(15)	C1 C2 C19 C24	-98.57(15)
B10 B11 B12 B9	37.93(11)	B7 C2 C19 C20	-57.01(18)
B3 B9 B12 B8	-38.22(11)	B8 C2 C19 C20	-131.81(15)
B4 B9 B12 B8	-101.84(12)	B3 C2 C19 C20	155.48(14)
B10 B9 B12 B8	-138.87(12)	B6 C2 C19 C20	16.60(19)
B3 B9 B12 B7	-0.46(16)	C1 C2 C19 C20	86.07(16)
B8 B9 B12 B7	37.75(11)	C24 C19 C20 C21	0.9(3)
B4 B9 B12 B7	-64.09(15)	C2 C19 C20 C21	176.30(17)
B10 B9 B12 B7	-101.12(13)	C19 C20 C21 C22	-0.1(3)
B3 B9 B12 B10	100.66(13)	C20 C21 C22 C23	-0.6(3)
B8 B9 B12 B10	138.87(12)	C21 C22 C23 C24	0.5(3)
B4 B9 B12 B10	37.04(11)	C22 C23 C24 C19	0.3(3)
B3 B9 B12 B11	62.77(15)	C20 C19 C24 C23	-1.0(2)
B8 B9 B12 B11	100.98(12)	C2 C19 C24 C23	-176.44(15)

B4 B9 B12 B11	-0.86(16)	C26 Cr1 C25 O1	74(37)
B10 B9 B12 B11	-37.89(11)	C27 Cr1 C25 O1	161(100)
B5 B4 C1 C13	-115.01(13)	C18 Cr1 C25 O1	-114(37)
B10 B4 C1 C13	-154.16(12)	C14 Cr1 C25 O1	-63(37)
B3 B4 C1 C13	104.65(13)	C16 Cr1 C25 O1	-10(37)
B9 B4 C1 C13	142.99(12)	C15 Cr1 C25 O1	-28(37)
B10 B4 C1 B5	-39.15(11)	C17 Cr1 C25 O1	-56(37)
B3 B4 C1 B5	-140.34(11)	C13 Cr1 C25 O1	-97(37)
B9 B4 C1 B5	-102.00(12)	C27 Cr1 C26 O2	12(10)
B5 B4 C1 B6	38.14(10)	C25 Cr1 C26 O2	101(10)
B10 B4 C1 B6	-1.01(14)	C18 Cr1 C26 O2	-71(10)
B3 B4 C1 B6	-102.20(11)	C14 Cr1 C26 O2	-175(100)
B9 B4 C1 B6	-63.86(13)	C16 Cr1 C26 O2	-124(10)
B5 B4 C1 B3	140.34(11)	C15 Cr1 C26 O2	-158(10)
B10 B4 C1 B3	101.19(12)	C17 Cr1 C26 O2	-90(10)
B9 B4 C1 B3	38.34(10)	C13 Cr1 C26 O2	-118(10)
B5 B4 C1 C2	102.78(11)	C26 Cr1 C27 O3	-28(8)
B10 B4 C1 C2	63.64(13)	C25 Cr1 C27 O3	-116(8)
B3 B4 C1 C2	-37.55(9)	C18 Cr1 C27 O3	106(8)
B9 B4 C1 C2	0.79(13)	C14 Cr1 C27 O3	159(8)
B11 B5 C1 C13	-145.94(11)	C16 Cr1 C27 O3	54(8)
B4 B5 C1 C13	112.50(13)	C15 Cr1 C27 O3	106(8)
B6 B5 C1 C13	-107.43(13)	C17 Cr1 C27 O3	72(8)
B10 B5 C1 C13	151.54(12)	C13 Cr1 C27 O3	141(8)

Table S7. Bond lengths (Å) of **3**.

Cr1 C25	1.858(4)	C21A Cr7	2.214(5)
Cr1 C26	1.837(4)	C22A C23A	1.386(9)
Cr1 C27	1.858(5)	C22A Cr7	2.180(5)
Cr1 C16	2.199(4)	C23A C24A	1.438(7)
Cr1 C14	2.201(3)	C23A Cr7	2.202(4)
Cr1 C13	2.212(3)	C24A Cr7	2.185(4)
Cr1 C18	2.216(3)	C28A Cr7	1.851(4)
Cr1 C15	2.224(4)	C29A Cr7	1.866(5)
Cr1 C17	2.220(4)	C30A Cr7	1.858(6)
Cr2 C28	1.852(6)	Cr5 C30B	1.827(5)
Cr2 C29	1.852(4)	Cr5 C28B	1.851(4)
Cr2 C30	1.850(4)	Cr5 C29B	1.855(4)
Cr2 C24	2.187(4)	Cr5 C24B	2.195(4)
Cr2 C20	2.204(3)	Cr5 C20B	2.196(4)
Cr2 C21	2.216(4)	Cr5 C23B	2.201(4)
Cr2 C23	2.209(4)	Cr5 C22B	2.206(4)
Cr2 C22	2.208(4)	Cr5 C21B	2.204(5)
Cr2 C19	2.221(3)	Cr5 C19B	2.223(3)
O1 C25	1.133(6)	Cr6 C26B	1.830(6)
O2 C26	1.154(5)	Cr6 C25B	1.847(6)
O3 C27	1.124(6)	Cr6 C27B	1.866(7)
O4 C28	1.143(7)	Cr6 C14B	2.170(6)
O5 C29	1.140(5)	Cr6 C15B	2.190(6)
O6 C30	1.134(5)	Cr6 C18B	2.197(5)
B3 C2	1.726(5)	Cr6 C16B	2.203(7)
B3 C1	1.731(5)	Cr6 C17B	2.205(6)
B3 B8	1.778(5)	Cr6 C13B	2.222(4)
B3 B9	1.761(5)	O1B C25B	1.159(7)
B3 B4	1.792(6)	O2B C26B	1.160(8)
B4 C1	1.703(5)	O3B C27B	1.165(9)
B4 B5	1.783(6)	O4B C28B	1.126(6)
B4 B10	1.775(5)	O5B C29B	1.143(6)
B4 B9	1.780(6)	O6B C30B	1.137(6)
B5 C1	1.727(4)	B3B C1B	1.736(5)
B5 B10	1.767(6)	B3B C2B	1.737(5)
B5 B6	1.768(5)	B3B B9B	1.772(6)
B5 B11	1.780(6)	B3B B4B	1.791(6)
B6 C1	1.715(4)	B3B B8B	1.799(8)
B6 C2	1.727(4)	B4B C1B	1.712(5)
B6 B11	1.764(5)	B4B B9B	1.787(8)
B6 B7	1.783(5)	B4B B10B	1.778(8)
B7 C2	1.704(4)	B4B B5B	1.780(7)
B7 B11	1.772(6)	B5B C1B	1.712(6)
B7 B8	1.778(6)	B5B B11B	1.761(8)
B7 B12	1.766(6)	B5B B6B	1.784(6)
B8 C2	1.708(5)	B5B B10B	1.776(6)
B8 B9	1.775(6)	B6B C2B	1.711(5)

B8 B12	1.790(5)	B6B C1B	1.732(6)
B9 B12	1.795(6)	B6B B7B	1.767(6)
B9 B10	1.788(7)	B6B B11B	1.768(6)
B10 B12	1.765(6)	B7B C2B	1.694(6)
B10 B11	1.776(6)	B7B B11B	1.782(7)
B11 B12	1.787(7)	B7B B12B	1.764(7)
C1 C13	1.505(4)	B7B B8B	1.758(7)
C1 C2	1.724(4)	B8B C2B	1.699(5)
C2 C19	1.509(4)	B8B B12B	1.790(7)
C13 C14	1.390(5)	B8B B9B	1.802(7)
C13 C18	1.432(4)	B9B B10B	1.785(7)
C14 C15	1.427(5)	B9B B12B	1.791(9)
C15 C16	1.384(6)	B10B B12B	1.778(8)
C16 C17	1.383(6)	B10B B11B	1.791(8)
C17 C18	1.421(5)	B11B B12B	1.773(8)
C19 C20	1.414(4)	C1B C13B	1.497(6)
C19 C24	1.411(5)	C1B C2B	1.749(5)
C20 C21	1.405(6)	C2B C19B	1.512(4)
C21 C22	1.409(8)	C13B C14B	1.403(7)
C22 C23	1.386(7)	C13B C18B	1.409(8)
C23 C24	1.403(6)	C14B C15B	1.425(9)
Cr1A C27A	1.818(5)	C15B C16B	1.313(19)
Cr1A C25A	1.826(5)	C16B C17B	1.42(2)
Cr1A C26A	1.853(4)	C17B C18B	1.395(10)
Cr1A C14A	2.178(3)	C19B C24B	1.391(5)
Cr1A C18A	2.176(3)	C19B C20B	1.426(5)
Cr1A C17A	2.197(4)	C20B C21B	1.394(6)
Cr1A C16A	2.199(4)	C21B C22B	1.410(8)
Cr1A C15A	2.216(4)	C22B C23B	1.389(8)
Cr1A C13A	2.219(3)	C23B C24B	1.422(6)
Cr2A C25C	1.845(5)	Cr8 C30C	1.826(5)
Cr2A C27C	1.831(5)	Cr8 C29C	1.843(4)
Cr2A C26C	1.846(4)	Cr8 C28C	1.862(5)
Cr2A C14C	2.193(4)	Cr8 C24C	2.202(4)
Cr2A C15C	2.204(4)	Cr8 C20C	2.210(4)
Cr2A C18C	2.204(3)	Cr8 C22C	2.206(4)
Cr2A C16C	2.208(4)	Cr8 C23C	2.223(5)
Cr2A C17C	2.211(3)	Cr8 C19C	2.225(4)
Cr2A C13C	2.233(3)	Cr8 C21C	2.227(5)
O1A C25A	1.152(6)	O1C C25C	1.126(6)
O2A C26A	1.134(5)	O2C C26C	1.155(5)
O3A C27A	1.159(6)	O3C C27C	1.170(7)
O4A C28A	1.137(5)	O4C C28C	1.118(6)
O5A C29A	1.114(6)	O5C C29C	1.153(5)
O6A C30A	1.148(7)	O6C C30C	1.161(7)
B3A C1A	1.724(5)	B3C C2C	1.737(5)
B3A C2A	1.745(5)	B3C C1C	1.731(5)
B3A B9A	1.758(6)	B3C B8C	1.777(6)
B3A B8A	1.777(6)	B3C B9C	1.756(6)

B3A B4A	1.769(6)	B3C B4C	1.766(6)
B4A C1A	1.696(6)	B4C C1C	1.702(5)
B4A B10A	1.776(7)	B4C B9C	1.773(7)
B4A B5A	1.775(7)	B4C B10C	1.773(6)
B4A B9A	1.778(6)	B4C B5C	1.772(6)
B5A C1A	1.700(5)	B5C C1C	1.690(4)
B5A B6A	1.771(7)	B5C B11C	1.749(6)
B5A B10A	1.771(7)	B5C B10C	1.755(7)
B5A B11A	1.767(6)	B5C B6C	1.784(6)
B6A C2A	1.716(5)	B6C C2C	1.709(5)
B6A C1A	1.734(5)	B6C C1C	1.747(5)
B6A B7A	1.788(5)	B6C B11C	1.771(6)
B6A B11A	1.770(7)	B6C B7C	1.764(6)
B7A C2A	1.708(5)	B7C C2C	1.709(5)
B7A B11A	1.763(7)	B7C B12C	1.803(7)
B7A B12A	1.759(8)	B7C B8C	1.791(6)
B7A B8A	1.753(7)	B7C B11C	1.797(6)
B8A C2A	1.692(5)	B8C C2C	1.713(5)
B8A B9A	1.779(7)	B8C B12C	1.773(6)
B8A B12A	1.773(7)	B8C B9C	1.777(6)
B9A B10A	1.782(8)	B9C B12C	1.773(7)
B9A B12A	1.788(8)	B9C B10C	1.804(7)
B10A B12A	1.789(7)	B10C B11C	1.737(8)
B10A B11A	1.791(9)	B10C B12C	1.769(7)
B11A B12A	1.772(8)	B11C B12C	1.791(7)
C1A C13A	1.505(4)	C1C C13C	1.493(4)
C1A C2A	1.744(4)	C1C C2C	1.737(5)
C2A C19A	1.495(5)	C2C C19C	1.501(5)
C13A C14A	1.405(5)	C13C C14C	1.423(5)
C13A C18A	1.421(5)	C13C C18C	1.431(4)
C14A C15A	1.424(5)	C14C C15C	1.420(5)
C15A C16A	1.388(7)	C15C C16C	1.365(6)
C16A C17A	1.387(6)	C16C C17C	1.420(6)
C17A C18A	1.396(5)	C17C C18C	1.404(5)
C19A C24A	1.392(6)	C19C C24C	1.431(5)
C19A C20A	1.429(6)	C19C C20C	1.423(5)
C19A Cr7	2.229(3)	C20C C21C	1.429(6)
C20A C21A	1.403(7)	C21C C22C	1.364(8)
C20A Cr7	2.201(4)	C22C C23C	1.398(8)
C21A C22A	1.366(9)	C23C C24C	1.429(6)

Table S8. Bond angles (º) of **3**.

C25 Cr1 C26	88.55(19)	O6A C30A Cr7	178.5(6)
C25 Cr1 C27	88.2(2)	C30B Cr5 C28B	87.0(3)
C26 Cr1 C27	87.3(2)	C30B Cr5 C29B	89.9(2)
C25 Cr1 C16	129.9(2)	C28B Cr5 C29B	86.4(2)
C26 Cr1 C16	86.35(16)	C30B Cr5 C24B	86.74(18)
C27 Cr1 C16	141.2(2)	C28B Cr5 C24B	128.61(17)
C25 Cr1 C14	87.16(17)	C29B Cr5 C24B	144.43(17)
C26 Cr1 C14	140.41(17)	C30B Cr5 C20B	145.2(2)
C27 Cr1 C14	131.77(17)	C28B Cr5 C20B	91.79(19)
C16 Cr1 C14	67.03(13)	C29B Cr5 C20B	124.86(19)
C25 Cr1 C13	105.22(16)	C24B Cr5 C20B	67.10(15)
C26 Cr1 C13	164.57(14)	C30B Cr5 C23B	94.2(2)
C27 Cr1 C13	99.80(16)	C28B Cr5 C23B	165.99(18)
C16 Cr1 C13	79.50(12)	C29B Cr5 C23B	107.50(19)
C14 Cr1 C13	36.73(12)	C24B Cr5 C23B	37.74(16)
C25 Cr1 C18	141.20(17)	C20B Cr5 C23B	79.30(17)
C26 Cr1 C18	129.89(15)	C30B Cr5 C22B	124.6(3)
C27 Cr1 C18	88.58(18)	C28B Cr5 C22B	147.5(2)
C16 Cr1 C18	67.03(14)	C29B Cr5 C22B	86.3(2)
C14 Cr1 C18	66.91(14)	C24B Cr5 C22B	67.10(17)
C13 Cr1 C18	37.75(11)	C20B Cr5 C22B	67.00(17)
C25 Cr1 C15	98.4(2)	C23B Cr5 C22B	36.7(2)
C26 Cr1 C15	104.68(17)	C30B Cr5 C21B	160.9(2)
C27 Cr1 C15	166.34(18)	C28B Cr5 C21B	111.9(2)
C16 Cr1 C15	36.45(16)	C29B Cr5 C21B	94.08(19)
C14 Cr1 C15	37.61(12)	C24B Cr5 C21B	79.20(18)
C13 Cr1 C15	66.98(12)	C20B Cr5 C21B	36.93(15)
C18 Cr1 C15	78.78(15)	C23B Cr5 C21B	66.8(2)
C25 Cr1 C17	163.73(19)	C22B Cr5 C21B	37.3(2)
C26 Cr1 C17	97.56(15)	C30B Cr5 C19B	108.29(18)
C27 Cr1 C17	107.1(2)	C28B Cr5 C19B	99.34(16)
C16 Cr1 C17	36.46(17)	C29B Cr5 C19B	161.12(18)
C14 Cr1 C17	78.51(14)	C24B Cr5 C19B	36.69(12)
C13 Cr1 C17	67.34(12)	C20B Cr5 C19B	37.64(13)
C18 Cr1 C17	37.38(12)	C23B Cr5 C19B	67.04(14)
C15 Cr1 C17	65.50(17)	C22B Cr5 C19B	79.13(15)
C28 Cr2 C29	88.8(2)	C21B Cr5 C19B	67.07(15)
C28 Cr2 C30	86.4(3)	C26B Cr6 C25B	87.9(3)
C29 Cr2 C30	88.00(19)	C26B Cr6 C27B	90.2(4)
C28 Cr2 C24	143.0(2)	C25B Cr6 C27B	88.8(3)
C29 Cr2 C24	127.72(18)	C26B Cr6 C14B	132.5(3)
C30 Cr2 C24	89.05(18)	C25B Cr6 C14B	87.4(3)
C28 Cr2 C20	88.2(2)	C27B Cr6 C14B	136.9(3)
C29 Cr2 C20	142.29(16)	C26B Cr6 C15B	97.9(4)
C30 Cr2 C20	129.26(16)	C25B Cr6 C15B	103.1(4)
C24 Cr2 C20	66.84(15)	C27B Cr6 C15B	165.7(3)
C28 Cr2 C21	97.6(3)	C14B Cr6 C15B	38.2(3)

C29 Cr2 C21	106.47(17)	C26B Cr6 C18B	135.0(3)
C30 Cr2 C21	164.99(17)	C25B Cr6 C18B	137.0(2)
C24 Cr2 C21	79.03(16)	C27B Cr6 C18B	88.1(3)
C20 Cr2 C21	37.07(15)	C14B Cr6 C18B	66.8(3)
C28 Cr2 C23	164.3(2)	C15B Cr6 C18B	77.8(3)
C29 Cr2 C23	95.95(18)	C26B Cr6 C16B	84.5(3)
C30 Cr2 C23	108.6(2)	C25B Cr6 C16B	134.5(5)
C24 Cr2 C23	37.21(15)	C27B Cr6 C16B	135.9(6)
C20 Cr2 C23	78.88(17)	C14B Cr6 C16B	66.4(4)
C21 Cr2 C23	66.71(19)	C15B Cr6 C16B	34.8(5)
C28 Cr2 C22	129.4(3)	C18B Cr6 C16B	66.6(4)
C29 Cr2 C22	86.88(17)	C26B Cr6 C17B	100.2(3)
C30 Cr2 C22	143.7(2)	C25B Cr6 C17B	166.7(4)
C24 Cr2 C22	66.46(16)	C27B Cr6 C17B	101.7(5)
C20 Cr2 C22	66.66(16)	C14B Cr6 C17B	79.3(4)
C21 Cr2 C22	37.1(2)	C15B Cr6 C17B	65.4(5)
C23 Cr2 C22	36.58(19)	C18B Cr6 C17B	37.0(3)
C28 Cr2 C19	107.2(2)	C16B Cr6 C17B	37.5(5)
C29 Cr2 C19	163.24(17)	C26B Cr6 C13B	163.3(3)
C30 Cr2 C19	97.75(15)	C25B Cr6 C13B	102.6(2)
C24 Cr2 C19	37.32(14)	C27B Cr6 C13B	102.8(2)
C20 Cr2 C19	37.26(12)	C14B Cr6 C13B	37.23(18)
C21 Cr2 C19	67.24(13)	C15B Cr6 C13B	67.2(2)
C23 Cr2 C19	67.30(14)	C18B Cr6 C13B	37.18(19)
C22 Cr2 C19	79.15(13)	C16B Cr6 C13B	78.9(2)
C2 B3 C1	59.85(18)	C17B Cr6 C13B	67.2(2)
C2 B3 B8	58.3(2)	C1B B3B C2B	60.49(19)
C1 B3 B8	106.6(2)	C1B B3B B9B	105.4(3)
C2 B3 B9	105.2(2)	C2B B3B B9B	105.0(3)
C1 B3 B9	105.1(3)	C1B B3B B4B	58.1(2)
B8 B3 B9	60.2(2)	C2B B3B B4B	106.6(3)
C2 B3 B4	105.9(3)	B9B B3B B4B	60.2(3)
C1 B3 B4	57.8(2)	C1B B3B B8B	106.5(3)
B8 B3 B4	108.3(3)	C2B B3B B8B	57.4(2)
B9 B3 B4	60.1(2)	B9B B3B B8B	60.6(3)
C1 B4 B5	59.3(2)	B4B B3B B8B	108.7(3)
C1 B4 B10	105.1(3)	C1B B4B B9B	105.8(3)
B5 B4 B10	59.6(2)	C1B B4B B10B	105.8(3)
C1 B4 B9	105.5(3)	B9B B4B B10B	60.1(3)
B5 B4 B9	108.4(3)	C1B B4B B5B	58.7(2)
B10 B4 B9	60.4(2)	B9B B4B B5B	107.5(4)
C1 B4 B3	59.3(2)	B10B B4B B5B	59.9(3)
B5 B4 B3	108.2(3)	C1B B4B B3B	59.4(2)
B10 B4 B3	107.1(3)	B9B B4B B3B	59.4(3)
B9 B4 B3	59.1(2)	B10B B4B B3B	107.5(4)
C1 B5 B10	104.4(3)	B5B B4B B3B	107.2(3)
C1 B5 B4	58.0(2)	C1B B5B B11B	106.7(3)
B10 B5 B4	60.0(2)	C1B B5B B4B	58.7(2)
C1 B5 B6	58.75(18)	B11B B5B B4B	108.9(3)

B10 B5 B6	106.8(3)	C1B B5B B6B	59.4(2)
B4 B5 B6	106.6(3)	B11B B5B B6B	59.9(3)
C1 B5 B11	105.4(3)	B4B B5B B6B	107.5(3)
B10 B5 B11	60.1(2)	C1B B5B B10B	105.9(3)
B4 B5 B11	108.1(3)	B11B B5B B10B	60.8(3)
B6 B5 B11	59.6(2)	B4B B5B B10B	60.0(3)
C1 B6 C2	60.14(17)	B6B B5B B10B	108.0(3)
C1 B6 B11	106.6(3)	C2B B6B C1B	61.1(2)
C2 B6 B11	105.8(3)	C2B B6B B7B	58.3(2)
C1 B6 B5	59.41(19)	C1B B6B B7B	107.4(3)
C2 B6 B5	107.8(3)	C2B B6B B11B	106.0(3)
B11 B6 B5	60.5(2)	C1B B6B B11B	105.5(3)
C1 B6 B7	106.6(2)	B7B B6B B11B	60.6(3)
C2 B6 B7	58.07(18)	C2B B6B B5B	107.2(3)
B11 B6 B7	60.0(2)	C1B B6B B5B	58.2(2)
B5 B6 B7	108.7(3)	B7B B6B B5B	108.2(3)
C2 B7 B11	106.4(2)	B11B B6B B5B	59.4(3)
C2 B7 B6	59.32(17)	C2B B7B B11B	106.2(3)
B11 B7 B6	59.5(2)	C2B B7B B12B	106.2(4)
C2 B7 B8	58.70(19)	B11B B7B B12B	60.0(3)
B11 B7 B8	109.3(3)	C2B B7B B6B	59.2(2)
B6 B7 B8	107.7(2)	B11B B7B B6B	59.8(3)
C2 B7 B12	106.1(3)	B12B B7B B6B	107.7(3)
B11 B7 B12	60.7(3)	C2B B7B B8B	58.9(2)
B6 B7 B12	107.6(3)	B11B B7B B8B	109.2(4)
B8 B7 B12	60.7(2)	B12B B7B B8B	61.1(3)
C2 B8 B9	105.4(3)	B6B B7B B8B	108.2(3)
C2 B8 B3	59.32(19)	C2B B8B B7B	58.7(2)
B9 B8 B3	59.4(2)	C2B B8B B12B	104.9(3)
C2 B8 B7	58.5(2)	B7B B8B B12B	59.6(3)
B9 B8 B7	107.6(3)	C2B B8B B3B	59.5(2)
B3 B8 B7	107.4(3)	B7B B8B B3B	107.4(3)
C2 B8 B12	104.9(3)	B12B B8B B3B	106.6(3)
B9 B8 B12	60.5(2)	C2B B8B B9B	105.4(3)
B3 B8 B12	107.7(3)	B7B B8B B9B	107.6(3)
B7 B8 B12	59.3(2)	B12B B8B B9B	59.8(3)
B8 B9 B3	60.3(2)	B3B B8B B9B	59.0(3)
B8 B9 B12	60.2(2)	B10B B9B B3B	108.0(3)
B3 B9 B12	108.1(3)	B10B B9B B4B	59.7(3)
B8 B9 B4	109.0(3)	B3B B9B B4B	60.4(3)
B3 B9 B4	60.8(2)	B10B B9B B8B	108.0(4)
B12 B9 B4	107.5(3)	B3B B9B B8B	60.4(3)
B8 B9 B10	107.5(3)	B4B B9B B8B	108.8(3)
B3 B9 B10	107.9(3)	B10B B9B B12B	59.6(3)
B12 B9 B10	59.0(2)	B3B B9B B12B	107.7(3)
B4 B9 B10	59.7(2)	B4B B9B B12B	107.4(3)
B12 B10 B5	109.4(3)	B8B B9B B12B	59.7(3)
B12 B10 B11	60.6(3)	B9B B10B B12B	60.4(3)
B5 B10 B11	60.3(2)	B9B B10B B4B	60.2(3)

B12 B10 B4	109.1(3)	B12B B10B B4B	108.4(3)
B5 B10 B4	60.5(2)	B9B B10B B5B	107.8(3)
B11 B10 B4	108.7(3)	B12B B10B B5B	107.1(3)
B12 B10 B9	60.7(2)	B4B B10B B5B	60.1(3)
B5 B10 B9	108.7(3)	B9B B10B B11B	107.7(3)
B11 B10 B9	108.8(3)	B12B B10B B11B	59.6(3)
B4 B10 B9	59.9(2)	B4B B10B B11B	107.6(3)
B6 B11 B7	60.5(2)	B5B B10B B11B	59.2(3)
B6 B11 B10	106.6(3)	B12B B11B B7B	59.5(3)
B7 B11 B10	106.8(3)	B12B B11B B5B	108.0(4)
B6 B11 B5	59.9(2)	B7B B11B B5B	108.5(3)
B7 B11 B5	108.6(2)	B12B B11B B6B	107.2(3)
B10 B11 B5	59.6(2)	B7B B11B B6B	59.7(2)
B6 B11 B12	107.5(3)	B5B B11B B6B	60.7(3)
B7 B11 B12	59.5(2)	B12B B11B B10B	59.9(3)
B10 B11 B12	59.4(3)	B7B B11B B10B	107.6(4)
B5 B11 B12	107.9(3)	B5B B11B B10B	60.0(3)
B10 B12 B9	60.3(2)	B6B B11B B10B	108.0(4)
B10 B12 B7	107.5(3)	B11B B12B B7B	60.5(3)
B9 B12 B7	107.2(2)	B11B B12B B10B	60.6(3)
B10 B12 B8	107.8(3)	B7B B12B B10B	109.0(3)
B9 B12 B8	59.4(2)	B11B B12B B8B	108.2(3)
B7 B12 B8	60.0(2)	B7B B12B B8B	59.3(3)
B10 B12 B11	60.0(3)	B10B B12B B8B	108.9(4)
B9 B12 B11	108.1(3)	B11B B12B B9B	108.2(4)
B7 B12 B11	59.8(2)	B7B B12B B9B	107.7(3)
B8 B12 B11	108.1(3)	B10B B12B B9B	60.0(3)
C13 C1 B4	121.8(2)	B8B B12B B9B	60.4(3)
C13 C1 B6	119.7(3)	C13B C1B B5B	124.2(3)
B4 C1 B6	112.9(2)	C13B C1B B4B	124.4(3)
C13 C1 B5	125.1(3)	B5B C1B B4B	62.6(3)
B4 C1 B5	62.7(2)	C13B C1B B3B	117.5(3)
B6 C1 B5	61.8(2)	B5B C1B B3B	112.9(3)
C13 C1 C2	116.2(2)	B4B C1B B3B	62.6(2)
B4 C1 C2	110.0(2)	C13B C1B B6B	116.7(3)
B6 C1 C2	60.27(17)	B5B C1B B6B	62.4(2)
B5 C1 C2	109.8(2)	B4B C1B B6B	113.1(3)
C13 C1 B3	114.2(2)	B3B C1B B6B	110.3(3)
B4 C1 B3	62.9(2)	C13B C1B C2B	116.1(3)
B6 C1 B3	111.6(2)	B5B C1B C2B	108.7(3)
B5 C1 B3	113.8(2)	B4B C1B C2B	109.6(3)
C2 C1 B3	59.93(18)	B3B C1B C2B	59.8(2)
C19 C2 B7	122.1(2)	B6B C1B C2B	58.9(2)
C19 C2 B8	124.7(3)	C19B C2B B7B	126.0(3)
B7 C2 B8	62.8(2)	C19B C2B B6B	119.4(3)
C19 C2 B3	119.1(2)	B7B C2B B6B	62.5(2)
B7 C2 B3	113.3(2)	C19B C2B B8B	121.7(3)
B8 C2 B3	62.3(2)	B7B C2B B8B	62.4(3)
C19 C2 C1	116.3(2)	B6B C2B B8B	113.7(3)

B7 C2 C1	109.7(2)	C19B C2B B3B	114.0(3)
B8 C2 C1	110.1(2)	B7B C2B B3B	113.3(3)
B3 C2 C1	60.22(19)	B6B C2B B3B	111.3(3)
C19 C2 B6	115.0(3)	B8B C2B B3B	63.1(3)
B7 C2 B6	62.6(2)	C19B C2B C1B	115.4(3)
B8 C2 B6	113.6(2)	B7B C2B C1B	109.9(3)
B3 C2 B6	111.3(2)	B6B C2B C1B	60.1(2)
C1 C2 B6	59.58(18)	B8B C2B C1B	110.5(3)
C14 C13 C18	119.2(3)	B3B C2B C1B	59.7(2)
C14 C13 C1	122.0(3)	C14B C13B C18B	117.5(5)
C18 C13 C1	118.7(3)	C14B C13B C1B	120.8(5)
C14 C13 Cr1	71.18(18)	C18B C13B C1B	121.7(4)
C18 C13 Cr1	71.26(18)	C14B C13B Cr6	69.4(3)
C1 C13 Cr1	131.6(2)	C18B C13B Cr6	70.4(3)
C13 C14 C15	120.7(3)	C1B C13B Cr6	133.5(3)
C13 C14 Cr1	72.09(18)	C13B C14B C15B	119.5(8)
C15 C14 Cr1	72.1(2)	C13B C14B Cr6	73.4(3)
C16 C15 C14	119.6(4)	C15B C14B Cr6	71.7(4)
C16 C15 Cr1	70.8(2)	C16B C15B C14B	121.8(9)
C14 C15 Cr1	70.3(2)	C16B C15B Cr6	73.1(6)
C15 C16 C17	120.7(3)	C14B C15B Cr6	70.2(3)
C15 C16 Cr1	72.8(2)	C15B C16B C17B	120.9(7)
C17 C16 Cr1	72.6(2)	C15B C16B Cr6	72.1(4)
C16 C17 C18	120.7(3)	C17B C16B Cr6	71.3(5)
C16 C17 Cr1	70.9(2)	C18B C17B C16B	118.4(9)
C18 C17 Cr1	71.1(2)	C18B C17B Cr6	71.2(3)
C17 C18 C13	118.9(3)	C16B C17B Cr6	71.2(4)
C17 C18 Cr1	71.5(2)	C17B C18B C13B	121.8(8)
C13 C18 Cr1	70.99(18)	C17B C18B Cr6	71.8(4)
C20 C19 C24	117.8(3)	C13B C18B Cr6	72.4(3)
C20 C19 C2	121.9(3)	C24B C19B C20B	119.0(3)
C24 C19 C2	120.3(3)	C24B C19B C2B	122.1(3)
C20 C19 Cr2	70.71(19)	C20B C19B C2B	118.9(3)
C24 C19 Cr2	70.02(19)	C24B C19B Cr5	70.5(2)
C2 C19 Cr2	131.4(2)	C20B C19B Cr5	70.2(2)
C21 C20 C19	121.3(4)	C2B C19B Cr5	132.8(2)
C21 C20 Cr2	71.9(2)	C21B C20B C19B	120.3(4)
C19 C20 Cr2	72.02(18)	C21B C20B Cr5	71.8(3)
C22 C21 C20	119.0(4)	C19B C20B Cr5	72.2(2)
C22 C21 Cr2	71.1(2)	C22B C21B C20B	120.1(4)
C20 C21 Cr2	71.0(2)	C22B C21B Cr5	71.4(3)
C23 C22 C21	121.0(4)	C20B C21B Cr5	71.2(2)
C23 C22 Cr2	71.8(2)	C21B C22B C23B	120.1(4)
C21 C22 Cr2	71.7(3)	C21B C22B Cr5	71.3(3)
C22 C23 C24	119.4(4)	C23B C22B Cr5	71.5(3)
C22 C23 Cr2	71.6(3)	C22B C23B C24B	119.8(4)
C24 C23 Cr2	70.5(2)	C22B C23B Cr5	71.8(3)
C23 C24 C19	121.5(4)	C24B C23B Cr5	70.9(2)
C23 C24 Cr2	72.3(2)	C19B C24B C23B	120.6(4)

C19 C24 Cr2	72.7(2)	C19B C24B Cr5	72.8(2)
O1 C25 Cr1	178.1(6)	C23B C24B Cr5	71.4(2)
O2 C26 Cr1	178.8(4)	O1B C25B Cr6	177.5(4)
O3 C27 Cr1	178.4(6)	O2B C26B Cr6	177.6(8)
O4 C28 Cr2	177.1(7)	O3B C27B Cr6	176.1(9)
O5 C29 Cr2	178.3(4)	O4B C28B Cr5	178.2(5)
O6 C30 Cr2	177.7(5)	O5B C29B Cr5	178.6(5)
C27A Cr1A C25A	85.9(3)	O6B C30B Cr5	177.4(6)
C27A Cr1A C26A	90.59(19)	C30A Cr7 C28A	86.7(2)
C25A Cr1A C26A	86.9(2)	C30A Cr7 C29A	87.1(3)
C27A Cr1A C14A	133.86(15)	C28A Cr7 C29A	90.3(2)
C25A Cr1A C14A	88.0(2)	C30A Cr7 C24A	89.4(2)
C26A Cr1A C14A	134.68(18)	C28A Cr7 C24A	134.13(18)
C27A Cr1A C18A	87.24(19)	C29A Cr7 C24A	135.15(19)
C25A Cr1A C18A	137.05(17)	C30A Cr7 C22A	135.9(3)
C26A Cr1A C18A	135.6(2)	C28A Cr7 C22A	136.4(3)
C14A Cr1A C18A	67.24(14)	C29A Cr7 C22A	84.5(2)
C27A Cr1A C17A	103.7(2)	C24A Cr7 C22A	67.8(2)
C25A Cr1A C17A	167.0(2)	C30A Cr7 C23A	103.3(3)
C26A Cr1A C17A	101.5(2)	C28A Cr7 C23A	166.1(2)
C14A Cr1A C17A	79.05(15)	C29A Cr7 C23A	99.74(19)
C18A Cr1A C17A	37.23(14)	C24A Cr7 C23A	38.26(19)
C27A Cr1A C16A	137.7(2)	C22A Cr7 C23A	36.9(2)
C25A Cr1A C16A	135.9(2)	C30A Cr7 C20A	139.7(2)
C26A Cr1A C16A	86.11(19)	C28A Cr7 C20A	87.66(19)
C14A Cr1A C16A	67.26(15)	C29A Cr7 C20A	132.8(2)
C18A Cr1A C16A	67.04(15)	C24A Cr7 C20A	66.94(17)
C17A Cr1A C16A	36.78(17)	C22A Cr7 C20A	65.9(2)
C27A Cr1A C15A	166.31(18)	C23A Cr7 C20A	78.4(2)
C25A Cr1A C15A	102.8(2)	C30A Cr7 C21A	168.7(2)
C26A Cr1A C15A	100.32(18)	C28A Cr7 C21A	103.0(2)
C14A Cr1A C15A	37.82(14)	C29A Cr7 C21A	98.6(2)
C18A Cr1A C15A	79.19(16)	C24A Cr7 C21A	79.7(2)
C17A Cr1A C15A	66.16(18)	C22A Cr7 C21A	36.2(2)
C16A Cr1A C15A	36.64(17)	C23A Cr7 C21A	66.2(2)
C27A Cr1A C13A	100.33(14)	C20A Cr7 C21A	37.06(18)
C25A Cr1A C13A	102.58(16)	C30A Cr7 C19A	105.1(2)
C26A Cr1A C13A	165.95(18)	C28A Cr7 C19A	101.13(17)
C14A Cr1A C13A	37.27(12)	C29A Cr7 C19A	163.63(18)
C18A Cr1A C13A	37.71(12)	C24A Cr7 C19A	36.76(15)
C17A Cr1A C13A	67.51(13)	C22A Cr7 C19A	79.17(17)
C16A Cr1A C13A	79.87(13)	C23A Cr7 C19A	67.18(16)
C15A Cr1A C13A	67.69(12)	C20A Cr7 C19A	37.64(14)
C25C Cr2A C27C	89.4(3)	C21A Cr7 C19A	67.62(16)
C25C Cr2A C26C	89.54(19)	C30C Cr8 C29C	87.5(2)
C27C Cr2A C26C	84.9(2)	C30C Cr8 C28C	87.9(3)
C25C Cr2A C14C	87.2(2)	C29C Cr8 C28C	89.0(2)
C27C Cr2A C14C	125.59(19)	C30C Cr8 C24C	87.0(2)
C26C Cr2A C14C	149.21(16)	C29C Cr8 C24C	134.55(18)

C25C Cr2A C15C	90.7(2)	C28C Cr8 C24C	135.79(19)
C27C Cr2A C15C	163.20(19)	C30C Cr8 C20C	137.3(2)
C26C Cr2A C15C	111.85(16)	C29C Cr8 C20C	134.77(16)
C14C Cr2A C15C	37.68(14)	C28C Cr8 C20C	87.5(2)
C25C Cr2A C18C	150.14(17)	C24C Cr8 C20C	67.82(15)
C27C Cr2A C18C	92.41(19)	C30C Cr8 C22C	135.4(3)
C26C Cr2A C18C	120.31(14)	C29C Cr8 C22C	86.28(19)
C14C Cr2A C18C	67.71(13)	C28C Cr8 C22C	136.0(2)
C15C Cr2A C18C	79.33(15)	C24C Cr8 C22C	67.24(18)
C25C Cr2A C16C	118.2(3)	C20C Cr8 C22C	66.29(17)
C27C Cr2A C16C	151.5(2)	C30C Cr8 C23C	101.9(3)
C26C Cr2A C16C	88.06(17)	C29C Cr8 C23C	100.38(19)
C14C Cr2A C16C	66.91(15)	C28C Cr8 C23C	166.7(2)
C15C Cr2A C16C	36.05(17)	C24C Cr8 C23C	37.68(15)
C18C Cr2A C16C	67.56(15)	C20C Cr8 C23C	79.13(18)
C25C Cr2A C17C	155.5(3)	C22C Cr8 C23C	36.8(2)
C27C Cr2A C17C	115.1(2)	C30C Cr8 C19C	102.9(2)
C26C Cr2A C17C	91.91(16)	C29C Cr8 C19C	165.13(18)
C14C Cr2A C17C	79.18(15)	C28C Cr8 C19C	101.84(19)
C15C Cr2A C17C	66.17(17)	C24C Cr8 C19C	37.71(13)
C18C Cr2A C17C	37.08(13)	C20C Cr8 C19C	37.43(12)
C16C Cr2A C17C	37.50(17)	C22C Cr8 C19C	78.85(15)
C25C Cr2A C13C	112.60(17)	C23C Cr8 C19C	67.37(14)
C27C Cr2A C13C	97.11(16)	C30C Cr8 C21C	166.4(2)
C26C Cr2A C13C	157.73(14)	C29C Cr8 C21C	100.41(18)
C14C Cr2A C13C	37.49(12)	C28C Cr8 C21C	103.2(2)
C15C Cr2A C13C	67.44(13)	C24C Cr8 C21C	79.62(17)
C18C Cr2A C13C	37.62(11)	C20C Cr8 C21C	37.59(16)
C16C Cr2A C13C	79.56(13)	C22C Cr8 C21C	35.8(2)
C17C Cr2A C13C	67.05(12)	C23C Cr8 C21C	66.0(2)
C1A B3A C2A	60.35(19)	C19C Cr8 C21C	67.39(14)
C1A B3A B9A	105.6(3)	C2C B3C C1C	60.09(19)
C2A B3A B9A	105.3(3)	C2C B3C B8C	58.3(2)
C1A B3A B8A	106.2(3)	C1C B3C B8C	107.0(3)
C2A B3A B8A	57.4(2)	C2C B3C B9C	105.8(3)
B9A B3A B8A	60.4(3)	C1C B3C B9C	106.1(3)
C1A B3A B4A	58.1(2)	B8C B3C B9C	60.4(2)
C2A B3A B4A	106.7(3)	C2C B3C B4C	106.5(3)
B9A B3A B4A	60.5(2)	C1C B3C B4C	58.2(2)
B8A B3A B4A	108.6(3)	B8C B3C B4C	108.7(3)
C1A B4A B3A	59.6(2)	B9C B3C B4C	60.5(3)
C1A B4A B10A	105.9(3)	C1C B4C B9C	106.6(3)
B3A B4A B10A	107.7(3)	C1C B4C B10C	105.1(3)
C1A B4A B5A	58.6(2)	B9C B4C B10C	61.2(3)
B3A B4A B5A	107.4(3)	C1C B4C B5C	58.2(2)
B10A B4A B5A	59.8(3)	B9C B4C B5C	108.4(3)
C1A B4A B9A	106.0(3)	B10C B4C B5C	59.3(3)
B3A B4A B9A	59.4(2)	C1C B4C B3C	59.9(2)
B10A B4A B9A	60.2(3)	B9C B4C B3C	59.5(2)

B5A B4A B9A	107.5(3)	B10C B4C B3C	107.8(3)
C1A B5A B6A	59.9(2)	B5C B4C B3C	107.3(3)
C1A B5A B4A	58.4(2)	C1C B5C B11C	107.3(3)
B6A B5A B4A	108.0(3)	C1C B5C B10C	106.4(3)
C1A B5A B10A	106.0(3)	B11C B5C B10C	59.4(3)
B6A B5A B10A	108.7(3)	C1C B5C B6C	60.3(2)
B4A B5A B10A	60.1(3)	B11C B5C B6C	60.2(2)
C1A B5A B11A	106.7(3)	B10C B5C B6C	107.7(3)
B6A B5A B11A	60.0(3)	C1C B5C B4C	58.8(2)
B4A B5A B11A	108.6(3)	B11C B5C B4C	108.1(3)
B10A B5A B11A	60.8(3)	B10C B5C B4C	60.3(3)
C2A B6A C1A	60.72(19)	B6C B5C B4C	108.2(3)
C2A B6A B5A	106.8(3)	C2C B6C C1C	60.3(2)
C1A B6A B5A	58.0(2)	C2C B6C B11C	105.7(3)
C2A B6A B7A	58.3(2)	C1C B6C B11C	103.9(3)
C1A B6A B7A	106.7(3)	C2C B6C B5C	105.9(3)
B5A B6A B7A	107.6(3)	C1C B6C B5C	57.2(2)
C2A B6A B11A	105.2(3)	B11C B6C B5C	58.9(3)
C1A B6A B11A	105.1(3)	C2C B6C B7C	58.9(2)
B5A B6A B11A	59.9(3)	C1C B6C B7C	107.4(3)
B7A B6A B11A	59.4(3)	B11C B6C B7C	61.1(2)
C2A B7A B11A	105.8(3)	B5C B6C B7C	108.6(3)
C2A B7A B12A	106.2(3)	C2C B7C B6C	58.9(2)
B11A B7A B12A	60.4(3)	C2C B7C B12C	104.4(3)
C2A B7A B8A	58.5(2)	B6C B7C B12C	107.3(3)
B11A B7A B8A	108.4(4)	C2C B7C B8C	58.6(2)
B12A B7A B8A	60.7(3)	B6C B7C B8C	107.3(3)
C2A B7A B6A	58.7(2)	B12C B7C B8C	59.1(2)
B11A B7A B6A	59.8(3)	C2C B7C B11C	104.6(3)
B12A B7A B6A	108.0(3)	B6C B7C B11C	59.6(2)
B8A B7A B6A	107.0(3)	B12C B7C B11C	59.7(3)
C2A B8A B9A	106.7(3)	B8C B7C B11C	106.6(3)
C2A B8A B3A	60.3(2)	C2C B8C B3C	59.7(2)
B9A B8A B3A	59.3(2)	C2C B8C B12C	105.5(3)
C2A B8A B7A	59.4(2)	B3C B8C B12C	107.0(3)
B9A B8A B7A	108.5(3)	C2C B8C B7C	58.3(2)
B3A B8A B7A	108.9(3)	B3C B8C B7C	107.7(3)
C2A B8A B12A	106.3(3)	B12C B8C B7C	60.8(3)
B9A B8A B12A	60.5(3)	C2C B8C B9C	105.9(3)
B3A B8A B12A	107.9(3)	B3C B8C B9C	59.2(2)
B7A B8A B12A	59.8(3)	B12C B8C B9C	60.0(3)
B3A B9A B8A	60.3(3)	B7C B8C B9C	108.8(3)
B3A B9A B10A	107.9(3)	B4C B9C B3C	60.1(2)
B8A B9A B10A	107.8(4)	B4C B9C B12C	107.6(3)
B3A B9A B4A	60.0(2)	B3C B9C B12C	107.9(3)
B8A B9A B4A	108.1(3)	B4C B9C B8C	108.4(3)
B10A B9A B4A	59.8(3)	B3C B9C B8C	60.4(2)
B3A B9A B12A	108.1(4)	B12C B9C B8C	59.9(3)
B8A B9A B12A	59.6(3)	B4C B9C B10C	59.4(3)

B10A B9A B12A	60.1(3)	B3C B9C B10C	106.9(3)
B4A B9A B12A	108.0(3)	B12C B9C B10C	59.2(3)
B4A B10A B12A	108.1(4)	B8C B9C B10C	106.9(3)
B4A B10A B9A	60.0(3)	B5C B10C B11C	60.1(3)
B12A B10A B9A	60.1(3)	B5C B10C B4C	60.3(2)
B4A B10A B5A	60.1(3)	B11C B10C B4C	108.6(3)
B12A B10A B5A	107.2(4)	B5C B10C B12C	109.0(3)
B9A B10A B5A	107.5(3)	B11C B10C B12C	61.4(3)
B4A B10A B11A	107.5(3)	B4C B10C B12C	107.8(3)
B12A B10A B11A	59.3(3)	B5C B10C B9C	107.8(3)
B9A B10A B11A	107.1(4)	B11C B10C B9C	108.6(3)
B5A B10A B11A	59.5(3)	B4C B10C B9C	59.4(3)
B12A B11A B7A	59.7(3)	B12C B10C B9C	59.5(3)
B12A B11A B10A	60.3(3)	B5C B11C B6C	60.9(2)
B7A B11A B10A	108.2(4)	B5C B11C B10C	60.5(3)
B12A B11A B6A	108.2(3)	B6C B11C B10C	109.1(3)
B7A B11A B6A	60.8(3)	B5C B11C B7C	108.7(3)
B10A B11A B6A	107.9(3)	B6C B11C B7C	59.3(2)
B12A B11A B5A	108.1(4)	B10C B11C B7C	109.1(3)
B7A B11A B5A	108.8(3)	B5C B11C B12C	108.3(3)
B10A B11A B5A	59.7(3)	B6C B11C B12C	107.6(3)
B6A B11A B5A	60.1(3)	B10C B11C B12C	60.2(3)
B11A B12A B7A	59.9(3)	B7C B11C B12C	60.3(3)
B11A B12A B10A	60.4(3)	B8C B12C B9C	60.1(3)
B7A B12A B10A	108.5(3)	B8C B12C B7C	60.1(2)
B11A B12A B8A	107.1(3)	B9C B12C B7C	108.4(3)
B7A B12A B8A	59.5(3)	B8C B12C B10C	108.7(3)
B10A B12A B8A	107.7(3)	B9C B12C B10C	61.3(3)
B11A B12A B9A	107.7(3)	B7C B12C B10C	107.4(4)
B7A B12A B9A	107.8(3)	B8C B12C B11C	107.7(3)
B10A B12A B9A	59.8(3)	B9C B12C B11C	107.6(3)
B8A B12A B9A	59.9(3)	B7C B12C B11C	60.0(3)
C13A C1A B4A	123.3(3)	B10C B12C B11C	58.4(3)
C13A C1A B5A	124.7(3)	C13C C1C B5C	123.9(3)
B4A C1A B5A	63.0(3)	C13C C1C B4C	124.9(3)
C13A C1A B3A	116.4(3)	B5C C1C B4C	63.0(2)
B4A C1A B3A	62.3(2)	C13C C1C B3C	117.9(2)
B5A C1A B3A	113.0(3)	B5C C1C B3C	112.9(3)
C13A C1A B6A	117.5(3)	B4C C1C B3C	61.9(2)
B4A C1A B6A	113.6(3)	C13C C1C B6C	116.2(3)
B5A C1A B6A	62.1(3)	B5C C1C B6C	62.5(2)
B3A C1A B6A	111.1(2)	B4C C1C B6C	113.3(3)
C13A C1A C2A	116.1(3)	B3C C1C B6C	110.4(2)
B4A C1A C2A	110.1(2)	C13C C1C C2C	115.8(2)
B5A C1A C2A	108.8(2)	B5C C1C C2C	108.9(2)
B3A C1A C2A	60.4(2)	B4C C1C C2C	109.4(3)
B6A C1A C2A	59.15(19)	B3C C1C C2C	60.11(19)
C19A C2A B8A	121.9(3)	B6C C1C C2C	58.76(19)
C19A C2A B7A	125.2(3)	C19C C2C B8C	122.2(3)

B8A C2A B7A	62.1(3)	C19C C2C B7C	126.2(3)
C19A C2A B6A	119.6(3)	B8C C2C B7C	63.1(2)
B8A C2A B6A	113.2(3)	C19C C2C B6C	119.5(3)
B7A C2A B6A	63.0(2)	B8C C2C B6C	113.5(3)
C19A C2A C1A	116.8(2)	B7C C2C B6C	62.1(2)
B8A C2A C1A	109.1(3)	C19C C2C B3C	113.5(3)
B7A C2A C1A	109.9(3)	B8C C2C B3C	62.0(2)
B6A C2A C1A	60.13(19)	B7C C2C B3C	113.5(3)
C19A C2A B3A	115.0(3)	B6C C2C B3C	112.0(3)
B8A C2A B3A	62.2(2)	C19C C2C C1C	114.9(3)
B7A C2A B3A	112.6(3)	B8C C2C C1C	109.7(3)
B6A C2A B3A	110.9(3)	B7C C2C C1C	110.4(3)
C1A C2A B3A	59.25(19)	B6C C2C C1C	60.9(2)
C14A C13A C18A	117.1(3)	B3C C2C C1C	59.8(2)
C14A C13A C1A	123.2(3)	C14C C13C C18C	118.2(3)
C18A C13A C1A	119.7(3)	C14C C13C C1C	120.5(3)
C14A C13A Cr1A	69.78(17)	C18C C13C C1C	121.3(3)
C18A C13A Cr1A	69.52(18)	C14C C13C Cr2A	69.72(19)
C1A C13A Cr1A	129.8(2)	C18C C13C Cr2A	70.08(17)
C13A C14A C15A	121.6(3)	C1C C13C Cr2A	133.6(2)
C13A C14A Cr1A	72.95(18)	C13C C14C C15C	120.1(3)
C15A C14A Cr1A	72.5(2)	C13C C14C Cr2A	72.79(19)
C16A C15A C14A	119.1(4)	C15C C14C Cr2A	71.6(2)
C16A C15A Cr1A	71.0(2)	C16C C15C C14C	121.2(4)
C14A C15A Cr1A	69.7(2)	C16C C15C Cr2A	72.2(2)
C16A C15A H15A	120.4	C14C C15C Cr2A	70.7(2)
C14A C15A H15A	120.4	C15C C16C C17C	119.8(4)
Cr1A C15A H15A	131.6	C15C C16C Cr2A	71.8(2)
C15A C16A C17A	120.4(3)	C17C C16C Cr2A	71.3(2)
C15A C16A Cr1A	72.3(2)	C18C C17C C16C	120.6(3)
C17A C16A Cr1A	71.5(2)	C18C C17C Cr2A	71.20(18)
C18A C17A C16A	120.5(4)	C16C C17C Cr2A	71.2(2)
C18A C17A Cr1A	70.6(2)	C17C C18C C13C	120.0(3)
C16A C17A Cr1A	71.7(2)	C17C C18C Cr2A	71.7(2)
C17A C18A C13A	121.2(3)	C13C C18C Cr2A	72.30(17)
C17A C18A Cr1A	72.2(2)	C24C C19C C20C	119.2(3)
C13A C18A Cr1A	72.78(18)	C24C C19C C2C	122.1(3)
C24A C19A C20A	118.0(4)	C20C C19C C2C	118.7(3)
C24A C19A C2A	122.8(4)	C24C C19C Cr8	70.3(2)
C20A C19A C2A	119.2(3)	C20C C19C Cr8	70.7(2)
C24A C19A Cr7	69.9(2)	C2C C19C Cr8	133.5(2)
C20A C19A Cr7	70.1(2)	C21C C20C C19C	120.0(4)
C2A C19A Cr7	132.5(2)	C21C C20C Cr8	71.9(3)
C21A C20A C19A	121.6(5)	C19C C20C Cr8	71.9(2)
C21A C20A Cr7	72.0(3)	C20C C21C C22C	119.5(4)
C19A C20A Cr7	72.2(2)	C20C C21C Cr8	70.6(2)
C22A C21A C20A	118.8(5)	C22C C21C Cr8	71.2(3)
C22A C21A Cr7	70.5(3)	C23C C22C C21C	122.6(4)
C20A C21A Cr7	71.0(3)	C23C C22C Cr8	72.3(3)

C23A C22A C21A	122.4(5)	C21C C22C Cr8	72.9(3)
C23A C22A Cr7	72.4(3)	C22C C23C C24C	119.4(4)
C21A C22A Cr7	73.3(3)	C22C C23C Cr8	70.9(3)
C22A C23A C24A	119.1(5)	C24C C23C Cr8	70.4(2)
C22A C23A Cr7	70.7(3)	C23C C24C C19C	119.2(4)
C24A C23A Cr7	70.2(2)	C23C C24C Cr8	72.0(2)
C19A C24A C23A	120.0(5)	C19C C24C Cr8	72.0(2)
C19A C24A Cr7	73.3(2)	O1C C25C Cr2A	178.2(5)
C23A C24A Cr7	71.5(2)	O2C C26C Cr2A	178.8(4)
O1A C25A Cr1A	178.6(5)	O3C C27C Cr2A	176.4(6)
O2A C26A Cr1A	177.4(5)	O4C C28C Cr8	179.7(6)
O3A C27A Cr1A	178.9(4)	O5C C29C Cr8	179.1(5)
O4A C28A Cr7	177.7(5)	O6C C30C Cr8	178.1(8)
O5A C29A Cr7	176.9(5)		

Table S9. Torsional angles (º) of **3**.

C2 B3 B4 C1	-37.2(2)	C2B B8B B9B B3B	-38.7(3)
B8 B3 B4 C1	-98.4(3)	B7B B8B B9B B3B	-100.1(3)
B9 B3 B4 C1	-135.9(3)	B12B B8B B9B B3B	-137.3(3)
C2 B3 B4 B5	-2.3(3)	C2B B8B B9B B4B	-1.1(5)
C1 B3 B4 B5	34.9(2)	B7B B8B B9B B4B	-62.5(5)
B8 B3 B4 B5 -	63.5(3)	B12B B8B B9B B4B	-99.7(4)
B9 B3 B4 B5	-100.9(3)	B3B B8B B9B B4B	37.6(3)
C2 B3 B4 B10	60.6(3)	C2B B8B B9B B12B	98.5(4)
C1 B3 B4 B10	97.7(3)	B7B B8B B9B B12B	37.1(3)
B8 B3 B4 B10	-0.7(4)	B3B B8B B9B B12B	137.3(3)
B9 B3 B4 B10	-38.1(3)	B3B B9B B10B B12B	100.3(4)
C2 B3 B4 B9	98.7(3)	B4B B9B B10B B12B	138.1(3)
C1 B3 B4 B9	135.9(3)	B8B B9B B10B B12B	36.4(3)
B8 B3 B4 B9	37.4(3)	B3B B9B B10B B4B	-37.8(3)
B10 B4 B5 C1	-134.5(3)	B8B B9B B10B B4B	-101.7(3)
B9 B4 B5 C1	-97.5(3)	B12B B9B B10B B4B	-138.1(3)
B3 B4 B5 C1	-34.9(2)	B3B B9B B10B B5B	0.4(5)
C1 B4 B5 B10	134.5(3)	B4B B9B B10B B5B	38.1(3)
B9 B4 B5 B10	37.0(3)	B8B B9B B10B B5B	-63.5(5)
B3 B4 B5 B10	99.6(3)	B12B B9B B10B B5B	-100.0(4)
C1 B4 B5 B6	34.4(2)	B3B B9B B10B B11B	62.8(5)
B10 B4 B5 B6	-100.1(3)	B4B B9B B10B B11B	100.5(4)
B9 B4 B5 B6	-63.1(3)	B8B B9B B10B B11B	-1.1(5)
B3 B4 B5 B6	-0.5(3)	B12B B9B B10B B11B	-37.6(3)
C1 B4 B5 B11	97.2(3)	C1B B4B B10B B9B	99.4(3)
B10 B4 B5 B11	-37.3(3)	B5B B4B B10B B9B	137.3(3)
B9 B4 B5 B11	-0.3(4)	B3B B4B B10B B9B	37.2(3)
B3 B4 B5 B11	62.3(3)	C1B B4B B10B B12B	61.7(4)
B10 B5 B6 C1	-97.0(3)	B9B B4B B10B B12B	-37.7(3)
B4 B5 B6 C1	-34.1(2)	B5B B4B B10B B12B	99.6(4)
B11 B5 B6 C1	-135.7(3)	B3B B4B B10B B12B	-0.5(4)
C1 B5 B6 C2	37.2(2)	C1B B4B B10B B5B	-37.9(3)
B10 B5 B6 C2	-59.8(3)	B9B B4B B10B B5B	-137.3(3)
B4 B5 B6 C2	3.1(3)	B3B B4B B10B B5B	-137.3(3)
B11 B5 B6 C2	-98.5(3)	C1B B4B B10B B11B	-1.3(4)
C1 B5 B6 B11	135.7(3)	B9B B4B B10B B11B	-100.7(4)
B10 B5 B6 B11	38.7(3)	B5B B4B B10B B11B	36.6(3)
B4 B5 B6 B11	101.6(3)	B3B B4B B10B B11B	-63.5(4)
C1 B5 B6 B7	98.6(3)	C1B B5B B10B B9B	-0.2(5)
B10 B5 B6 B7	1.6(3)	B11B B5B B10B B9B	100.3(4)
B4 B5 B6 B7	64.5(3)	B4B B5B B10B B9B	-38.2(4)
B11 B5 B6 B7	-37.1(3)	B6B B5B B10B B9B	62.1(5)
C1 B6 B7 C2	-37.1(2)	C1B B5B B10B B12B	-63.8(5)
B11 B6 B7 C2	-137.0(3)	B11B B5B B10B B12B	36.7(4)
B5 B6 B7 C2	-99.7(3)	B4B B5B B10B B12B	-101.7(4)
C1 B6 B7 B11	99.9(3)	B6B B5B B10B B12B	-1.5(5)
C2 B6 B7 B11	137.0(3)	C1B B5B B10B B4B	38.0(3)

B5 B6 B7 B11	37.3(3)	B11B B5B B10B B4B	138.5(3)
C1 B6 B7 B8	-2.5(3)	B6B B5B B10B B4B	100.3(3)
C2 B6 B7 B8	34.6(2)	C1B B5B B10B B11B	-100.5(3)
B11 B6 B7 B8	-102.4(3)	B4B B5B B10B B11B	-138.5(3)
B5 B6 B7 B8	-65.1(3)	B6B B5B B10B B11B	-38.2(3)
C1 B6 B7 B12	61.5(3)	C2B B7B B11B B12B	-99.7(4)
C2 B6 B7 B12	98.6(3)	B6B B7B B11B B12B	-137.9(4)
B11 B6 B7 B12	-38.4(3)	B8B B7B B11B B12B	-37.6(4)
B5 B6 B7 B12	-1.1(3)	C2B B7B B11B B5B	0.7(4)
C1 B3 B8 C2	36.9(2)	B12B B7B B11B B5B	100.5(4)
B9 B3 B8 C2	135.1(3)	B6B B7B B11B B5B	-37.5(3)
B4 B3 B8 C2	97.7(3)	B8B B7B B11B B5B	62.8(4)
C2 B3 B8 B9	-135.1(3)	C2B B7B B11B B6B	38.2(3)
C1 B3 B8 B9	-98.2(3)	B12B B7B B11B B6B	137.9(4)
B4 B3 B8 B9	-37.4(3)	B8B B7B B11B B6B	100.3(4)
C2 B3 B8 B7	-34.6(2)	C2B B7B B11B B10B	-62.7(4)
C1 B3 B8 B7	2.3(3)	B12B B7B B11B B10B	37.0(4)
B9 B3 B8 B7	100.5(3)	B6B B7B B11B B10B	-100.9(4)
B4 B3 B8 B7	63.1(3)	B8B B7B B11B B10B	-0.6(5)
C2 B3 B8 B12	-97.2(3)	C1B B5B B11B B12B	62.2(4)
C1 B3 B8 B12	-60.3(3)	B4B B5B B11B B12B	0.3(4)
B9 B3 B8 B12	38.0(3)	B6B B5B B11B B12B	100.0(3)
B4 B3 B8 B12	0.6(4)	B10B B5B B11B B12B	-37.1(3)
B11 B7 B8 C2	-98.0(3)	C1B B5B B11B B7B	-0.8(4)
B6 B7 B8 C2	-34.9(2)	B4B B5B B11B B7B	-62.7(4)
B12 B7 B8 C2	-135.6(3)	B6B B5B B11B B7B	37.0(3)
C2 B7 B8 B9	97.6(3)	B10B B5B B11B B7B	-100.1(4)
B11 B7 B8 B9	-0.3(3)	C1B B5B B11B B6B	-37.9(3)
B6 B7 B8 B9	62.7(3)	B4B B5B B11B B6B	-99.8(3)
B12 B7 B8 B9	-37.9(2)	B10B B5B B11B B6B	-137.1(3)
C2 B7 B8 B3	35.0(2)	C1B B5B B11B B10B	99.2(3)
B11 B7 B8 B3	-63.0(3)	B4B B5B B11B B10B	37.3(3)
B6 B7 B8 B3	0.1(4)	B6B B5B B11B B10B	137.1(3)
B12 B7 B8 B3	-100.6(3)	C2B B6B B11B B12B	-0.6(5)
C2 B7 B8 B12	135.6(3)	C1B B6B B11B B12B	-64.3(4)
B11 B7 B8 B12	37.6(3)	B7B B6B B11B B12B	37.2(4)
B6 B7 B8 B12	100.7(3)	B5B B6B B11B B12B	-101.4(4)
C2 B8 B9 B3	-39.0(2)	C2B B6B B11B B7B	-37.8(3)
B7 B8 B9 B3	-100.2(3)	C1B B6B B11B B7B	-101.5(3)
B12 B8 B9 B3	-137.7(3)	B5B B6B B11B B7B	-138.6(3)
C2 B8 B9 B12	98.7(3)	C2B B6B B11B B5B	100.8(3)
B3 B8 B9 B12	137.7(3)	C1B B6B B11B B5B	37.1(3)
B7 B8 B9 B12	37.4(2)	B7B B6B B11B B5B	138.6(3)
C2 B8 B9 B4	-1.1(3)	C2B B6B B11B B10B	62.5(4)
B3 B8 B9 B4	37.9(3)	C1B B6B B11B B10B	-1.2(4)
B7 B8 B9 B4	-62.3(3)	B7B B6B B11B B10B	100.3(4)
B12 B8 B9 B4	-99.8(3)	B5B B6B B11B B10B	-38.3(3)
C2 B8 B9 B10	62.0(3)	B9B B10B B11B B12B	37.9(4)
B3 B8 B9 B10	101.0(3)	B4B B10B B11B B12B	101.4(4)

B7 B8 B9 B10	0.8(3)	B5B B10B B11B B12B	138.5(4)
B12 B8 B9 B10	-36.6(3)	B9B B10B B11B B7B	1.1(5)
C2 B3 B9 B8	38.5(2)	B12B B10B B11B B7B	-36.8(3)
C1 B3 B9 B8	100.7(3)	B4B B10B B11B B7B	64.6(4)
B4 B3 B9 B8	138.3(3)	B5B B10B B11B B7B	101.6(4)
C2 B3 B9 B12	0.5(4)	B9B B10B B11B B5B	-100.6(4)
C1 B3 B9 B12	62.8(3)	B12B B10B B11B B5B	-138.5(4)
B8 B3 B9 B12	-37.9(3)	B4B B10B B11B B5B	-37.1(3)
B4 B3 B9 B12	100.4(3)	B9B B10B B11B B6B	-61.9(4)
C2 B3 B9 B4	-99.8(3)	B12B B10B B11B B6B	-99.9(4)
C1 B3 B9 B4	-37.6(2)	B4B B10B B11B B6B	1.6(4)
B8 B3 B9 B4	-138.3(3)	B5B B10B B11B B6B	38.6(3)
C2 B3 B9 B10	-61.9(3)	B5B B11B B12B B7B	-101.3(4)
C1 B3 B9 B10	0.4(3)	B6B B11B B12B B7B	-37.2(3)
B8 B3 B9 B10	-100.3(3)	B10B B11B B12B B7B	-138.4(4)
B4 B3 B9 B10	38.0(3)	B7B B11B B12B B10B	138.4(4)
C1 B4 B9 B8	0.8(4)	B5B B11B B12B B10B	37.1(3)
B5 B4 B9 B8	63.0(3)	B6B B11B B12B B10B	101.2(4)
B10 B4 B9 B8	99.6(3)	B7B B11B B12B B8B	36.6(4)
B3 B4 B9 B8	-37.7(2)	B5B B11B B12B B8B	-64.7(5)
C1 B4 B9 B3	38.4(2)	B6B B11B B12B B8B	-0.7(6)
B5 B4 B9 B3	100.7(3)	B10B B11B B12B B8B	-101.9(5)
B10 B4 B9 B3	137.3(3)	B7B B11B B12B B9B	100.5(4)
C1 B4 B9 B12	-62.9(3)	B5B B11B B12B B9B	-0.8(4)
B5 B4 B9 B12	-0.7(4)	B6B B11B B12B B9B	63.3(4)
B10 B4 B9 B12	35.9(3)	B10B B11B B12B B9B	-37.9(3)
B3 B4 B9 B12	-101.4(3)	C2B B7B B12B B11B	99.6(4)
C1 B4 B9 B10	-98.8(3)	B6B B7B B12B B11B	37.4(4)
B5 B4 B9 B10	-36.6(3)	B8B B7B B12B B11B	138.8(4)
B3 B4 B9 B10	-137.3(3)	C2B B7B B12B B10B	61.9(5)
C1 B5 B10 B12	-62.8(3)	B11B B7B B12B B10B	-37.7(4)
B4 B5 B10 B12	-101.4(3)	B6B B7B B12B B10B	-0.3(5)
B6 B5 B10 B12	-1.6(4)	B8B B7B B12B B10B	101.1(5)
B11 B5 B10 B12	36.9(3)	C2B B7B B12B B8B	-39.2(3)
C1 B5 B10 B11	-99.7(3)	B11B B7B B12B B8B	-138.8(4)
B4 B5 B10 B11	-138.4(3)	B6B B7B B12B B8B	-101.4(4)
B6 B5 B10 B11	-38.5(2)	C2B B7B B12B B9B	-1.7(4)
C1 B5 B10 B4	38.7(2)	B11B B7B B12B B9B	-101.3(4)
B6 B5 B10 B4	99.9(3)	B6B B7B B12B B9B	-63.9(4)
B11 B5 B10 B4	138.4(3)	B8B B7B B12B B9B	37.5(3)
C1 B5 B10 B9	1.8(4)	B9B B10B B12B B11B	-137.6(3)
B4 B5 B10 B9	-36.9(3)	B4B B10B B12B B11B	-100.0(4)
B6 B5 B10 B9	63.0(3)	B5B B10B B12B B11B	-36.6(3)
B11 B5 B10 B9	101.5(3)	B9B B10B B12B B7B	-100.0(4)
C1 B4 B10 B12	62.5(4)	B4B B10B B12B B7B	-62.4(5)
B5 B4 B10 B12	102.0(3)	B5B B10B B12B B7B	1.1(5)
B9 B4 B10 B12	-37.0(3)	B11B B10B B12B B7B	37.6(4)
B3 B4 B10 B12	0.6(4)	B9B B10B B12B B8B	-36.9(3)
C1 B4 B10 B5	-39.5(3)	B4B B10B B12B B8B	0.7(5)

B9 B4 B10 B5	-139.0(3)	B5B B10B B12B B8B	64.1(5)
B3 B4 B10 B5	-101.4(3)	B11B B10B B12B B8B	100.7(4)
C1 B4 B10 B11	-1.9(4)	B4B B10B B12B B9B	37.6(3)
B5 B4 B10 B11	37.5(3)	B5B B10B B12B B9B	101.1(4)
B9 B4 B10 B11	-101.4(3)	B11B B10B B12B B9B	137.6(3)
B3 B4 B10 B11	-63.9(4)	C2B B8B B12B B11B	1.7(5)
C1 B4 B10 B9	99.5(3)	B7B B8B B12B B11B	-37.1(4)
B5 B4 B10 B9	139.0(3)	B3B B8B B12B B11B	63.7(5)
B3 B4 B10 B9	37.5(3)	B9B B8B B12B B11B	101.0(4)
B8 B9 B10 B12	37.1(3)	C2B B8B B12B B7B	38.8(3)
B3 B9 B10 B12	100.8(3)	B3B B8B B12B B7B	100.8(3)
B4 B9 B10 B12	139.3(3)	B9B B8B B12B B7B	138.1(3)
B8 B9 B10 B5	-65.1(3)	C2B B8B B12B B10B	-62.6(5)
B3 B9 B10 B5	-1.4(4)	B7B B8B B12B B10B	-101.4(4)
B12 B9 B10 B5	-102.2(3)	B3B B8B B12B B10B	-0.6(5)
B4 B9 B10 B5	37.1(3)	B9B B8B B12B B10B	36.8(3)
B8 B9 B10 B11	-1.0(3)	C2B B8B B12B B9B	-99.4(4)
B3 B9 B10 B11	62.7(3)	B7B B8B B12B B9B	-138.1(3)
B12 B9 B10 B11	-38.1(3)	B3B B8B B12B B9B	-37.4(3)
B4 B9 B10 B11	101.2(3)	B10B B9B B12B B11B	38.1(3)
B8 B9 B10 B4	-102.2(3)	B3B B9B B12B B11B	-62.7(4)
B3 B9 B10 B4	-38.5(3)	B4B B9B B12B B11B	1.0(4)
B12 B9 B10 B4	-139.3(3)	B8B B9B B12B B11B	-101.0(3)
C1 B6 B11 B7	-99.9(3)	B10B B9B B12B B7B	102.1(4)
C2 B6 B11 B7	-37.0(2)	B3B B9B B12B B7B	1.3(4)
B5 B6 B11 B7	-138.7(3)	B4B B9B B12B B7B	64.9(4)
C1 B6 B11 B10	0.4(4)	B8B B9B B12B B7B	-37.0(3)
C2 B6 B11 B10	63.3(3)	B3B B9B B12B B10B	-100.9(3)
B5 B6 B11 B10	-38.4(3)	B4B B9B B12B B10B	-37.2(3)
B7 B6 B11 B10	100.3(3)	B8B B9B B12B B10B	-139.1(3)
C1 B6 B11 B5	38.9(3)	B10B B9B B12B B8B	139.1(3)
C2 B6 B11 B5	101.8(3)	B3B B9B B12B B8B	38.3(3)
B7 B6 B11 B5	138.7(3)	B4B B9B B12B B8B	102.0(3)
C1 B6 B11 B12	-62.0(3)	B11B B5B C1B C13B	143.0(4)
C2 B6 B11 B12	0.9(3)	B4B B5B C1B C13B	-114.7(4)
B5 B6 B11 B12	-100.9(3)	B6B B5B C1B C13B	105.0(4)
B7 B6 B11 B12	37.8(3)	B10B B5B C1B C13B	-153.3(4)
C2 B7 B11 B6	37.7(2)	B11B B5B C1B B4B	-102.3(4)
B8 B7 B11 B6	99.6(3)	B6B B5B C1B B4B	-140.4(3)
B12 B7 B11 B6	137.2(3)	B10B B5B C1B B4B	-38.6(3)
C2 B7 B11 B10	-62.2(3)	B11B B5B C1B B3B	-63.7(4)
B6 B7 B11 B10	-99.9(3)	B4B B5B C1B B3B	38.6(3)
B8 B7 B11 B10	-0.3(3)	B6B B5B C1B B3B	-101.8(3)
B12 B7 B11 B10	37.3(2)	B10B B5B C1B B3B	0.0(4)
C2 B7 B11 B5	0.7(4)	B11B B5B C1B B6B	38.1(3)
B6 B7 B11 B5	-37.0(3)	B4B B5B C1B B6B	140.4(3)
B8 B7 B11 B5	62.6(4)	B10B B5B C1B B6B	101.8(4)
B12 B7 B11 B5	100.2(3)	B11B B5B C1B C2B	0.6(4)
C2 B7 B11 B12	-99.5(3)	B4B B5B C1B C2B	102.9(3)

B6 B7 B11 B12	-137.2(3)	B6B B5B C1B C2B	-37.5(2)
B8 B7 B11 B12	-37.6(3)	B10B B5B C1B C2B	64.3(4)
B12 B10 B11 B6	-100.9(3)	B9B B4B C1B C13B	-144.4(4)
B5 B10 B11 B6	38.6(3)	B10B B4B C1B C13B	152.9(4)
B4 B10 B11 B6	0.9(4)	B5B B4B C1B C13B	114.4(4)
B9 B10 B11 B6	-62.7(4)	B3B B4B C1B C13B	-105.9(4)
B12 B10 B11 B7	-37.4(2)	B9B B4B C1B B5B	101.2(4)
B5 B10 B11 B7	102.1(3)	B10B B4B C1B B5B	38.5(3)
B4 B10 B11 B7	64.5(4)	B3B B4B C1B B5B	139.7(4)
B9 B10 B11 B7	0.8(3)	B9B B4B C1B B3B	-38.5(3)
B12 B10 B11 B5	-139.4(3)	B10B B4B C1B B3B	-101.2(4)
B4 B10 B11 B5	-37.6(3)	B5B B4B C1B B3B	-139.7(4)
B9 B10 B11 B5	-101.3(3)	B9B B4B C1B B6B	63.3(4)
B5 B10 B11 B12	139.4(3)	B10B B4B C1B B6B	0.5(4)
B4 B10 B11 B12	101.8(3)	B5B B4B C1B B6B	-37.9(3)
B9 B10 B11 B12	38.2(3)	B3B B4B C1B B6B	101.8(3)
C1 B5 B11 B6	-38.3(3)	B9B B4B C1B C2B	-0.3(4)
B10 B5 B11 B6	-136.3(3)	B10B B4B C1B C2B	-63.0(4)
B4 B5 B11 B6	-99.1(3)	B5B B4B C1B C2B	-101.5(3)
C1 B5 B11 B7	-1.0(4)	B3B B4B C1B C2B	38.2(3)
B10 B5 B11 B7	-99.0(3)	C2B B3B C1B C13B	-105.8(3)
B4 B5 B11 B7	-61.8(4)	B9B B3B C1B C13B	155.4(3)
B6 B5 B11 B7	37.3(3)	B4B B3B C1B C13B	116.6(4)
C1 B5 B11 B10	98.1(3)	B8B B3B C1B C13B	-141.3(3)
B4 B5 B11 B10	37.3(3)	C2B B3B C1B B5B	99.0(3)
B6 B5 B11 B10	136.3(3)	B9B B3B C1B B5B	0.2(4)
C1 B5 B11 B12	62.0(3)	B4B B3B C1B B5B	-38.6(4)
B10 B5 B11 B12	-36.0(3)	B8B B3B C1B B5B	63.5(4)
B4 B5 B11 B12	1.2(4)	C2B B3B C1B B4B	137.6(3)
B6 B5 B11 B12	100.3(3)	B9B B3B C1B B4B	38.8(4)
B5 B10 B12 B9	101.1(3)	B8B B3B C1B B4B	102.1(4)
B11 B10 B12 B9	137.9(3)	C2B B3B C1B B6B	31.4(3)
B4 B10 B12 B9	36.7(3)	B9B B3B C1B B6B	-67.4(4)
B5 B10 B12 B7	0.9(4)	B4B B3B C1B B6B	-106.2(3)
B11 B10 B12 B7	37.7(2)	B8B B3B C1B B6B	-4.1(4)
B4 B10 B12 B7	-63.5(4)	B9B B3B C1B C2B	-98.8(4)
B9 B10 B12 B7	-100.2(3)	B4B B3B C1B C2B	-137.6(3)
B5 B10 B12 B8	64.2(4)	B8B B3B C1B C2B	-35.5(2)
B11 B10 B12 B8	101.0(3)	C2B B6B C1B C13B	105.8(3)
B4 B10 B12 B8	-0.2(4)	B7B B6B C1B C13B	142.4(3)
B9 B10 B12 B8	-36.9(3)	B11B B6B C1B C13B	-154.2(3)
B5 B10 B12 B11	-36.8(3)	B5B B6B C1B C13B	-154.2(3)
B4 B10 B12 B11	-101.2(3)	C2B B6B C1B B5B	-137.7(3)
B9 B10 B12 B11	-137.9(3)	B7B B6B C1B B5B	-101.0(3)
B8 B9 B12 B10	-138.4(3)	B11B B6B C1B B5B	-37.6(3)
B3 B9 B12 B10	-100.4(3)	C2B B6B C1B B4B	-99.6(3)
B4 B9 B12 B10	-36.2(3)	B7B B6B C1B B4B	-63.0(4)
B8 B9 B12 B7	-37.7(3)	B11B B6B C1B B4B	0.4(4)
B3 B9 B12 B7	0.4(4)	B5B B6B C1B B4B	38.0(3)

B4 B9 B12 B7	64.6(4)	C2B B6B C1B B3B	-31.7(3)
B10 B9 B12 B7	100.8(3)	B7B B6B C1B B3B	5.0(4)
B3 B9 B12 B8	38.0(3)	B11B B6B C1B B3B	68.3(3)
B4 B9 B12 B8	102.2(3)	B5B B6B C1B B3B	106.0(3)
B10 B9 B12 B8	138.4(3)	B7B B6B C1B C2B	36.6(2)
B8 B9 B12 B11	-100.8(3)	B11B B6B C1B C2B	100.0(3)
B3 B9 B12 B11	-62.8(4)	B5B B6B C1B C2B	137.7(3)
B4 B9 B12 B11	1.5(4)	B11B B7B C2B C19B	-146.3(3)
B10 B9 B12 B11	37.6(3)	B12B B7B C2B C19B	151.0(3)
C2 B7 B12 B10	62.4(3)	B6B B7B C2B C19B	-107.8(3)
B11 B7 B12 B10	-37.8(3)	B8B B7B C2B C19B	110.7(4)
B6 B7 B12 B10	0.1(3)	B11B B7B C2B B6B	-38.5(3)
B8 B7 B12 B10	100.9(3)	B12B B7B C2B B6B	-101.3(3)
C2 B7 B12 B9	-1.1(4)	B8B B7B C2B B6B	-141.5(3)
B11 B7 B12 B9	-101.3(3)	B11B B7B C2B B8B	103.0(4)
B6 B7 B12 B9	-63.4(4)	B12B B7B C2B B8B	40.2(3)
B8 B7 B12 B9	37.4(3)	B6B B7B C2B B8B	141.5(3)
C2 B7 B12 B8	-38.5(2)	B11B B7B C2B B3B	64.3(4)
B11 B7 B12 B8	-138.6(3)	B12B B7B C2B B3B	1.5(4)
B6 B7 B12 B8	-100.8(3)	B6B B7B C2B B3B	102.8(3)
C2 B7 B12 B11	100.1(3)	B8B B7B C2B B3B	-38.7(3)
B6 B7 B12 B11	37.9(2)	B11B B7B C2B C1B	-0.4(4)
B8 B7 B12 B11	138.6(3)	B12B B7B C2B C1B	-63.1(3)
C2 B8 B12 B10	-62.2(4)	B6B B7B C2B C1B	38.1(3)
B9 B8 B12 B10	37.3(3)	B8B B7B C2B C1B	-103.4(3)
B3 B8 B12 B10	-0.2(4)	C1B B6B C2B C19B	-104.2(3)
B7 B8 B12 B10	-100.4(3)	B7B B6B C2B C19B	117.9(4)
C2 B8 B12 B9	-99.5(3)	B11B B6B C2B C19B	156.7(3)
B3 B8 B12 B9	-37.5(3)	B5B B6B C2B C19B	-141.0(3)
B7 B8 B12 B9	-137.6(3)	C1B B6B C2B B7B	137.9(3)
C2 B8 B12 B7	38.1(2)	B11B B6B C2B B7B	38.8(3)
B9 B8 B12 B7	137.6(3)	B5B B6B C2B B7B	101.1(3)
B3 B8 B12 B7	100.1(3)	C1B B6B C2B B8B	100.9(3)
C2 B8 B12 B11	1.2(4)	B7B B6B C2B B8B	-37.1(3)
B9 B8 B12 B11	100.7(3)	B11B B6B C2B B8B	1.8(4)
B3 B8 B12 B11	63.2(4)	B5B B6B C2B B8B	64.1(4)
B7 B8 B12 B11	-37.0(3)	C1B B6B C2B B3B	31.9(3)
B6 B11 B12 B10	99.3(3)	B7B B6B C2B B3B	-106.0(3)
B7 B11 B12 B10	137.6(3)	B11B B6B C2B B3B	-67.2(4)
B5 B11 B12 B10	36.1(3)	B5B B6B C2B B3B	-4.9(4)
B6 B11 B12 B9	61.5(4)	B7B B6B C2B C1B	-137.9(3)
B7 B11 B12 B9	99.8(3)	B11B B6B C2B C1B	-99.1(3)
B10 B11 B12 B9	-37.8(3)	B5B B6B C2B C1B	-36.8(3)
B5 B11 B12 B9	-1.7(4)	B7B B8B C2B C19B	-117.2(4)
B6 B11 B12 B7	-38.3(2)	B12B B8B C2B C19B	-156.4(4)
B10 B11 B12 B7	-137.6(3)	B3B B8B C2B C19B	102.9(4)
B5 B11 B12 B7	-101.5(3)	B9B B8B C2B C19B	141.4(3)
B6 B11 B12 B8	-1.3(4)	B12B B8B C2B B7B	-39.2(3)
B7 B11 B12 B8	37.0(3)	B3B B8B C2B B7B	-139.9(3)

B10 B11 B12 B8	-100.6(3)	B9B B8B C2B B7B	-101.5(4)
B5 B11 B12 B8	-64.5(4)	B7B B8B C2B B6B	37.1(3)
B5 B4 C1 C13	116.2(3)	B12B B8B C2B B6B	-2.2(5)
B10 B4 C1 C13	155.8(3)	B3B B8B C2B B6B	-102.9(3)
B9 B4 C1 C13	-141.3(3)	B9B B8B C2B B6B	-64.4(4)
B3 B4 C1 C13	-103.0(3)	B7B B8B C2B B3B	139.9(3)
B5 B4 C1 B6	-37.3(3)	B12B B8B C2B B3B	100.7(4)
B10 B4 C1 B6	2.3(4)	B9B B8B C2B B3B	38.5(3)
B9 B4 C1 B6	65.2(3)	B7B B8B C2B C1B	102.4(3)
B3 B4 C1 B6	103.5(3)	B12B B8B C2B C1B	63.2(4)
B10 B4 C1 B5	39.6(3)	B3B B8B C2B C1B	-37.5(3)
B9 B4 C1 B5	102.5(3)	B9B B8B C2B C1B	0.9(4)
B3 B4 C1 B5	140.8(3)	C1B B3B C2B C19B	106.5(3)
B5 B4 C1 C2	-102.5(2)	B9B B3B C2B C19B	-154.0(3)
B10 B4 C1 C2	-63.0(3)	B4B B3B C2B C19B	143.2(3)
B9 B4 C1 C2	-0.1(3)	B8B B3B C2B C19B	-114.8(3)
B3 B4 C1 C2	38.2(2)	C1B B3B C2B B7B	-100.2(3)
B5 B4 C1 B3	-140.8(3)	B9B B3B C2B B7B	-0.8(4)
B10 B4 C1 B3	-101.2(3)	B4B B3B C2B B7B	-63.6(4)
B9 B4 C1 B3	-38.3(2)	B8B B3B C2B B7B	38.4(3)
C2 B6 C1 C13	105.1(3)	C1B B3B C2B B6B	-32.0(3)
B11 B6 C1 C13	-155.9(3)	B9B B3B C2B B6B	67.4(4)
B5 B6 C1 C13	-116.5(3)	B4B B3B C2B B6B	4.6(4)
B7 B6 C1 C13	141.3(3)	B8B B3B C2B B6B	106.6(3)
C2 B6 C1 B4	-100.8(3)	C1B B3B C2B B8B	-138.6(3)
B11 B6 C1 B4	-1.7(4)	B9B B3B C2B B8B	-39.2(3)
B5 B6 C1 B4	37.6(3)	B4B B3B C2B B8B	-102.0(4)
B7 B6 C1 B4	-64.6(3)	B9B B3B C2B C1B	99.5(3)
C2 B6 C1 B5	-138.4(3)	B4B B3B C2B C1B	36.6(3)
B11 B6 C1 B5	-39.4(3)	B8B B3B C2B C1B	138.6(3)
B7 B6 C1 B5	-102.2(3)	C13B C1B C2B C19B	3.9(4)
B11 B6 C1 C2	99.0(3)	B5B C1B C2B C19B	149.7(3)
B5 B6 C1 C2	138.4(3)	B4B C1B C2B C19B	-143.5(3)
B7 B6 C1 C2	36.2(2)	B3B C1B C2B C19B	-104.1(3)
C2 B6 C1 B3	-32.1(2)	B6B C1B C2B C19B	110.6(3)
B11 B6 C1 B3	66.9(3)	C13B C1B C2B B7B	-146.0(3)
B5 B6 C1 B3	106.3(3)	B5B C1B C2B B7B	-0.1(3)
B7 B6 C1 B3	4.1(3)	B4B C1B C2B B7B	66.6(4)
B10 B5 C1 C13	-150.9(3)	B3B C1B C2B B7B	106.0(3)
B4 B5 C1 C13	-111.2(3)	B6B C1B C2B B7B	-39.2(3)
B6 B5 C1 C13	108.0(3)	C13B C1B C2B B6B	-106.8(4)
B11 B5 C1 C13	146.7(3)	B5B C1B C2B B6B	39.1(3)
B10 B5 C1 B4	-39.6(3)	B4B C1B C2B B6B	105.8(3)
B6 B5 C1 B4	-140.7(3)	B3B C1B C2B B6B	145.2(3)
B11 B5 C1 B4	-102.0(3)	C13B C1B C2B B8B	147.0(3)
B10 B5 C1 B6	101.1(3)	B5B C1B C2B B8B	-67.2(3)
B4 B5 C1 B6	140.7(3)	B4B C1B C2B B8B	-0.4(4)
B11 B5 C1 B6	38.7(3)	B3B C1B C2B B8B	39.0(3)
B10 B5 C1 C2	63.3(3)	B6B C1B C2B B8B	-106.2(3)

B4 B5 C1 C2	102.9(3)	C13B C1B C2B B3B	108.0(4)
B6 B5 C1 C2	-37.8(2)	B5B C1B C2B B3B	-106.2(3)
B11 B5 C1 C2	0.9(4)	B4B C1B C2B B3B	-39.4(3)
B10 B5 C1 B3	-1.7(3)	B6B C1B C2B B3B	-145.2(3)
B4 B5 C1 B3	38.0(3)	B5B C1B C13B C14B	-47.5(5)
B6 B5 C1 B3	-102.8(3)	B4B C1B C13B C14B	-125.5(4)
B11 B5 C1 B3	-64.1(3)	B3B C1B C13B C14B	160.3(4)
C2 B3 C1 C13	-107.4(3)	B6B C1B C13B C14B	25.9(5)
B8 B3 C1 C13	-143.7(3)	C2B C1B C13B C14B	92.5(4)
B9 B3 C1 C13	153.5(3)	B5B C1B C13B C18B	134.9(4)
B4 B3 C1 C13	114.8(3)	B4B C1B C13B C18B	56.9(6)
C2 B3 C1 B4	137.8(2)	B3B C1B C13B C18B	-17.2(5)
B8 B3 C1 B4	101.5(3)	B6B C1B C13B C18B	-151.6(4)
B9 B3 C1 B4	38.7(2)	C2B C1B C13B C18B	-85.1(5)
C2 B3 C1 B6	32.2(2)	B5B C1B C13B Cr6	42.5(6)
B8 B3 C1 B6	-4.0(3)	B4B C1B C13B Cr6	-35.5(6)
B9 B3 C1 B6	-66.8(3)	B3B C1B C13B Cr6	-109.7(4)
B4 B3 C1 B6	-105.5(3)	B6B C1B C13B Cr6	115.9(4)
C2 B3 C1 B5	99.9(2)	C2B C1B C13B Cr6	-177.5(3)
B8 B3 C1 B5	63.7(3)	C26B Cr6 C13B C14B	-59.8(12)
B9 B3 C1 B5	0.8(3)	C25B Cr6 C13B C14B	68.1(4)
B4 B3 C1 B5	-37.9(2)	C27B Cr6 C13B C14B	159.8(4)
B8 B3 C1 C2	-36.2(2)	C15B Cr6 C13B C14B	-31.0(5)
B9 B3 C1 C2	-99.1(3)	C18B Cr6 C13B C14B	-131.0(5)
B4 B3 C1 C2	-137.8(2)	C16B Cr6 C13B C14B	-65.4(6)
B11 B7 C2 C19	-141.4(3)	C17B Cr6 C13B C14B	-102.8(5)
B6 B7 C2 C19	-103.7(3)	C26B Cr6 C13B C18B	71.3(12)
B8 B7 C2 C19	115.6(3)	C25B Cr6 C13B C18B	-160.8(4)
B12 B7 C2 C19	155.1(3)	C27B Cr6 C13B C18B	-69.2(5)
B11 B7 C2 B8	102.9(3)	C14B Cr6 C13B C18B	131.0(5)
B6 B7 C2 B8	140.7(3)	C15B Cr6 C13B C18B	100.1(5)
B12 B7 C2 B8	39.4(3)	C16B Cr6 C13B C18B	65.7(6)
B11 B7 C2 B3	65.1(3)	C17B Cr6 C13B C18B	28.3(6)
B6 B7 C2 B3	102.8(3)	C26B Cr6 C13B C1B	-173.2(10)
B8 B7 C2 B3	-37.9(3)	C25B Cr6 C13B C1B	-45.3(5)
B12 B7 C2 B3	1.6(4)	C27B Cr6 C13B C1B	46.4(5)
B11 B7 C2 C1	-0.1(3)	C14B Cr6 C13B C1B	-113.4(6)
B6 B7 C2 C1	37.6(2)	C15B Cr6 C13B C1B	-144.4(6)
B8 B7 C2 C1	-103.0(3)	C18B Cr6 C13B C1B	115.6(6)
B12 B7 C2 C1	-63.6(3)	C16B Cr6 C13B C1B	-178.8(7)
B11 B7 C2 B6	-37.8(3)	C17B Cr6 C13B C1B	143.9(6)
B8 B7 C2 B6	-140.7(3)	C18BC13BC14BC15B	3.7(7)
B12 B7 C2 B6	-101.2(3)	C1B C13B C14B C15B	-173.9(4)
B9 B8 C2 C19	146.8(3)	Cr6 C13B C14B C15B	56.9(5)
B3 B8 C2 C19	107.7(3)	C18B C13B C14B Cr6	-53.2(4)
B7 B8 C2 C19	-111.8(3)	C1B C13B C14B Cr6	129.1(3)
B12 B8 C2 C19	-150.3(3)	C26B Cr6 C14B C13B	160.3(4)
B9 B8 C2 B7	-101.4(3)	C25B Cr6 C14B C13B	-115.0(3)
B3 B8 C2 B7	-140.5(3)	C27B Cr6 C14B C13B	-29.6(6)

B12 B8 C2 B7	-38.5(3)	C15B Cr6 C14B C13B	129.8(7)
B9 B8 C2 B3	39.1(2)	C18B Cr6 C14B C13B	29.7(3)
B7 B8 C2 B3	140.5(3)	C16B Cr6 C14B C13B	103.2(6)
B12 B8 C2 B3	101.9(3)	C17B Cr6 C14B C13B	66.2(4)
B9 B8 C2 C1	1.1(3)	C26B Cr6 C14B C15B	30.5(7)
B3 B8 C2 C1	-38.0(2)	C25B Cr6 C14B C15B	115.2(6)
B7 B8 C2 C1	102.5(2)	C27B Cr6 C14B C15B	-159.4(6)
B12 B8 C2 C1	64.0(3)	C18B Cr6 C14B C15B	-100.1(6)
B9 B8 C2 B6	-63.5(3)	C16B Cr6 C14B C15B	-26.6(7)
B3 B8 C2 B6	-102.6(3)	C17B Cr6 C14B C15B	-63.6(6)
B7 B8 C2 B6	37.9(3)	C13B Cr6 C14B C15B	-129.8(7)
B12 B8 C2 B6	-0.6(3)	C13BC14BC15BC16B	-3.7(10)
C1 B3 C2 C19	105.4(3)	Cr6 C14B C15B C16B	54.1(7)
B8 B3 C2 C19	-116.3(3)	C13B C14B C15B Cr6	-57.8(4)
B9 B3 C2 C19	-155.7(3)	C26B Cr6 C15B C16B	68.2(6)
B4 B3 C2 C19	141.7(3)	C25B Cr6 C15B C16B	157.9(6)
C1 B3 C2 B7	-100.2(3)	C27B Cr6 C15B C16B	-56.1(16)
B8 B3 C2 B7	38.0(3)	C14B Cr6 C15B C16B	-134.0(9)
B9 B3 C2 B7	-1.3(4)	C18B Cr6 C15B C16B	-66.2(6)
B4 B3 C2 B7	-64.0(3)	C17B Cr6 C15B C16B	-29.4(5)
C1 B3 C2 B8	-138.3(2)	C13B Cr6 C15B C16B	-103.7(6)
B9 B3 C2 B8	-39.4(3)	C26B Cr6 C15B C14B	-157.8(5)
B4 B3 C2 B8	-102.0(3)	C25B Cr6 C15B C14B	-68.1(5)
B8 B3 C2 C1	138.3(2)	C27B Cr6 C15B C14B	77.9(17)
B9 B3 C2 C1	98.9(3)	C18B Cr6 C15B C14B	67.8(5)
B4 B3 C2 C1	36.2(2)	C16B Cr6 C15B C14B	134.0(9)
C1 B3 C2 B6	-31.9(2)	C17B Cr6 C15B C14B	104.6(5)
B8 B3 C2 B6	106.4(3)	C13B Cr6 C15B C14B	30.3(4)
B9 B3 C2 B6	67.0(3)	C14BC15BC16BC17B	1.3(13)
B4 B3 C2 B6	4.3(3)	Cr6 C15B C16B C17B	54.1(8)
C13 C1 C2 C19	-5.9(4)	C14B C15B C16B Cr6	-52.8(6)
B4 C1 C2 C19	-149.5(3)	C26B Cr6 C16B C15B	-112.5(7)
B6 C1 C2 C19	104.8(3)	C25B Cr6 C16B C15B	-31.0(7)
B5 C1 C2 C19	143.3(3)	C27B Cr6 C16B C15B	162.9(5)
B3 C1 C2 C19	-110.0(3)	C14B Cr6 C16B C15B	29.0(5)
C13 C1 C2 B7	-149.7(3)	C18B Cr6 C16B C15B	102.8(5)
B4 C1 C2 B7	66.7(3)	C17B Cr6 C16B C15B	132.8(8)
B6 C1 C2 B7	-39.0(2)	C13B Cr6 C16B C15B	65.9(5)
B5 C1 C2 B7	-0.5(3)	C26B Cr6 C16B C17B	114.7(7)
B3 C1 C2 B7	106.2(3)	C25B Cr6 C16B C17B	-163.8(5)
C13 C1 C2 B8	143.0(3)	C27B Cr6 C16B C17B	30.1(7)
B4 C1 C2 B8	-0.7(3)	C14B Cr6 C16B C17B	-103.8(5)
B6 C1 C2 B8	-106.3(3)	C15B Cr6 C16B C17B	-132.8(8)
B5 C1 C2 B8	-67.8(3)	C18B Cr6 C16B C17B	-30.0(5)
B3 C1 C2 B8	38.9(2)	C13B Cr6 C16B C17B	-66.9(5)
C13 C1 C2 B3	104.1(3)	C15BC16BC17BC18B	0.8(13)
B4 C1 C2 B3	-39.6(2)	Cr6 C16B C17B C18B	55.2(6)
B6 C1 C2 B3	-145.2(2)	C15B C16B C17B Cr6	-54.5(8)
B5 C1 C2 B3	-106.7(3)	C26B Cr6 C17B C18B	163.0(6)

C13 C1 C2 B6	-110.7(3)	C25B Cr6 C17B C18B	-70.5(18)
B4 C1 C2 B6	105.6(3)	C27B Cr6 C17B C18B	70.7(7)
B5 C1 C2 B6	38.5(3)	C14B Cr6 C17B C18B	-65.4(6)
B3 C1 C2 B6	145.2(2)	C15B Cr6 C17B C18B	-102.8(6)
C1 B6 C2 C19	-107.1(2)	C16B Cr6 C17B C18B	-130.2(10)
B11 B6 C2 C19	152.6(3)	C13B Cr6 C17B C18B	-28.5(5)
B5 B6 C2 C19	-143.9(2)	C26B Cr6 C17B C16B	-66.7(7)
B7 B6 C2 C19	114.7(3)	C25B Cr6 C17B C16B	59.7(15)
C1 B6 C2 B7	138.2(3)	C27B Cr6 C17B C16B	-159.1(6)
B11 B6 C2 B7	37.8(2)	C14B Cr6 C17B C16B	64.9(6)
B5 B6 C2 B7	101.3(3)	C15B Cr6 C17B C16B	27.4(5)
C1 B6 C2 B8	100.2(3)	C18B Cr6 C17B C16B	130.2(10)
B11 B6 C2 B8	-0.1(3)	C13B Cr6 C17B C16B	101.8(7)
B5 B6 C2 B8	63.3(3)	C16BC17BC18BC13B	-0.5(11)
B7 B6 C2 B8	-38.0(3)	Cr6 C17B C18B C13B	54.7(6)
C1 B6 C2 B3	32.1(2)	C16B C17B C18B Cr6	-55.2(6)
B11 B6 C2 B3	-68.2(3)	C14BC13BC18BC17B	-1.7(8)
B5 B6 C2 B3	-4.7(3)	C1B C13B C18B C17B	175.9(5)
B7 B6 C2 B3	-106.1(3)	Cr6 C13B C18B C17B	-54.4(6)
B11 B6 C2 C1	-100.4(3)	C14B C13B C18B Cr6	52.7(4)
B5 B6 C2 C1	-36.9(2)	C1B C13B C18B Cr6	-129.7(3)
B7 B6 C2 C1	-138.2(3)	C26B Cr6 C18B C17B	-24.0(9)
B4 C1 C13 C14	-156.0(3)	C25B Cr6 C18B C17B	161.4(7)
B6 C1 C13 C14	-4.2(4)	C27B Cr6 C18B C17B	-112.4(8)
B5 C1 C13 C14	-78.9(4)	C14B Cr6 C18B C17B	103.6(7)
C2 C1 C13 C14	65.0(4)	C15B Cr6 C18B C17B	65.1(7)
B3 C1 C13 C14	132.0(3)	C16B Cr6 C18B C17B	30.4(8)
B4 C1 C13 C18	26.5(4)	C13B Cr6 C18B C17B	133.4(8)
B6 C1 C13 C18	178.3(3)	C26B Cr6 C18B C13B	-157.3(5)
B5 C1 C13 C18	103.6(4)	C25B Cr6 C18B C13B	28.0(6)
C2 C1 C13 C18	-112.5(3)	C27B Cr6 C18B C13B	114.2(4)
B3 C1 C13 C18	-45.5(4)	C14B Cr6 C18B C13B	-29.8(3)
B4 C1 C13 Cr1	-63.3(4)	C15B Cr6 C18B C13B	-68.3(4)
B6 C1 C13 Cr1	88.5(3)	C16B Cr6 C18B C13B	-102.9(6)
B5 C1 C13 Cr1	13.8(5)	C17B Cr6 C18B C13B	-133.4(8)
C2 C1 C13 Cr1	157.7(2)	B7B C2B C19B C24B	76.1(5)
B3 C1 C13 Cr1	-135.3(3)	B6B C2B C19B C24B	0.3(5)
C25 Cr1 C13 C14	63.2(3)	B8B C2B C19B C24B	153.1(4)
C26 Cr1 C13 C14	-89.3(7)	B3B C2B C19B C24B	-134.7(3)
C27 Cr1 C13 C14	154.0(2)	C1B C2B C19B C24B	-68.2(4)
C16 Cr1 C13 C14	-65.5(2)	B7B C2B C19B C20B	-105.6(4)
C18 Cr1 C13 C14	-131.3(3)	B6B C2B C19B C20B	178.6(3)
C15 Cr1 C13 C14	-29.6(2)	B8B C2B C19B C20B	-28.5(5)
C17 Cr1 C13 C14	-101.5(2)	B3B C2B C19B C20B	43.7(4)
C25 Cr1 C13 C18	-165.5(2)	C1B C2B C19B C20B	110.1(3)
C26 Cr1 C13 C18	42.0(7)	B7B C2B C19B Cr5	-16.5(5)
C27 Cr1 C13 C18	-74.7(3)	B6B C2B C19B Cr5	-92.3(4)
C16 Cr1 C13 C18	65.9(2)	B8B C2B C19B Cr5	60.6(5)
C14 Cr1 C13 C18	131.3(3)	B3B C2B C19B Cr5	132.7(3)

C15 Cr1 C13 C18	101.7(2)	C1B C2B C19B Cr5	-160.8(2)
C17 Cr1 C13 C18	29.9(2)	C30B Cr5 C19B C24B	-57.0(3)
C25 Cr1 C13 C1	-53.3(3)	C28B Cr5 C19B C24B	-146.9(3)
C26 Cr1 C13 C1	154.2(6)	C29B Cr5 C19B C24B	106.4(5)
C27 Cr1 C13 C1	37.5(3)	C20B Cr5 C19B C24B	132.4(3)
C16 Cr1 C13 C1	178.0(3)	C23B Cr5 C19B C24B	29.7(3)
C14 Cr1 C13 C1	-116.5(4)	C22B Cr5 C19B C24B	66.1(3)
C18 Cr1 C13 C1	112.1(4)	C21B Cr5 C19B C24B	103.1(3)
C15 Cr1 C13 C1	-146.1(3)	C30B Cr5 C19B C20B	170.6(3)
C17 Cr1 C13 C1	142.0(3)	C28B Cr5 C19B C20B	80.7(3)
C18 C13 C14 C15	0.8(5)	C29B Cr5 C19B C20B	-25.9(6)
C1 C13 C14 C15	-176.7(3)	C24B Cr5 C19B C20B	-132.4(3)
Cr1 C13 C14 C15	55.4(3)	C23B Cr5 C19B C20B	-102.7(3)
C18 C13 C14 Cr1	-54.6(3)	C22B Cr5 C19B C20B	-66.3(3)
C1 C13 C14 Cr1	127.9(3)	C21B Cr5 C19B C20B	-29.2(3)
C25 Cr1 C14 C13	-120.4(2)	C30B Cr5 C19B C2B	59.1(4)
C26 Cr1 C14 C13	155.3(2)	C28B Cr5 C19B C2B	-30.8(4)
C27 Cr1 C14 C13	-35.4(3)	C29B Cr5 C19B C2B	-137.5(5)
C16 Cr1 C14 C13	103.7(2)	C24B Cr5 C19B C2B	116.1(4)
C18 Cr1 C14 C13	29.99(18)	C20B Cr5 C19B C2B	-111.5(4)
C15 Cr1 C14 C13	131.9(3)	C23B Cr5 C19B C2B	145.8(4)
C17 Cr1 C14 C13	67.4(2)	C22B Cr5 C19B C2B	-177.8(4)
C25 Cr1 C14 C15	107.7(3)	C21B Cr5 C19B C2B	-140.8(4)
C26 Cr1 C14 C15	23.4(4)	C24BC19BC20BC21B	2.7(6)
C27 Cr1 C14 C15	-167.3(3)	C2B C19B C20B C21B	-175.7(4)
C16 Cr1 C14 C15	-28.2(3)	Cr5 C19B C20B C21B	55.5(4)
C13 Cr1 C14 C15	-131.9(3)	C24B C19B C20B Cr5	-52.8(3)
C18 Cr1 C14 C15	-101.9(3)	C2B C19B C20B Cr5	128.8(3)
C17 Cr1 C14 C15	-64.6(3)	C30B Cr5 C20B C21B	-147.3(4)
C13 C14 C15 C16	-2.7(5)	C28B Cr5 C20B C21B	125.4(3)
Cr1 C14 C15 C16	52.7(3)	C29B Cr5 C20B C21B	38.5(4)
C13 C14 C15 Cr1	-55.4(3)	C24B Cr5 C20B C21B	-102.9(3)
C25 Cr1 C15 C16	153.0(3)	C23B Cr5 C20B C21B	-65.4(3)
C26 Cr1 C15 C16	62.3(3)	C22B Cr5 C20B C21B	-29.1(3)
C27 Cr1 C15 C16	-88.9(8)	C19B Cr5 C20B C21B	-131.5(4)
C14 Cr1 C15 C16	-132.9(4)	C30B Cr5 C20B C19B	-15.7(5)
C13 Cr1 C15 C16	-104.0(3)	C28B Cr5 C20B C19B	-103.0(3)
C18 Cr1 C15 C16	-66.3(2)	C29B Cr5 C20B C19B	170.1(2)
C17 Cr1 C15 C16	-29.5(2)	C24B Cr5 C20B C19B	28.6(2)
C25 Cr1 C15 C14	-74.1(3)	C23B Cr5 C20B C19B	66.1(2)
C26 Cr1 C15 C14	-164.8(2)	C22B Cr5 C20B C19B	102.4(3)
C27 Cr1 C15 C14	44.0(9)	C21B Cr5 C20B C19B	131.5(4)
C16 Cr1 C15 C14	132.9(4)	C19BC20BC21BC22B	-1.5(7)
C13 Cr1 C15 C14	28.9(2)	Cr5 C20B C21B C22B	54.1(4)
C18 Cr1 C15 C14	66.6(2)	C19B C20B C21B Cr5	-55.6(4)
C17 Cr1 C15 C14	103.5(3)	C30B Cr5 C21B C22B	-22.9(8)
C14 C15 C16 C17	4.3(6)	C28B Cr5 C21B C22B	166.3(3)
Cr1 C15 C16 C17	56.7(4)	C29B Cr5 C21B C22B	78.5(3)
C14 C15 C16 Cr1	-52.5(3)	C24B Cr5 C21B C22B	-66.2(3)

C25 Cr1 C16 C15	-35.9(3)	C20B Cr5 C21B C22B	-132.3(4)
C26 Cr1 C16 C15	-120.9(3)	C23B Cr5 C21B C22B	-28.8(3)
C27 Cr1 C16 C15	157.9(3)	C19B Cr5 C21B C22B	-102.5(3)
C14 Cr1 C16 C15	29.0(2)	C30B Cr5 C21B C20B	109.4(7)
C13 Cr1 C16 C15	65.3(2)	C28B Cr5 C21B C20B	-61.4(3)
C18 Cr1 C16 C15	102.6(2)	C29B Cr5 C21B C20B	-149.2(3)
C17 Cr1 C16 C15	131.1(3)	C24B Cr5 C21B C20B	66.1(3)
C25 Cr1 C16 C17	-167.0(2)	C23B Cr5 C21B C20B	103.5(3)
C26 Cr1 C16 C17	107.9(2)	C22B Cr5 C21B C20B	132.3(4)
C27 Cr1 C16 C17	26.8(4)	C19B Cr5 C21B C20B	29.8(2)
C14 Cr1 C16 C17	-102.1(2)	C20BC21BC22BC23B	0.2(7)
C13 Cr1 C16 C17	-65.9(2)	Cr5 C21B C22B C23B	54.2(4)
C18 Cr1 C16 C17	-28.5(2)	C20B C21B C22B Cr5	-54.0(4)
C15 Cr1 C16 C17	-131.1(3)	C30B Cr5 C22B C21B	171.1(3)
C15 C16 C17 C18	-3.9(6)	C28B Cr5 C22B C21B	-24.1(5)
Cr1 C16 C17 C18	52.9(3)	C29B Cr5 C22B C21B	-101.6(3)
C15 C16 C17 Cr1	-56.8(4)	C24B Cr5 C22B C21B	102.6(3)
C25 Cr1 C17 C16	38.0(7)	C20B Cr5 C22B C21B	28.9(3)
C26 Cr1 C17 C16	-73.3(3)	C23B Cr5 C22B C21B	132.3(4)
C27 Cr1 C17 C16	-162.8(2)	C19B Cr5 C22B C21B	66.3(3)
C14 Cr1 C17 C16	66.7(2)	C30B Cr5 C22B C23B	38.8(4)
C13 Cr1 C17 C16	103.5(2)	C28B Cr5 C22B C23B	-156.3(3)
C18 Cr1 C17 C16	133.6(3)	C29B Cr5 C22B C23B	126.1(3)
C15 Cr1 C17 C16	29.5(2)	C24B Cr5 C22B C23B	-29.6(3)
C25 Cr1 C17 C18	-95.6(7)	C20B Cr5 C22B C23B	-103.4(3)
C26 Cr1 C17 C18	153.1(3)	C21B Cr5 C22B C23B	-132.3(4)
C27 Cr1 C17 C18	63.6(3)	C19B Cr5 C22B C23B	-66.0(3)
C16 Cr1 C17 C18	-133.6(3)	C21BC22BC23BC24B	0.0(7)
C14 Cr1 C17 C18	-66.9(2)	Cr5 C22B C23B C24B	54.1(4)
C13 Cr1 C17 C18	-30.1(2)	C21B C22B C23B Cr5	-54.2(4)
C15 Cr1 C17 C18	-104.1(2)	C30B Cr5 C23B C22B	-148.9(3)
C16 C17 C18 C13	2.0(5)	C28B Cr5 C23B C22B	117.0(10)
Cr1 C17 C18 C13	54.8(3)	C29B Cr5 C23B C22B	-57.7(3)
C16 C17 C18 Cr1	-52.8(3)	C24B Cr5 C23B C22B	131.9(4)
C14 C13 C18 C17	-0.4(5)	C20B Cr5 C23B C22B	65.7(3)
C1 C13 C18 C17	177.2(3)	C21B Cr5 C23B C22B	29.2(3)
Cr1 C13 C18 C17	-55.0(3)	C19B Cr5 C23B C22B	103.0(3)
C14 C13 C18 Cr1	54.6(3)	C30B Cr5 C23B C24B	79.2(3)
C1 C13 C18 Cr1	-127.8(3)	C28B Cr5 C23B C24B	-15.0(11)
C25 Cr1 C18 C17	153.6(3)	C29B Cr5 C23B C24B	170.4(3)
C26 Cr1 C18 C17	-35.7(3)	C20B Cr5 C23B C24B	-66.2(3)
C27 Cr1 C18 C17	-121.1(3)	C22B Cr5 C23B C24B	-131.9(4)
C16 Cr1 C18 C17	27.9(2)	C21B Cr5 C23B C24B	-102.7(3)
C14 Cr1 C18 C17	101.6(3)	C19B Cr5 C23B C24B	-28.9(2)
C13 Cr1 C18 C17	130.8(3)	C20BC19BC24BC23B	-2.5(6)
C15 Cr1 C18 C17	64.1(2)	C2B C19B C24B C23B	175.9(4)
C25 Cr1 C18 C13	22.7(4)	Cr5 C19B C24B C23B	-55.1(3)
C26 Cr1 C18 C13	-166.6(2)	C20B C19B C24B Cr5	52.6(3)
C27 Cr1 C18 C13	108.1(2)	C2B C19B C24B Cr5	-129.0(3)

C16 Cr1 C18 C13	-103.0(2)	C22BC23BC24BC19B	1.2(7)
C14 Cr1 C18 C13	-29.23(18)	Cr5 C23B C24B C19B	55.8(3)
C15 Cr1 C18 C13	-66.7(2)	C22B C23B C24B Cr5	-54.6(4)
C17 Cr1 C18 C13	-130.8(3)	C30B Cr5 C24B C19B	127.1(3)
B7 C2 C19 C20	-147.7(3)	C28B Cr5 C24B C19B	43.6(3)
B8 C2 C19 C20	-70.5(4)	C29B Cr5 C24B C19B	-147.8(3)
B3 C2 C19 C20	4.4(4)	C20B Cr5 C24B C19B	-29.3(2)
C1 C2 C19 C20	73.3(4)	C23B Cr5 C24B C19B	-131.8(4)
B6 C2 C19 C20	140.2(3)	C22B Cr5 C24B C19B	-103.0(3)
B7 C2 C19 C24	33.3(4)	C21B Cr5 C24B C19B	-65.9(2)
B8 C2 C19 C24	110.5(4)	C30B Cr5 C24B C23B	-101.1(3)
B3 C2 C19 C24	-174.6(3)	C28B Cr5 C24B C23B	175.4(3)
C1 C2 C19 C24	-105.7(3)	C29B Cr5 C24B C23B	-15.9(5)
B6 C2 C19 C24	-38.8(4)	C20B Cr5 C24B C23B	102.5(3)
B7 C2 C19 Cr2	-56.0(4)	C22B Cr5 C24B C23B	28.9(3)
B8 C2 C19 Cr2	21.2(4)	C21B Cr5 C24B C23B	65.9(3)
B3 C2 C19 Cr2	96.1(3)	C19B Cr5 C24B C23B	131.8(4)
C1 C2 C19 Cr2	165.0(2)	C26B Cr6 C25B O1B	-151(12)
B6 C2 C19 Cr2	-128.1(3)	C27B Cr6 C25B O1B	-61(12)
C28 Cr2 C19 C20	62.5(3)	C14B Cr6 C25B O1B	76(12)
C29 Cr2 C19 C20	-99.6(6)	C15B Cr6 C25B O1B	111(12)
C30 Cr2 C19 C20	151.2(3)	C18B Cr6 C25B O1B	25(12)
C24 Cr2 C19 C20	-130.7(3)	C16B Cr6 C25B O1B	128(12)
C21 Cr2 C19 C20	-28.6(3)	C17B Cr6 C25B O1B	81(12)
C23 Cr2 C19 C20	-101.8(3)	C13B Cr6 C25B O1B	42(12)
C22 Cr2 C19 C20	-65.5(3)	C25B Cr6 C26B O2B	152(21)
C28 Cr2 C19 C24	-166.7(3)	C27B Cr6 C26B O2B	63(21)
C29 Cr2 C19 C24	31.2(6)	C14B Cr6 C26B O2B	-124(21)
C30 Cr2 C19 C24	-78.1(3)	C15B Cr6 C26B O2B	-105(21)
C20 Cr2 C19 C24	130.7(3)	C18B Cr6 C26B O2B	-25(22)
C21 Cr2 C19 C24	102.1(3)	C16B Cr6 C26B O2B	-73(21)
C23 Cr2 C19 C24	28.9(2)	C17B Cr6 C26B O2B	-39(22)
C22 Cr2 C19 C24	65.2(2)	C13B Cr6 C26B O2B	-79(22)
C28 Cr2 C19 C2	-53.4(4)	C26B Cr6 C27B O3B	110(9)
C29 Cr2 C19 C2	144.5(5)	C25B Cr6 C27B O3B	22(9)
C30 Cr2 C19 C2	35.2(3)	C14B Cr6 C27B O3B	-62(9)
C24 Cr2 C19 C2	113.3(3)	C15B Cr6 C27B O3B	-125(8)
C20 Cr2 C19 C2	-115.9(4)	C18B Cr6 C27B O3B	-115(9)
C21 Cr2 C19 C2	-144.6(3)	C16B Cr6 C27B O3B	-167(9)
C23 Cr2 C19 C2	142.3(3)	C17B Cr6 C27B O3B	-149(9)
C22 Cr2 C19 C2	178.6(3)	C13B Cr6 C27B O3B	-80(9)
C24 C19 C20 C21	1.0(5)	C30B Cr5 C28B O4B	39(19)
C2 C19 C20 C21	-178.0(3)	C29B Cr5 C28B O4B	-51(19)
Cr2 C19 C20 C21	54.6(3)	C24B Cr5 C28B O4B	122(19)
C24 C19 C20 Cr2	-53.6(3)	C20B Cr5 C28B O4B	-176(100)
C2 C19 C20 Cr2	127.4(3)	C23B Cr5 C28B O4B	134(19)
C28 Cr2 C20 C21	105.2(3)	C22B Cr5 C28B O4B	-129(19)
C29 Cr2 C20 C21	19.4(5)	C21B Cr5 C28B O4B	-144(19)
C30 Cr2 C20 C21	-171.0(3)	C19B Cr5 C28B O4B	147(19)

C24 Cr2 C20 C21	-102.9(3)	C30B Cr5 C29B O5B	-168(100)
C23 Cr2 C20 C21	-65.9(3)	C28B Cr5 C29B O5B	-81(19)
C22 Cr2 C20 C21	-29.6(3)	C24B Cr5 C29B O5B	108(19)
C19 Cr2 C20 C21	-132.8(4)	C20B Cr5 C29B O5B	9(19)
C28 Cr2 C20 C19	-122.0(3)	C23B Cr5 C29B O5B	98(19)
C29 Cr2 C20 C19	152.3(3)	C22B Cr5 C29B O5B	67(19)
C30 Cr2 C20 C19	-38.1(4)	C21B Cr5 C29B O5B	31(19)
C24 Cr2 C20 C19	30.0(2)	C19B Cr5 C29B O5B	28(19)
C21 Cr2 C20 C19	132.8(4)	C28B Cr5 C30B O6B	158(17)
C23 Cr2 C20 C19	67.0(2)	C29B Cr5 C30B O6B	-115(17)
C22 Cr2 C20 C19	103.2(3)	C24B Cr5 C30B O6B	29(17)
C19 C20 C21 C22	-0.2(6)	C20B Cr5 C30B O6B	69(17)
Cr2 C20 C21 C22	54.4(4)	C23B Cr5 C30B O6B	-8(17)
C19 C20 C21 Cr2	-54.7(3)	C22B Cr5 C30B O6B	-30(17)
C28 Cr2 C21 C22	152.1(3)	C21B Cr5 C30B O6B	-13(17)
C29 Cr2 C21 C22	61.0(3)	C19B Cr5 C30B O6B	59(17)
C30 Cr2 C21 C22	-103.3(8)	O6A C30A Cr7 C28A	-17(27)
C24 Cr2 C21 C22	-65.3(3)	O6A C30A Cr7 C29A	74(27)
C20 Cr2 C21 C22	-131.2(4)	O6A C30A Cr7 C24A	-151(27)
C23 Cr2 C21 C22	-28.4(3)	O6A C30A Cr7 C22A	153(27)
C19 Cr2 C21 C22	-102.4(3)	O6A C30A Cr7 C23A	173(100)
C28 Cr2 C21 C20	-76.7(3)	O6A C30A Cr7 C20A	-99(27)
C29 Cr2 C21 C20	-167.7(3)	O6A C30A Cr7 C21A	-166(26)
C30 Cr2 C21 C20	28.0(10)	O6A C30A Cr7 C19A	-117(27)
C24 Cr2 C21 C20	65.9(3)	O4A C28A Cr7 C30A	-36(12)
C23 Cr2 C21 C20	102.8(3)	O4A C28A Cr7 C29A	-123(12)
C22 Cr2 C21 C20	131.2(4)	O4A C28A Cr7 C24A	50(12)
C19 Cr2 C21 C20	28.8(2)	O4A C28A Cr7 C22A	155(12)
C20 C21 C22 C23	0.0(7)	O4A C28A Cr7 C23A	101(12)
Cr2 C21 C22 C23	54.4(4)	O4A C28A Cr7 C20A	104(12)
C20 C21 C22 Cr2	-54.4(4)	O4A C28A Cr7 C21A	138(12)
C28 Cr2 C22 C23	-169.8(3)	O4A C28A Cr7 C19A	69(12)
C29 Cr2 C22 C23	104.3(3)	O5A C29A Cr7 C30A	82(11)
C30 Cr2 C22 C23	22.0(4)	O5A C29A Cr7 C28A	169(11)
C24 Cr2 C22 C23	-29.5(2)	O5A C29A Cr7 C24A	-4(11)
C20 Cr2 C22 C23	-103.2(3)	O5A C29A Cr7 C22A	-55(11)
C21 Cr2 C22 C23	-132.8(4)	O5A C29A Cr7 C23A	-21(11)
C19 Cr2 C22 C23	-66.4(2)	O5A C29A Cr7 C20A	-104(11)
C28 Cr2 C22 C21	-36.9(4)	O5A C29A Cr7 C21A	-88(11)
C29 Cr2 C22 C21	-122.8(3)	O5A C29A Cr7 C19A	-57(11)
C30 Cr2 C22 C21	154.8(3)	C19A C24A Cr7 C30A	116.7(3)
C24 Cr2 C22 C21	103.4(3)	C23A C24A Cr7 C30A	-112.7(4)
C20 Cr2 C22 C21	29.6(2)	C19A C24A Cr7 C28A	31.9(4)
C23 Cr2 C22 C21	132.8(4)	C23A C24A Cr7 C28A	162.5(4)
C19 Cr2 C22 C21	66.5(3)	C19A C24A Cr7 C29A	-158.0(3)
C21 C22 C23 C24	-0.5(6)	C23A C24A Cr7 C29A	-27.4(5)
Cr2 C22 C23 C24	53.8(3)	C19A C24A Cr7 C22A	-102.0(3)
C21 C22 C23 Cr2	-54.4(4)	C23A C24A Cr7 C22A	28.6(4)
C28 Cr2 C23 C22	30.6(9)	C19A C24A Cr7 C23A	-130.6(5)

C29 Cr2 C23 C22	-76.6(3)	C19A C24A Cr7 C20A	-29.8(2)
C30 Cr2 C23 C22	-166.5(3)	C23A C24A Cr7 C20A	100.8(4)
C24 Cr2 C23 C22	131.8(4)	C19A C24A Cr7 C21A	-66.2(3)
C20 Cr2 C23 C22	65.6(2)	C23A C24A Cr7 C21A	64.4(4)
C21 Cr2 C23 C22	28.8(2)	C23A C24A Cr7 C19A	130.6(5)
C19 Cr2 C23 C22	102.8(3)	C23A C22A Cr7 C30A	34.4(5)
C28 Cr2 C23 C24	-101.2(8)	C21A C22A Cr7 C30A	167.4(3)
C29 Cr2 C23 C24	151.6(3)	C23A C22A Cr7 C28A	-161.1(3)
C30 Cr2 C23 C24	61.7(3)	C21A C22A Cr7 C28A	-28.1(4)
C20 Cr2 C23 C24	-66.2(2)	C23A C22A Cr7 C29A	114.4(4)
C21 Cr2 C23 C24	-103.0(3)	C21A C22A Cr7 C29A	-112.7(3)
C22 Cr2 C23 C24	-131.8(4)	C23A C22A Cr7 C24A	-29.6(3)
C19 Cr2 C23 C24	-29.0(2)	C21A C22A Cr7 C24A	103.3(3)
C22 C23 C24 C19	1.4(6)	C21A C22A Cr7 C23A	133.0(5)
Cr2 C23 C24 C19	55.7(3)	C23A C22A Cr7 C20A	-103.3(4)
C22 C23 C24 Cr2	-54.3(3)	C21A C22A Cr7 C20A	29.6(3)
C20 C19 C24 C23	-1.6(5)	C23A C22A Cr7 C21A	-133.0(5)
C2 C19 C24 C23	177.4(3)	C23A C22A Cr7 C19A	-66.2(3)
Cr2 C19 C24 C23	-55.5(3)	C21A C22A Cr7 C19A	66.7(3)
C20 C19 C24 Cr2	53.9(3)	C22A C23A Cr7 C30A	-156.2(4)
C2 C19 C24 Cr2	-127.0(3)	C24A C23A Cr7 C30A	71.5(4)
C28 Cr2 C24 C23	153.9(4)	C22A C23A Cr7 C28A	68.5(10)
C29 Cr2 C24 C23	-36.7(3)	C24A C23A Cr7 C28A	-63.8(11)
C30 Cr2 C24 C23	-123.4(3)	C22A C23A Cr7 C29A	-66.9(4)
C20 Cr2 C24 C23	102.5(3)	C24A C23A Cr7 C29A	160.8(4)
C21 Cr2 C24 C23	65.7(3)	C22A C23A Cr7 C24A	132.3(5)
C22 Cr2 C24 C23	29.0(3)	C24A C23A Cr7 C22A	-132.3(5)
C19 Cr2 C24 C23	132.4(3)	C22A C23A Cr7 C20A	65.1(4)
C28 Cr2 C24 C19	21.4(4)	C24A C23A Cr7 C20A	-67.3(3)
C29 Cr2 C24 C19	-169.1(2)	C22A C23A Cr7 C21A	28.2(4)
C30 Cr2 C24 C19	104.1(2)	C24A C23A Cr7 C21A	-104.1(4)
C20 Cr2 C24 C19	-29.93(19)	C22A C23A Cr7 C19A	102.8(4)
C21 Cr2 C24 C19	-66.7(2)	C24A C23A Cr7 C19A	-29.5(3)
C23 Cr2 C24 C19	-132.4(3)	C21A C20A Cr7 C30A	-162.6(4)
C22 Cr2 C24 C19	-103.5(2)	C19A C20A Cr7 C30A	-29.5(4)
C26 Cr1 C25 O1	61(13)	C21A C20A Cr7 C28A	115.3(3)
C27 Cr1 C25 O1	148(13)	C19A C20A Cr7 C28A	-111.7(3)
C16 Cr1 C25 O1	-23(13)	C21A C20A Cr7 C29A	27.0(4)
C14 Cr1 C25 O1	-80(13)	C19A C20A Cr7 C29A	160.1(2)
C13 Cr1 C25 O1	-112(13)	C21A C20A Cr7 C24A	-104.0(3)
C18 Cr1 C25 O1	-126(13)	C19A C20A Cr7 C24A	29.1(2)
C15 Cr1 C25 O1	-44(13)	C21A C20A Cr7 C22A	-29.0(3)
C17 Cr1 C25 O1	-52(13)	C19A C20A Cr7 C22A	104.0(3)
C25 Cr1 C26 O2	-134(24)	C21A C20A Cr7 C23A	-65.6(3)
C27 Cr1 C26 O2	138(24)	C19A C20A Cr7 C23A	67.5(3)
C16 Cr1 C26 O2	-4(24)	C19A C20A Cr7 C21A	133.1(4)
C14 Cr1 C26 O2	-50(24)	C21A C20A Cr7 C19A	-133.1(4)
C13 Cr1 C26 O2	20(25)	C22A C21A Cr7 C30A	-50.9(14)
C18 Cr1 C26 O2	52(24)	C20A C21A Cr7 C30A	80.5(14)

C15 Cr1 C26 O2	-35(24)	C22A C21A Cr7 C28A	160.5(3)
C17 Cr1 C26 O2	31(24)	C20A C21A Cr7 C28A	-68.0(3)
C25 Cr1 C27 O3	-19(19)	C22A C21A Cr7 C29A	68.2(3)
C26 Cr1 C27 O3	70(19)	C20A C21A Cr7 C29A	-160.3(3)
C16 Cr1 C27 O3	151(19)	C22A C21A Cr7 C24A	-66.3(3)
C14 Cr1 C27 O3	-103(19)	C20A C21A Cr7 C24A	65.2(3)
C13 Cr1 C27 O3	-124(19)	C20A C21A Cr7 C22A	131.5(4)
C18 Cr1 C27 O3	-160(19)	C22A C21A Cr7 C23A	-28.7(3)
C15 Cr1 C27 O3	-138(19)	C20A C21A Cr7 C23A	102.8(3)
C17 Cr1 C27 O3	167(19)	C22A C21A Cr7 C20A	-131.5(4)
C29 Cr2 C28 O4	-99(13)	C22A C21A Cr7 C19A	-102.6(3)
C30 Cr2 C28 O4	-11(13)	C20A C21A Cr7 C19A	28.8(2)
C24 Cr2 C28 O4	73(13)	C24A C19A Cr7 C30A	-67.7(3)
C20 Cr2 C28 O4	119(13)	C20A C19A Cr7 C30A	160.7(3)
C21 Cr2 C28 O4	155(13)	C2A C19A Cr7 C30A	48.9(4)
C23 Cr2 C28 O4	153(12)	C24A C19A Cr7 C28A	-157.3(3)
C22 Cr2 C28 O4	176(100)	C20A C19A Cr7 C28A	71.1(3)
C19 Cr2 C28 O4	86(13)	C2A C19A Cr7 C28A	-40.7(4)
C28 Cr2 C29 O5	79(18)	C24A C19A Cr7 C29A	69.3(8)
C30 Cr2 C29 O5	-7(18)	C20A C19A Cr7 C29A	-62.3(7)
C24 Cr2 C29 O5	-94(18)	C2A C19A Cr7 C29A	-174.1(6)
C20 Cr2 C29 O5	165(18)	C20A C19A Cr7 C24A	-131.6(4)
C21 Cr2 C29 O5	177(100)	C2A C19A Cr7 C24A	116.5(5)
C23 Cr2 C29 O5	-116(18)	C24A C19A Cr7 C22A	67.2(3)
C22 Cr2 C29 O5	-151(18)	C20A C19A Cr7 C22A	-64.4(3)
C19 Cr2 C29 O5	-118(18)	C2A C19A Cr7 C22A	-176.2(4)
C28 Cr2 C30 O6	-19(11)	C24A C19A Cr7 C23A	30.7(3)
C29 Cr2 C30 O6	70(11)	C20A C19A Cr7 C23A	-100.9(3)
C24 Cr2 C30 O6	-162(11)	C2A C19A Cr7 C23A	147.2(4)
C20 Cr2 C30 O6	-104(11)	C24A C19A Cr7 C20A	131.6(4)
C21 Cr2 C30 O6	-125(11)	C2A C19A Cr7 C20A	-111.8(4)
C23 Cr2 C30 O6	166(11)	C24A C19A Cr7 C21A	103.2(3)
C22 Cr2 C30 O6	152(11)	C20A C19A Cr7 C21A	-28.4(3)
C19 Cr2 C30 O6	-126(11)	C2A C19A Cr7 C21A	-140.3(4)
C2A B3A B4A C1A	-37.3(2)	C2C B3C B4C C1C	-37.3(2)
B9A B3A B4A C1A	-135.8(3)	B8C B3C B4C C1C	-98.8(3)
B8A B3A B4A C1A	-97.9(3)	B9C B3C B4C C1C	-136.5(3)
C1A B3A B4A B10A	98.4(4)	C2C B3C B4C B9C	99.1(3)
C2A B3A B4A B10A	61.1(4)	C1C B3C B4C B9C	136.5(3)
B9A B3A B4A B10A	-37.4(4)	B8C B3C B4C B9C	37.7(3)
B8A B3A B4A B10A	0.6(4)	C2C B3C B4C B10C	60.1(4)
C1A B3A B4A B5A	35.4(3)	C1C B3C B4C B10C	97.5(3)
C2A B3A B4A B5A	-2.0(3)	B8C B3C B4C B10C	-1.3(4)
B9A B3A B4A B5A	-100.4(3)	B9C B3C B4C B10C	-39.0(3)
B8A B3A B4A B5A	-62.5(4)	C2C B3C B4C B5C	-2.4(4)
C1A B3A B4A B9A	135.8(3)	C1C B3C B4C B5C	34.9(2)
C2A B3A B4A B9A	98.5(3)	B8C B3C B4C B5C	-63.9(3)
B8A B3A B4A B9A	37.9(3)	B9C B3C B4C B5C	-101.6(3)
B3A B4A B5A C1A	-35.8(2)	B9C B4C B5C C1C	-98.5(3)

B10A B4A B5A C1A	-136.6(3)	B10C B4C B5C C1C	-136.4(3)
B9A B4A B5A C1A	-98.4(3)	B3C B4C B5C C1C	-35.6(2)
C1A B4A B5A B6A	34.9(2)	C1C B4C B5C B11C	99.7(3)
B3A B4A B5A B6A	-0.9(4)	B9C B4C B5C B11C	1.2(4)
B10A B4A B5A B6A	-101.7(3)	B10C B4C B5C B11C	-36.7(3)
B9A B4A B5A B6A	-63.5(4)	B3C B4C B5C B11C	64.1(3)
C1A B4A B5A B10A	136.6(3)	C1C B4C B5C B10C	136.4(3)
B3A B4A B5A B10A	100.8(3)	B9C B4C B5C B10C	38.0(3)
B9A B4A B5A B10A	38.2(3)	B3C B4C B5C B10C	100.8(3)
C1A B4A B5A B11A	98.5(3)	C1C B4C B5C B6C	36.0(3)
B3A B4A B5A B11A	62.7(4)	B9C B4C B5C B6C	-62.4(4)
B10AB4AB5AB11A	-38.1(3)	B10C B4C B5C B6C	-100.4(3)
B9A B4A B5A B11A	0.1(4)	B3C B4C B5C B6C	0.4(4)
C1A B5A B6A C2A	37.7(2)	C1C B5C B6C C2C	37.2(2)
B4A B5A B6A C2A	3.5(4)	B11C B5C B6C C2C	-99.0(3)
B10A B5A B6A C2A	-60.2(4)	B10C B5C B6C C2C	-62.0(3)
B11A B5A B6A C2A	-98.1(3)	B4C B5C B6C C2C	1.8(4)
B4A B5A B6A C1A	-34.3(2)	B11C B5C B6C C1C	-136.2(3)
B10A B5A B6A C1A	-98.0(3)	B10C B5C B6C C1C	-99.2(3)
B11A B5A B6A C1A	-135.8(3)	B4C B5C B6C C1C	-35.4(2)
C1A B5A B6A B7A	99.0(3)	C1C B5C B6C B11C	136.2(3)
B4A B5A B6A B7A	64.8(3)	B10C B5C B6C B11C	37.1(3)
B10A B5A B6A B7A	1.1(4)	B4C B5C B6C B11C	100.8(3)
B11A B5A B6A B7A	-36.8(3)	C1C B5C B6C B7C	99.1(3)
C1A B5A B6A B11A	135.8(3)	B11C B5C B6C B7C	-37.1(3)
B4A B5A B6A B11A	101.5(3)	B10C B5C B6C B7C	0.0(4)
B10AB5AB6AB11A	37.9(3)	B4C B5C B6C B7C	63.7(3)
C1A B6A B7A C2A	-38.3(3)	C1C B6C B7C C2C	-37.3(2)
B5A B6A B7A C2A	-99.3(3)	B11C B6C B7C C2C	-133.9(3)
B11A B6A B7A C2A	-136.3(4)	B5C B6C B7C C2C	-97.7(3)
C2A B6A B7A B11A	136.3(4)	C2C B6C B7C B12C	96.6(3)
C1A B6A B7A B11A	98.0(4)	C1C B6C B7C B12C	59.3(3)
B5A B6A B7A B11A	37.0(3)	B11C B6C B7C B12C	-37.3(3)
C2A B6A B7A B12A	98.5(4)	B5C B6C B7C B12C	-1.1(3)
C1A B6A B7A B12A	60.2(4)	C2C B6C B7C B8C	34.4(3)
B5A B6A B7A B12A	-0.8(4)	C1C B6C B7C B8C	-2.9(4)
B11AB6AB7AB12A	-37.8(3)	B11C B6C B7C B8C	-99.5(3)
C2A B6A B7A B8A	34.5(3)	B5C B6C B7C B8C	-63.3(3)
C1A B6A B7A B8A	-3.7(4)	C2C B6C B7C B11C	133.9(3)
B5A B6A B7A B8A	-64.7(4)	C1C B6C B7C B11C	96.6(3)
B11A B6A B7A B8A	-101.7(4)	B5C B6C B7C B11C	36.2(3)
C1A B3A B8A C2A	37.2(3)	C1C B3C B8C C2C	36.8(2)
B9A B3A B8A C2A	136.3(3)	B9C B3C B8C C2C	136.0(3)
B4A B3A B8A C2A	98.3(3)	B4C B3C B8C C2C	98.2(3)
C1A B3A B8A B9A	-99.1(3)	C2C B3C B8C B12C	-98.3(3)
C2A B3A B8A B9A	-136.3(3)	C1C B3C B8C B12C	-61.5(3)
B4A B3A B8A B9A	-38.0(3)	B9C B3C B8C B12C	37.7(3)
C1A B3A B8A B7A	1.7(4)	B4C B3C B8C B12C	-0.1(4)
C2A B3A B8A B7A	-35.5(3)	C2C B3C B8C B7C	-34.3(3)

B9A B3A B8A B7A	100.8(4)	C1C B3C B8C B7C	2.5(4)
B4A B3A B8A B7A	62.8(4)	B9C B3C B8C B7C	101.7(3)
C1A B3A B8A B12A	-61.7(4)	B4C B3C B8C B7C	63.9(4)
C2A B3A B8A B12A	-98.9(4)	C2C B3C B8C B9C	-136.0(3)
B9A B3A B8A B12A	37.4(3)	C1C B3C B8C B9C	-99.2(3)
B4A B3A B8A B12A	-0.6(4)	B4C B3C B8C B9C	-37.7(3)
B11A B7A B8A C2A	-97.7(3)	B6C B7C B8C C2C	-34.6(3)
B12A B7A B8A C2A	-136.1(3)	B12C B7C B8C C2C	-134.8(3)
B6A B7A B8A C2A	-34.6(3)	B11C B7C B8C C2C	-97.2(3)
C2A B7A B8A B9A	98.8(3)	C2C B7C B8C B3C	34.8(3)
B11A B7A B8A B9A	1.2(4)	B6C B7C B8C B3C	0.3(4)
B12A B7A B8A B9A	-37.2(3)	B12C B7C B8C B3C	-99.9(3)
B6A B7A B8A B9A	64.2(4)	B11C B7C B8C B3C	-62.4(4)
C2A B7A B8A B3A	35.9(3)	C2C B7C B8C B12C	134.8(3)
B11A B7A B8A B3A	-61.8(4)	B6C B7C B8C B12C	100.2(3)
B12A B7A B8A B3A	-100.2(4)	B11C B7C B8C B12C	37.6(3)
B6A B7A B8A B3A	1.3(4)	C2C B7C B8C B9C	97.5(3)
C2A B7A B8A B12A	136.1(3)	B6C B7C B8C B9C	62.9(4)
B11AB7AB8AB12A	38.4(3)	B12C B7C B8C B9C	-37.3(3)
B6A B7A B8A B12A	101.4(4)	B11C B7C B8C B9C	0.3(4)
C1A B3A B9A B8A	100.0(3)	C1C B4C B9C B3C	38.4(2)
C2A B3A B9A B8A	37.1(3)	B10C B4C B9C B3C	136.8(3)
B4A B3A B9A B8A	137.9(3)	B5C B4C B9C B3C	99.7(3)
C1A B3A B9A B10A	-0.6(4)	C1C B4C B9C B12C	-62.6(4)
C2A B3A B9A B10A	-63.5(4)	B10C B4C B9C B12C	35.9(3)
B8A B3A B9A B10A	-100.6(4)	B5C B4C B9C B12C	-1.3(4)
B4A B3A B9A B10A	37.3(3)	B3C B4C B9C B12C	-101.0(3)
C1A B3A B9A B4A	-37.9(3)	C1C B4C B9C B8C	0.8(4)
C2A B3A B9A B4A	-100.8(3)	B10C B4C B9C B8C	99.2(4)
B8A B3A B9A B4A	-137.9(3)	B5C B4C B9C B8C	62.0(4)
C1A B3A B9A B12A	62.9(4)	B3C B4C B9C B8C	-37.6(3)
C2A B3A B9A B12A	0.1(4)	C1C B4C B9C B10C	-98.4(3)
B8A B3A B9A B12A	-37.1(3)	B5C B4C B9C B10C	-37.2(3)
B4A B3A B9A B12A	100.8(4)	B3C B4C B9C B10C	-136.8(3)
C2A B8A B9A B3A	-38.8(3)	C2C B3C B9C B4C	-100.3(3)
B7A B8A B9A B3A	-101.4(3)	C1C B3C B9C B4C	-37.5(3)
B12A B8A B9A B3A	-138.4(3)	B8C B3C B9C B4C	-138.2(3)
C2A B8A B9A B10A	62.1(4)	C2C B3C B9C B12C	0.1(4)
B3A B8A B9A B10A	100.9(3)	C1C B3C B9C B12C	62.8(4)
B7A B8A B9A B10A	-0.5(4)	B8C B3C B9C B12C	-37.9(3)
B12AB8AB9AB10A	-37.5(3)	B4C B3C B9C B12C	100.4(4)
C2A B8A B9A B4A	-1.1(4)	C2C B3C B9C B8C	37.9(3)
B3A B8A B9A B4A	37.7(3)	C1C B3C B9C B8C	100.7(3)
B7A B8A B9A B4A	-63.8(4)	B4C B3C B9C B8C	138.2(3)
B12A B8A B9A B4A	-100.7(4)	C2C B3C B9C B10C	-62.3(4)
C2A B8A B9A B12A	99.6(3)	C1C B3C B9C B10C	0.5(4)
B3A B8A B9A B12A	138.4(3)	B8C B3C B9C B10C	-100.2(3)
B7A B8A B9A B12A	37.0(3)	B4C B3C B9C B10C	38.0(3)
C1A B4A B9A B3A	38.7(3)	C2C B8C B9C B4C	-1.1(4)

B10A B4A B9A B3A	138.2(4)	B3C B8C B9C B4C	37.5(3)
B5A B4A B9A B3A	100.2(3)	B12C B8C B9C B4C	-100.1(4)
C1A B4A B9A B8A	1.0(4)	B7C B8C B9C B4C	-62.4(4)
B3A B4A B9A B8A	-37.8(3)	C2C B8C B9C B3C	-38.6(2)
B10A B4A B9A B8A	100.4(4)	B12C B8C B9C B3C	-137.6(3)
B5A B4A B9A B8A	62.4(4)	B7C B8C B9C B3C	-99.9(3)
C1A B4A B9A B10A	-99.5(4)	C2C B8C B9C B12C	99.0(3)
B3A B4A B9A B10A	-138.2(4)	B3C B8C B9C B12C	137.6(3)
B5A B4A B9A B10A	-38.0(3)	B7C B8C B9C B12C	37.6(3)
C1A B4A B9A B12A	-62.1(4)	C2C B8C B9C B10C	61.5(3)
B3A B4A B9A B12A	-100.9(4)	B3C B8C B9C B10C	100.1(3)
B10AB4AB9AB12A	37.4(4)	B12C B8C B9C B10C	-37.4(3)
B5A B4A B9A B12A	-0.7(4)	B7C B8C B9C B10C	0.2(4)
C1AB4AB10AB12A	62.3(4)	C1C B5C B10C B11C	-100.7(3)
B3AB4AB10AB12A	-0.3(5)	B6C B5C B10C B11C	-37.4(3)
B5AB4AB10AB12A	99.8(4)	B4C B5C B10C B11C	-138.7(3)
B9AB4AB10AB12A	-37.4(4)	C1C B5C B10C B4C	37.9(3)
C1A B4A B10A B9A	99.6(3)	B11C B5C B10C B4C	138.7(3)
B3A B4A B10A B9A	37.0(3)	B6C B5C B10C B4C	101.3(3)
B5A B4A B10A B9A	137.2(4)	C1C B5C B10C B12C	-62.2(4)
C1A B4A B10A B5A	-37.6(3)	B11C B5C B10C B12C	38.6(3)
B3A B4A B10A B5A	-100.1(3)	B6C B5C B10C B12C	1.2(4)
B9A B4A B10A B5A	-137.2(4)	B4C B5C B10C B12C	-100.1(4)
C1AB4AB10AB11A	-0.4(4)	C1C B5C B10C B9C	0.9(4)
B3AB4AB10AB11A	-62.9(4)	B11C B5C B10C B9C	101.7(4)
B5AB4AB10AB11A	37.2(3)	B6C B5C B10C B9C	64.3(4)
B9AB4AB10AB11A	-100.0(4)	B4C B5C B10C B9C	-37.0(3)
B3A B9A B10A B4A	-37.4(3)	C1C B4C B10C B5C	-37.4(3)
B8A B9A B10A B4A	-101.1(3)	B9C B4C B10C B5C	-138.2(3)
B12AB9AB10AB4A	-138.3(4)	B3C B4C B10C B5C	-100.0(3)
B3AB9AB10AB12A	100.9(4)	C1C B4C B10C B11C	-0.2(5)
B8AB9AB10AB12A	37.2(3)	B9C B4C B10C B11C	-101.1(4)
B4AB9AB10AB12A	138.3(4)	B5C B4C B10C B11C	37.1(3)
B3A B9A B10A B5A	0.8(5)	B3C B4C B10C B11C	-62.9(4)
B8A B9A B10A B5A	-62.9(4)	C1C B4C B10C B12C	64.8(4)
B4A B9A B10A B5A	38.1(3)	B9C B4C B10C B12C	-36.0(3)
B12AB9AB10AB5A	-100.2(4)	B5C B4C B10C B12C	102.2(4)
B3AB9AB10AB11A	63.4(4)	B3C B4C B10C B12C	2.2(5)
B8AB9AB10AB11A	-0.3(4)	C1C B4C B10C B9C	100.9(3)
B4AB9AB10AB11A	100.7(3)	B5C B4C B10C B9C	138.2(3)
B12AB9AB10AB11A	-37.6(3)	B3C B4C B10C B9C	38.2(3)
C1A B5A B10A B4A	37.5(3)	B4C B9C B10C B5C	37.4(3)
B6A B5A B10A B4A	100.5(3)	B3C B9C B10C B5C	-0.9(5)
B11A B5A B10A B4A	138.0(4)	B12C B9C B10C B5C	-102.0(4)
C1A B5A B10A B12A	-63.9(4)	B8C B9C B10C B5C	-64.3(4)
B6A B5A B10A B12A	-0.9(5)	B4C B9C B10C B11C	101.1(3)
B4A B5A B10A B12A	-101.4(4)	B3C B9C B10C B11C	62.8(4)
B11AB5AB10AB12A	36.6(3)	B12C B9C B10C B11C	-38.4(3)
C1A B5A B10A B9A	-0.6(5)	B8C B9C B10C B11C	-0.7(4)

B6A B5A B10A B9A	62.4(4)	B3C B9C B10C B4C	-38.3(3)
B4A B5A B10A B9A	-38.1(3)	B12C B9C B10C B4C	-139.5(3)
B11A B5A B10A B9A	99.9(4)	B8C B9C B10C B4C	-101.7(3)
C1A B5A B10A B11A	-100.5(3)	B4C B9C B10C B12C	139.5(3)
B6A B5A B10A B11A	-37.5(3)	B3C B9C B10C B12C	101.2(3)
B4A B5A B10A B11A	-138.0(4)	B8C B9C B10C B12C	37.7(3)
C2A B7A B11A B12A	-100.0(4)	C1C B5C B11C B6C	-39.0(3)
B8A B7A B11A B12A	-38.5(3)	B10C B5C B11C B6C	-138.2(3)
B6A B7A B11A B12A	-137.9(3)	B4C B5C B11C B6C	-101.0(3)
C2A B7A B11A B10A	-62.8(4)	C1C B5C B11C B10C	99.2(3)
B12AB7AB11AB10A	37.2(3)	B6C B5C B11C B10C	138.2(3)
B8A B7A B11A B10A	-1.4(4)	B4C B5C B11C B10C	37.1(3)
B6A B7A B11A B10A	-100.7(3)	C1C B5C B11C B7C	-2.7(4)
C2A B7A B11A B6A	37.9(3)	B10C B5C B11C B7C	-101.8(4)
B12A B7A B11A B6A	137.9(3)	B6C B5C B11C B7C	36.3(3)
B8A B7A B11A B6A	99.4(3)	B4C B5C B11C B7C	-64.7(4)
C2A B7A B11A B5A	0.5(5)	C1C B5C B11C B12C	61.3(4)
B12A B7A B11A B5A	100.5(4)	B10C B5C B11C B12C	-37.8(3)
B8A B7A B11A B5A	62.0(4)	B6C B5C B11C B12C	100.3(3)
B6A B7A B11A B5A	-37.4(3)	B4C B5C B11C B12C	-0.7(4)
B4AB10AB11AB12A	101.0(4)	C2C B6C B11C B5C	99.4(3)
B9AB10AB11AB12A	37.9(3)	C1C B6C B11C B5C	36.8(3)
B5AB10AB11AB12A	138.5(4)	B7C B6C B11C B5C	139.2(3)
B4A B10A B11A B7A	64.1(4)	C2C B6C B11C B10C	61.5(4)
B12AB10AB11AB7A	-36.9(3)	C1C B6C B11C B10C	-1.1(4)
B9A B10A B11A B7A	1.0(4)	B5C B6C B11C B10C	-37.9(3)
B5A B10A B11A B7A	101.6(3)	B7C B6C B11C B10C	101.3(4)
B4A B10A B11A B6A	-0.2(5)	C2C B6C B11C B7C	-39.9(3)
B12AB10AB11AB6A	-101.2(4)	C1C B6C B11C B7C	-102.4(3)
B9A B10A B11A B6A	-63.3(4)	B5C B6C B11C B7C	-139.2(3)
B5A B10A B11A B6A	37.3(3)	C2C B6C B11C B12C	-2.2(4)
B4A B10A B11A B5A	-37.5(3)	C1C B6C B11C B12C	-64.8(4)
B12AB10AB11AB5A	-138.5(4)	B5C B6C B11C B12C	-101.6(4)
B9A B10A B11A B5A	-100.6(3)	B7C B6C B11C B12C	37.6(3)
C2A B6A B11A B12A	0.0(4)	B4C B10C B11C B5C	-37.2(3)
C1A B6A B11A B12A	-63.1(4)	B12C B10C B11C B5C	-137.8(3)
B5A B6A B11A B12A	-100.9(4)	B9C B10C B11C B5C	-100.3(3)
B7A B6A B11A B12A	37.6(3)	B5C B10C B11C B6C	38.1(3)
C2A B6A B11A B7A	-37.5(3)	B4C B10C B11C B6C	0.8(5)
C1A B6A B11A B7A	-100.7(3)	B12C B10C B11C B6C	-99.8(3)
B5A B6A B11A B7A	-138.4(4)	B9C B10C B11C B6C	-62.2(4)
C2A B6A B11A B10A	63.8(4)	B5C B10C B11C B7C	101.2(3)
C1A B6A B11A B10A	0.6(4)	B4C B10C B11C B7C	63.9(4)
B5A B6A B11A B10A	-37.1(3)	B12C B10C B11C B7C	-36.7(3)
B7A B6A B11A B10A	101.3(4)	B9C B10C B11C B7C	0.9(4)
C2A B6A B11A B5A	100.9(3)	B5C B10C B11C B12C	137.8(3)
C1A B6A B11A B5A	37.8(3)	B4C B10C B11C B12C	100.6(4)
B7A B6A B11A B5A	138.4(4)	B9C B10C B11C B12C	37.5(3)
C1A B5A B11A B12A	62.0(5)	C2C B7C B11C B5C	2.6(4)

B6A B5A B11A B12A	101.0(4)	B6C B7C B11C B5C	-37.0(3)
B4A B5A B11A B12A	0.5(5)	B12C B7C B11C B5C	100.9(4)
B10AB5AB11AB12A	-37.3(4)	B8C B7C B11C B5C	63.6(4)
C1A B5A B11A B7A	-1.3(5)	C2C B7C B11C B6C	39.6(3)
B6A B5A B11A B7A	37.7(3)	B12C B7C B11C B6C	137.9(3)
B4A B5A B11A B7A	-62.8(5)	B8C B7C B11C B6C	100.6(3)
B10A B5A B11A B7A	-100.6(4)	C2C B7C B11C B10C	-61.7(4)
C1A B5A B11A B10A	99.3(4)	B6C B7C B11C B10C	-101.3(3)
B6A B5A B11A B10A	138.3(3)	B12C B7C B11C B10C	36.6(3)
B4A B5A B11A B10A	37.8(3)	B8C B7C B11C B10C	-0.7(4)
C1A B5A B11A B6A	-39.0(3)	C2C B7C B11C B12C	-98.3(3)
B4A B5A B11A B6A	-100.5(3)	B6C B7C B11C B12C	-137.9(3)
B10A B5A B11A B6A	-138.3(3)	B8C B7C B11C B12C	-37.3(3)
B10AB11AB12AB7A	-138.7(3)	C2C B8C B12C B9C	-99.7(3)
B6A B11A B12A B7A	-38.1(3)	B3C B8C B12C B9C	-37.3(3)
B5A B11A B12A B7A	-101.7(4)	B7C B8C B12C B9C	-138.5(3)
B7AB11AB12AB10A	138.7(3)	C2C B8C B12C B7C	38.8(3)
B6AB11AB12AB10A	100.6(4)	B3C B8C B12C B7C	101.2(3)
B5AB11AB12AB10A	37.0(3)	B9C B8C B12C B7C	138.5(3)
B7A B11A B12A B8A	37.7(3)	C2C B8C B12C B10C	-60.9(4)
B10AB11AB12AB8A	-101.0(4)	B3C B8C B12C B10C	1.4(4)
B6A B11A B12A B8A	-0.4(5)	B7C B8C B12C B10C	-99.7(4)
B5A B11A B12A B8A	-64.0(5)	B9C B8C B12C B10C	38.8(3)
B7A B11A B12A B9A	100.8(4)	C2C B8C B12C B11C	0.8(4)
B10AB11AB12AB9A	-37.9(3)	B3C B8C B12C B11C	63.2(4)
B6A B11A B12A B9A	62.7(5)	B7C B8C B12C B11C	-38.0(3)
B5A B11A B12A B9A	-0.9(5)	B9C B8C B12C B11C	100.5(4)
C2A B7A B12A B11A	99.3(3)	B4C B9C B12C B8C	101.5(3)
B8A B7A B12A B11A	137.3(3)	B3C B9C B12C B8C	38.1(3)
B6A B7A B12A B11A	37.6(3)	B10C B9C B12C B8C	137.4(3)
C2A B7A B12A B10A	62.0(4)	B4C B9C B12C B7C	64.3(4)
B11AB7AB12AB10A	-37.3(3)	B3C B9C B12C B7C	0.8(5)
B8A B7A B12A B10A	100.1(4)	B8C B9C B12C B7C	-37.2(3)
B6A B7A B12A B10A	0.3(5)	B10C B9C B12C B7C	100.2(4)
C2A B7A B12A B8A	-38.0(3)	B4C B9C B12C B10C	-35.9(3)
B11A B7A B12A B8A	-137.3(3)	B3C B9C B12C B10C	-99.3(3)
B6A B7A B12A B8A	-99.8(3)	B8C B9C B12C B10C	-137.4(3)
C2A B7A B12A B9A	-1.2(4)	B4C B9C B12C B11C	0.9(4)
B11A B7A B12A B9A	-100.5(4)	B3C B9C B12C B11C	-62.6(4)
B8A B7A B12A B9A	36.8(3)	B8C B9C B12C B11C	-100.6(3)
B6A B7A B12A B9A	-63.0(4)	B10C B9C B12C B11C	36.8(3)
B4AB10AB12AB11A	-100.1(4)	C2C B7C B12C B8C	-38.7(3)
B9AB10AB12AB11A	-137.4(3)	B6C B7C B12C B8C	-100.1(3)
B5AB10AB12AB11A	-36.7(3)	B11C B7C B12C B8C	-137.4(3)
B4A B10A B12A B7A	-63.0(5)	C2C B7C B12C B9C	-1.4(4)
B9A B10A B12A B7A	-100.3(4)	B6C B7C B12C B9C	-62.9(4)
B5A B10A B12A B7A	0.4(5)	B8C B7C B12C B9C	37.2(3)
B11AB10AB12AB7A	37.1(3)	B11C B7C B12C B9C	-100.1(4)
B4A B10A B12A B8A	-0.1(5)	C2C B7C B12C B10C	63.3(4)

B9A B10A B12A B8A	-37.3(3)	B6C B7C B12C B10C	1.8(4)
B5A B10A B12A B8A	63.3(5)	B8C B7C B12C B10C	102.0(3)
B11AB10AB12AB8A	100.0(4)	B11C B7C B12C B10C	-35.4(3)
B4A B10A B12A B9A	37.3(3)	C2C B7C B12C B11C	98.7(3)
B5A B10A B12A B9A	100.7(4)	B6C B7C B12C B11C	37.3(3)
B11AB10AB12AB9A	137.4(3)	B8C B7C B12C B11C	137.4(3)
C2A B8A B12A B11A	0.6(5)	B5C B10C B12C B8C	61.7(4)
B9A B8A B12A B11A	100.9(4)	B11C B10C B12C B8C	99.7(3)
B3A B8A B12A B11A	64.0(4)	B4C B10C B12C B8C	-2.3(5)
B7A B8A B12A B11A	-37.9(3)	B9C B10C B12C B8C	-38.3(3)
C2A B8A B12A B7A	38.5(3)	B5C B10C B12C B9C	99.9(3)
B9A B8A B12A B7A	138.8(3)	B11C B10C B12C B9C	137.9(3)
B3A B8A B12A B7A	101.9(3)	B4C B10C B12C B9C	36.0(3)
C2A B8A B12A B10A	-63.0(5)	B5C B10C B12C B7C	-1.9(4)
B9A B8A B12A B10A	37.3(4)	B11C B10C B12C B7C	36.1(3)
B3A B8A B12A B10A	0.4(5)	B4C B10C B12C B7C	-65.8(4)
B7A B8A B12A B10A	-101.5(4)	B9C B10C B12C B7C	-101.8(3)
C2A B8A B12A B9A	-100.3(3)	B5C B10C B12C B11C	-38.0(3)
B3A B8A B12A B9A	-36.9(3)	B4C B10C B12C B11C	-101.9(4)
B7A B8A B12A B9A	-138.8(3)	B9C B10C B12C B11C	-137.9(3)
B3A B9A B12A B11A	-62.5(4)	B5C B11C B12C B8C	-63.5(4)
B8A B9A B12A B11A	-99.9(4)	B6C B11C B12C B8C	0.9(4)
B10AB9AB12AB11A	38.2(3)	B10C B11C B12C B8C	-101.5(3)
B4A B9A B12A B11A	1.0(5)	B7C B11C B12C B8C	38.0(3)
B3A B9A B12A B7A	0.7(4)	B5C B11C B12C B9C	-0.1(4)
B8A B9A B12A B7A	-36.6(3)	B6C B11C B12C B9C	64.3(4)
B10A B9A B12A B7A	101.4(4)	B10C B11C B12C B9C	-38.1(3)
B4A B9A B12A B7A	64.2(4)	B7C B11C B12C B9C	101.4(3)
B3A B9A B12A B10A	-100.7(3)	B5C B11C B12C B7C	-101.5(3)
B8A B9A B12A B10A	-138.1(3)	B6C B11C B12C B7C	-37.2(3)
B4A B9A B12A B10A	-37.2(3)	B10C B11C B12C B7C	-139.5(3)
B3A B9A B12A B8A	37.4(3)	B5C B11C B12C B10C	38.0(3)
B10A B9A B12A B8A	138.1(3)	B6C B11C B12C B10C	102.3(3)
B4A B9A B12A B8A	100.9(3)	B7C B11C B12C B10C	139.5(3)
B3A B4A C1A C13A	-104.9(3)	B11C B5C C1C C13C	143.4(3)
B10A B4A C1A C13A	153.6(3)	B10C B5C C1C C13C	-154.2(3)
B5A B4A C1A C13A	115.5(3)	B6C B5C C1C C13C	104.5(3)
B9A B4A C1A C13A	-143.5(3)	B4C B5C C1C C13C	-115.6(3)
B3A B4A C1A B5A	139.7(3)	B11C B5C C1C B4C	-101.0(4)
B10A B4A C1A B5A	38.1(3)	B10C B5C C1C B4C	-38.7(3)
B9A B4A C1A B5A	101.0(3)	B6C B5C C1C B4C	-140.0(3)
B10A B4A C1A B3A	-101.5(3)	B11C B5C C1C B3C	-63.0(4)
B5A B4A C1A B3A	-139.7(3)	B10C B5C C1C B3C	-0.6(4)
B9A B4A C1A B3A	-38.6(3)	B6C B5C C1C B3C	-102.0(3)
B3A B4A C1A B6A	102.4(3)	B4C B5C C1C B3C	38.0(3)
B10A B4A C1A B6A	0.8(4)	B11C B5C C1C B6C	38.9(3)
B5A B4A C1A B6A	-37.3(3)	B10C B5C C1C B6C	101.3(3)
B9A B4A C1A B6A	63.7(4)	B4C B5C C1C B6C	140.0(3)
B3A B4A C1A C2A	38.2(3)	B11C B5C C1C C2C	1.7(4)

B10A B4A C1A C2A	-63.3(4)	B10C B5C C1C C2C	64.1(4)
B5A B4A C1A C2A	-101.5(3)	B6C B5C C1C C2C	-37.2(2)
B9A B4A C1A C2A	-0.4(4)	B4C B5C C1C C2C	102.7(3)
B6A B5A C1A C13A	105.6(3)	B9C B4C C1C C13C	-144.2(3)
B4A B5A C1A C13A	-113.4(3)	B10C B4C C1C C13C	152.0(3)
B10A B5A C1A C13A	-151.7(3)	B5C B4C C1C C13C	114.1(3)
B11A B5A C1A C13A	144.7(3)	B3C B4C C1C C13C	-106.0(3)
B6A B5A C1A B4A	-141.0(3)	B9C B4C C1C B5C	101.7(3)
B10A B5A C1A B4A	-38.3(3)	B10C B4C C1C B5C	37.9(3)
B11A B5A C1A B4A	-102.0(4)	B3C B4C C1C B5C	140.0(3)
B6A B5A C1A B3A	-102.5(3)	B9C B4C C1C B3C	-38.2(3)
B4A B5A C1A B3A	38.5(3)	B10C B4C C1C B3C	-102.1(3)
B10A B5A C1A B3A	0.2(4)	B5C B4C C1C B3C	-140.0(3)
B11A B5A C1A B3A	-63.5(4)	B9C B4C C1C B6C	63.3(3)
B4A B5A C1A B6A	141.0(3)	B10C B4C C1C B6C	-0.5(4)
B10A B5A C1A B6A	102.7(3)	B5C B4C C1C B6C	-38.4(3)
B11A B5A C1A B6A	39.1(3)	B3C B4C C1C B6C	101.6(3)
B6A B5A C1A C2A	-37.5(3)	B9C B4C C1C C2C	-0.1(3)
B4A B5A C1A C2A	103.5(3)	B10C B4C C1C C2C	-64.0(3)
B10A B5A C1A C2A	65.2(4)	B5C B4C C1C C2C	-101.9(3)
B11A B5A C1A C2A	1.5(4)	B3C B4C C1C C2C	38.1(2)
C2A B3A C1A C13A	-106.4(3)	C2C B3C C1C C13C	-105.3(3)
B9A B3A C1A C13A	154.6(3)	B8C B3C C1C C13C	-141.3(3)
B8A B3A C1A C13A	-142.3(3)	B9C B3C C1C C13C	155.4(3)
B4A B3A C1A C13A	115.6(3)	B4C B3C C1C C13C	116.9(3)
C2A B3A C1A B4A	138.1(3)	C2C B3C C1C B5C	99.4(3)
B9A B3A C1A B4A	39.1(3)	B8C B3C C1C B5C	63.4(3)
B8A B3A C1A B4A	102.1(3)	B9C B3C C1C B5C	0.1(4)
C2A B3A C1A B5A	99.3(3)	B4C B3C C1C B5C	-38.4(3)
B9A B3A C1A B5A	0.3(4)	C2C B3C C1C B4C	137.8(3)
B8A B3A C1A B5A	63.4(4)	B8C B3C C1C B4C	101.8(3)
B4A B3A C1A B5A	-38.8(3)	B9C B3C C1C B4C	38.6(3)
C2A B3A C1A B6A	31.7(3)	C2C B3C C1C B6C	31.6(2)
B9A B3A C1A B6A	-67.3(4)	B8C B3C C1C B6C	-4.4(3)
B8A B3A C1A B6A	-4.2(4)	B9C B3C C1C B6C	-67.7(3)
B4A B3A C1A B6A	-106.3(3)	B4C B3C C1C B6C	-106.3(3)
B9A B3A C1A C2A	-99.0(3)	B8C B3C C1C C2C	-36.0(2)
B8A B3A C1A C2A	-35.9(3)	B9C B3C C1C C2C	-99.3(3)
B4A B3A C1A C2A	-138.1(3)	B4C B3C C1C C2C	-137.8(3)
C2A B6A C1A C13A	105.4(3)	C2C B6C C1C C13C	105.6(3)
B5A B6A C1A C13A	-116.8(3)	B11C B6C C1C C13C	-154.0(3)
B7A B6A C1A C13A	142.6(3)	B5C B6C C1C C13C	-116.4(3)
B11A B6A C1A C13A	-155.4(3)	B7C B6C C1C C13C	142.3(3)
C2A B6A C1A B4A	-100.1(3)	C2C B6C C1C B5C	-138.0(3)
B5A B6A C1A B4A	37.7(3)	B11C B6C C1C B5C	-37.6(3)
B7A B6A C1A B4A	-62.9(4)	B7C B6C C1C B5C	-101.2(3)
B11A B6A C1A B4A	-0.9(4)	C2C B6C C1C B4C	-99.3(3)
C2A B6A C1A B5A	-137.8(3)	B11C B6C C1C B4C	1.0(4)
B7AB6AC1AB5A10	-0.6(3)	B5C B6C C1C B4C	38.6(3)

B11A B6A C1A B5A	-38.6(3)	B7C B6C C1C B4C	-62.6(3)
C2A B6A C1A B3A	-32.2(3)	C2C B6C C1C B3C	-32.1(2)
B5A B6A C1A B3A	105.6(3)	B11C B6C C1C B3C	68.3(3)
B7A B6A C1A B3A	5.0(4)	B5C B6C C1C B3C	105.9(3)
B11A B6A C1A B3A	67.0(4)	B7C B6C C1C B3C	4.6(3)
B5A B6A C1A C2A	137.8(3)	B11C B6C C1C C2C	100.3(3)
B7A B6A C1A C2A	37.2(3)	B5C B6C C1C C2C	138.0(3)
B11A B6A C1A C2A	99.2(3)	B7C B6C C1C C2C	36.7(2)
B9A B8A C2A C19A	141.9(3)	B3C B8C C2C C19C	101.4(3)
B3A B8A C2A C19A	103.6(3)	B12C B8C C2C C19C	-157.6(3)
B7A B8A C2A C19A	-116.0(4)	B7C B8C C2C C19C	-117.6(4)
B12A B8A C2A C19A	-154.7(3)	B9C B8C C2C C19C	139.8(3)
B9A B8A C2A B7A	-102.0(4)	B3C B8C C2C B7C	-140.9(3)
B3A B8A C2A B7A	-140.3(3)	B12C B8C C2C B7C	-40.0(3)
B12A B8A C2A B7A	-38.7(3)	B9C B8C C2C B7C	-102.5(3)
B9A B8A C2A B6A	-64.0(4)	B3C B8C C2C B6C	-103.3(3)
B3A B8A C2A B6A	-102.3(3)	B12C B8C C2C B6C	-2.4(4)
B7A B8A C2A B6A	38.0(3)	B7C B8C C2C B6C	37.6(3)
B12A B8A C2A B6A	-0.6(4)	B9C B8C C2C B6C	-64.9(4)
B9A B8A C2A C1A	0.9(4)	B12C B8C C2C B3C	100.9(3)
B3A B8A C2A C1A	-37.4(3)	B7C B8C C2C B3C	140.9(3)
B7A B8A C2A C1A	102.9(3)	B9C B8C C2C B3C	38.4(3)
B12A B8A C2A C1A	64.2(4)	B3C B8C C2C C1C	-37.3(2)
B9A B8A C2A B3A	38.3(3)	B12C B8C C2C C1C	63.6(3)
B7A B8A C2A B3A	140.3(3)	B7C B8C C2C C1C	103.6(3)
B12A B8A C2A B3A	101.7(4)	B9C B8C C2C C1C	1.0(3)
B11A B7A C2A C19A	-146.9(3)	B6C B7C C2C C19C	-107.6(4)
B12A B7A C2A C19A	150.0(3)	B12C B7C C2C C19C	150.6(3)
B8A B7A C2A C19A	110.9(4)	B8C B7C C2C C19C	111.6(4)
B6A B7A C2A C19A	-108.5(4)	B11C B7C C2C C19C	-147.6(3)
B11A B7A C2A B8A	102.2(4)	B6C B7C C2C B8C	140.7(3)
B12A B7A C2A B8A	39.0(3)	B12C B7C C2C B8C	38.9(3)
B6A B7A C2A B8A	140.5(3)	B11C B7C C2C B8C	100.8(3)
B11A B7A C2A B6A	-38.4(3)	B12C B7C C2C B6C	-101.8(3)
B12A B7A C2A B6A	-101.5(3)	B8C B7C C2C B6C	-140.7(3)
B8A B7A C2A B6A	-140.5(3)	B11C B7C C2C B6C	-40.0(3)
B11A B7A C2A C1A	0.5(4)	B6C B7C C2C B3C	103.4(3)
B12A B7A C2A C1A	-62.6(4)	B12C B7C C2C B3C	1.6(4)
B8A B7A C2A C1A	-101.7(3)	B8C B7C C2C B3C	-37.4(3)
B6A B7A C2A C1A	38.8(3)	B11C B7C C2C B3C	63.4(4)
B11A B7A C2A B3A	64.4(4)	B6C B7C C2C C1C	38.4(3)
B12A B7A C2A B3A	1.3(4)	B12C B7C C2C C1C	-63.4(4)
B8A B7A C2A B3A	-37.7(3)	B8C B7C C2C C1C	-102.3(3)
B6A B7A C2A B3A	102.8(3)	B11C B7C C2C C1C	-1.6(4)
C1A B6A C2A C19A	-105.8(3)	C1C B6C C2C C19C	-103.8(3)
B5A B6A C2A C19A	-142.4(3)	B11C B6C C2C C19C	158.9(3)
B7A B6A C2A C19A	117.1(3)	B5C B6C C2C C19C	-139.6(3)
B11A B6A C2A C19A	155.1(3)	B7C B6C C2C C19C	118.0(4)
C1A B6A C2A B8A	99.5(3)	C1C B6C C2C B8C	100.2(3)

B5A B6A C2A B8A	62.9(3)	B11C B6C C2C B8C	2.9(4)
B7A B6A C2A B8A	-37.7(3)	B5C B6C C2C B8C	64.4(4)
B11A B6A C2A B8A	0.4(4)	B7C B6C C2C B8C	-38.0(3)
C1A B6A C2A B7A	137.1(3)	C1C B6C C2C B7C	138.2(3)
B5A B6A C2A B7A	100.6(3)	B11C B6C C2C B7C	40.9(3)
B11A B6A C2A B7A	38.1(3)	B5C B6C C2C B7C	102.4(3)
B5A B6A C2A C1A	-36.5(2)	C1C B6C C2C B3C	32.3(2)
B7A B6A C2A C1A	-137.1(3)	B11C B6C C2C B3C	-65.0(3)
B11A B6A C2A C1A	-99.1(3)	B5C B6C C2C B3C	-3.5(4)
C1A B6A C2A B3A	31.7(3)	B7C B6C C2C B3C	-105.9(3)
B5A B6A C2A B3A	-4.8(3)	B11C B6C C2C C1C	-97.3(3)
B7A B6A C2A B3A	-105.4(3)	B5C B6C C2C C1C	-35.8(2)
B11A B6A C2A B3A	-67.4(3)	B7C B6C C2C C1C	-138.2(3)
C13A C1A C2A C19A	2.5(4)	C1C B3C C2C C19C	106.1(3)
B4A C1A C2A C19A	-143.6(3)	B8C B3C C2C C19C	-115.2(3)
B5A C1A C2A C19A	149.1(3)	B9C B3C C2C C19C	-154.1(3)
B3A C1A C2A C19A	-104.5(3)	B4C B3C C2C C19C	142.6(3)
B6A C1A C2A C19A	110.3(3)	C1C B3C C2C B8C	-138.6(3)
C13A C1A C2A B8A	145.7(3)	B9C B3C C2C B8C	-38.9(3)
B4A C1A C2A B8A	-0.3(4)	B4C B3C C2C B8C	-102.1(3)
B5A C1A C2A B8A	-67.6(3)	C1C B3C C2C B7C	-100.8(3)
B3A C1A C2A B8A	38.8(3)	B8C B3C C2C B7C	37.8(3)
B6A C1A C2A B8A	-106.4(3)	B9C B3C C2C B7C	-1.1(4)
C13A C1A C2A B7A	-148.0(3)	B4C B3C C2C B7C	-64.3(4)
B4A C1A C2A B7A	66.0(3)	C1C B3C C2C B6C	-32.8(2)
B5A C1A C2A B7A	-1.3(4)	B8C B3C C2C B6C	105.9(3)
B3A C1A C2A B7A	105.0(3)	B9C B3C C2C B6C	67.0(3)
B6A C1A C2A B7A	-40.1(3)	B4C B3C C2C B6C	3.8(4)
C13A C1A C2A B6A	-107.9(3)	B8C B3C C2C C1C	138.6(3)
B4A C1A C2A B6A	106.1(3)	B9C B3C C2C C1C	99.7(3)
B5A C1A C2A B6A	38.8(3)	B4C B3C C2C C1C	36.5(2)
B3A C1A C2A B6A	145.2(3)	C13C C1C C2C C19C	5.0(4)
C13A C1A C2A B3A	107.0(3)	B5C C1C C2C C19C	150.2(3)
B4A C1A C2A B3A	-39.0(3)	B4C C1C C2C C19C	-142.6(3)
B5A C1A C2A B3A	-106.3(3)	B3C C1C C2C C19C	-103.8(3)
B6A C1A C2A B3A	-145.2(3)	B6C C1C C2C C19C	111.3(3)
C1A B3A C2A C19A	107.6(3)	C13C C1C C2C B8C	147.1(3)
B9A B3A C2A C19A	-152.9(3)	B5C C1C C2C B8C	-67.8(3)
B8A B3A C2A C19A	-114.4(3)	B4C C1C C2C B8C	-0.6(3)
B4A B3A C2A C19A	143.9(3)	B3C C1C C2C B8C	38.3(2)
C1A B3A C2A B8A	-138.1(3)	B6C C1C C2C B8C	-106.7(3)
B9A B3A C2A B8A	-38.5(3)	C13C C1C C2C B7C	-145.2(3)
B4A B3A C2A B8A	-101.7(3)	B5C C1C C2C B7C	0.0(4)
C1A B3A C2A B7A	-100.4(3)	B4C C1C C2C B7C	67.1(3)
B9A B3A C2A B7A	-0.9(4)	B3C C1C C2C B7C	106.0(3)
B8A B3A C2A B7A	37.6(3)	B6C C1C C2C B7C	-38.9(3)
B4A B3A C2A B7A	-64.1(3)	C13C C1C C2C B6C	-106.3(3)
C1A B3A C2A B6A	-32.0(2)	B5C C1C C2C B6C	38.9(3)
B9A B3A C2A B6A	67.5(3)	B4C C1C C2C B6C	106.1(3)

B8A B3A C2A B6A	106.0(3)	B3C C1C C2C B6C	145.0(3)
B4A B3A C2A B6A	4.3(3)	C13C C1C C2C B3C	108.8(3)
B9A B3A C2A C1A	99.5(3)	B5C C1C C2C B3C	-106.1(3)
B8A B3A C2A C1A	138.1(3)	B4C C1C C2C B3C	-38.9(3)
B4A B3A C2A C1A	36.3(2)	B6C C1C C2C B3C	-145.0(3)
B4A C1A C13A C14A	-139.1(3)	B5C C1C C13C C14C	-51.2(5)
B5A C1A C13A C14A	-61.0(5)	B4C C1C C13C C14C	-129.8(3)
B3A C1A C13A C14A	148.0(3)	B3C C1C C13C C14C	156.4(3)
B6A C1A C13A C14A	12.7(4)	B6C C1C C13C C14C	22.0(4)
C2A C1A C13A C14A	79.8(4)	C2C C1C C13C C14C	88.1(3)
B4A C1A C13A C18A	38.5(5)	B5C C1C C13C C18C	130.7(3)
B5A C1A C13A C18A	116.6(4)	B4C C1C C13C C18C	52.1(4)
B3A C1A C13A C18A	-34.4(4)	B3C C1C C13C C18C	-21.7(4)
B6A C1A C13A C18A	-169.7(3)	B6C C1C C13C C18C	-156.1(3)
C2A C1A C13A C18A	-102.6(3)	C2C C1C C13C C18C	-90.0(3)
B4A C1A C13A Cr1A	-48.7(4)	B5C C1C C13C Cr2A	39.1(4)
B5A C1A C13A Cr1A	29.4(5)	B4C C1C C13C Cr2A	-39.5(4)
B3A C1A C13A Cr1A	-121.6(3)	B3C C1C C13C Cr2A	-113.3(3)
B6A C1A C13A Cr1A	103.1(3)	B6C C1C C13C Cr2A	112.3(3)
C2A C1A C13A Cr1A	170.2(2)	C2C C1C C13C Cr2A	178.4(2)
C27ACr1AC13AC14A	157.5(3)	C25CCr2AC13C C14C	50.9(3)
C25ACr1AC13AC14A	69.4(3)	C27CCr2AC13C C14C	143.2(3)
C26ACr1AC13AC14A	-62.1(8)	C26CCr2AC13C C14C	-122.7(4)
C18ACr1AC13AC14A	-130.9(3)	C15CCr2AC13C C14C	-29.9(2)
C17ACr1AC13AC14A	-101.8(3)	C18CCr2AC13C C14C	-132.1(3)
C16ACr1AC13AC14A	-65.6(2)	C16CCr2AC13C C14C	-65.5(2)
C15ACr1AC13AC14A	-29.4(2)	C17CCr2AC13C C14C	-102.5(2)
C27ACr1AC13AC18A	-71.6(3)	C25CCr2AC13C C18C	-177.0(3)
C25ACr1AC13AC18A	-159.6(3)	C27CCr2AC13C C18C	-84.7(3)
C26ACr1AC13AC18A	68.9(8)	C26CCr2AC13C C18C	9.3(5)
C14ACr1AC13AC18A	130.9(3)	C14CCr2AC13C C18C	132.1(3)
C17ACr1AC13AC18A	29.1(2)	C15CCr2AC13C C18C	166.6(2)
C16ACr1AC13AC18A	65.4(2)	C16CCr2AC13C C18C	02.2(2)
C15ACr1AC13AC18A	101.5(3)	C17CCr2AC13C C18C	29.6(2)
C27A Cr1A C13A C1A	40.6(3)	C25C Cr2A C13C C1C	-62.3(4)
C25A Cr1A C13A C1A	-47.5(3)	C27C Cr2A C13C C1C	30.0(3)
C26A Cr1A C13A C1A	-178.9(7)	C26C Cr2A C13C C1C	124.0(4)
C14A Cr1A C13A C1A	-116.9(4)	C14C Cr2A C13C C1C	-113.3(3)
C18A Cr1A C13A C1A	112.2(4)	C15C Cr2A C13C C1C	-143.2(3)
C17A Cr1A C13A C1A	141.3(3)	C18C Cr2A C13C C1C	114.7(4)
C16A Cr1A C13A C1A	177.6(3)	C16C Cr2A C13C C1C	-178.7(3)
C15A Cr1A C13A C1A	-146.3(3)	C17C Cr2A C13C C1C	144.2(3)
C18AC13AC14AC15A	3.5(5)	C18CC13CC14CC15C	3.4(5)
C1A C13A C14A C15A	-178.9(3)	C1C C13C C14C C15C	-174.7(3)
Cr1AC13AC14A C15A	56.1(3)	Cr2AC13CC14C C15C	55.8(3)
C18AC13AC14A Cr1A	-52.6(3)	C18CC13CC14C Cr2A	-52.4(3)
C1A C13A C14A Cr1A	125.0(3)	C1C C13C C14C Cr2A	129.5(3)
C27ACr1AC14A C13A	-31.5(4)	C25CCr2AC14C C13C	-134.2(3)
C25ACr1AC14A C13A	-113.9(2)	C27CCr2AC14C C13C	-46.9(3)

C26ACr1AC14A C13A	162.4(3)	C26CCr2AC14C C13C	141.5(3)
C18ACr1AC14A C13A	30.1(2)	C15CCr2AC14C C13C	131.1(3)
C17ACr1AC14A C13A	67.1(2)	C18CCr2AC14C C13C	29.31(18)
C16ACr1AC14A C13A	103.6(2)	C16CCr2AC14C C13C	103.4(2)
C15ACr1AC14A C13A	132.1(3)	C17CCr2AC14C C13C	66.2(2)
C27ACr1AC14A C15A	-163.6(3)	C25CCr2AC14C C15C	94.8(3)
C25ACr1AC14A C15A	114.0(3)	C27CCr2AC14C C15C	-178.0(3)
C26ACr1AC14A C15A	30.3(4)	C26CCr2AC14C C15C	10.4(4)
C18ACr1AC14A C15A	-102.1(3)	C18CCr2AC14C C15C	-101.7(2)
C17ACr1AC14A C15A	-65.0(3)	C16CCr2AC14C C15C	-27.6(2)
C16ACr1AC14A C15A	-28.5(3)	C17CCr2AC14C C15C	-64.8(2)
C13ACr1AC14A C15A	-132.1(3)	C13CCr2AC14C C15C	-131.1(3)
C13AC14AC15AC16A	-3.4(6)	C13CC14CC15CC16C	-2.6(6)
Cr1AC14AC15A C16A	52.9(4)	Cr2AC14CC15C C16C	53.8(4)
C13AC14AC15A Cr1A	-56.3(3)	C13CC14CC15C Cr2A	-56.4(3)
C27ACr1AC15A C16A	-73.4(9)	C25CCr2AC15C C16C	142.0(3)
C25ACr1AC15A C16A	158.0(3)	C27CCr2AC15C C16C	-127.9(8)
C26ACr1AC15A C16A	68.9(3)	C26CCr2AC15C C16C	52.2(3)
C14ACr1AC15A C16A	-132.5(4)	C14CCr2AC15C C16C	-133.5(4)
C18ACr1AC15A C16A	-65.9(2)	C18CCr2AC15C C16C	-66.3(3)
C17ACr1AC15A C16A	-29.2(2)	C17CCr2AC15C C16C	-29.9(2)
C13ACr1AC15A C16A	-103.5(3)	C13CCr2AC15C C16C	-103.7(3)
C27ACr1AC15A C14A	59.1(10)	C25CCr2AC15C C14C	-84.5(3)
C25ACr1AC15A C14A	-69.5(3)	C27CCr2AC15C C14C	5.6(9)
C26ACr1AC15A C14A	-158.6(3)	C26CCr2AC15C C14C	-174.3(2)
C18ACr1AC15A C14A	66.6(2)	C18CCr2AC15C C14C	67.2(2)
C17ACr1AC15A C14A	103.3(3)	C16CCr2AC15C C14C	133.5(4)
C16ACr1AC15A C14A	132.5(4)	C17CCr2AC15C C14C	103.7(2)
C13ACr1AC15A C14A	29.0(2)	C13CCr2AC15C C14C	29.8(2)
C14AC15AC16AC17A	2.8(6)	C14CC15CC16CC17C	1.7(6)
Cr1AC15AC16A C17A	55.0(4)	Cr2AC15CC16C C17C	54.8(4)
C14AC15AC16A Cr1A	-52.3(3)	C14CC15CC16C Cr2A	-53.1(4)
C27ACr1AC16A C15A	160.3(2)	C25CCr2AC16C C15C	-44.3(3)
C25ACr1AC16A C15A	-31.6(3)	C27CCr2AC16C C15C	151.5(4)
C26ACr1AC16A C15A	-113.1(3)	C26CCr2AC16C C15C	-132.8(3)
C14ACr1AC16A C15A	29.3(2)	C14CCr2AC16C C15C	28.8(2)
C18ACr1AC16A C15A	103.2(3)	C18CCr2AC16C C15C	103.1(3)
C17ACr1AC16A C15A	131.8(3)	C17CCr2AC16C C15C	131.5(4)
C13ACr1AC16A C15A	66.0(2)	C13CCr2AC16C C15C	65.8(2)
C27ACr1AC16A C17A	28.5(3)	C25CCr2AC16C C17C	-175.9(3)
C25ACr1AC16A C17A	-163.5(3)	C27CCr2AC16C C17C	20.0(5)
C26ACr1AC16A C17A	115.1(3)	C26CCr2AC16C C17C	95.7(3)
C14ACr1AC16A C17A	-102.5(2)	C14CCr2AC16C C17C	-102.7(3)
C18ACr1AC16A C17A	-28.7(2)	C15CCr2AC16C C17C	-131.5(4)
C15ACr1AC16A C17A	-131.8(3)	C18CCr2AC16C C17C	-28.4(2)
C13ACr1AC16A C17A	-65.8(2)	C13CCr2AC16C C17C	-65.7(2)
C15AC16AC17AC18A	-2.4(7)	C15CC16CC17CC18C	-1.7(6)
Cr1AC16AC17A C18A	53.0(4)	Cr2AC16CC17C C18C	53.3(3)
C15AC16AC17A Cr1A	-55.4(4)	C15CC16CC17C Cr2A	-55.0(4)

C27ACr1AC17A C18A	66.2(3)	C25CCr2AC17C C18C	-124.4(4)
C25ACr1AC17A C18A	-71.1(9)	C27CCr2AC17C C18C	57.2(3)
C26ACr1AC17A C18A	159.6(3)	C26CCr2AC17C C18C	142.5(3)
C14ACr1AC17A C18A	-66.6(2)	C14CCr2AC17C C18C	-67.2(2)
C16ACr1AC17A C18A	-133.1(4)	C15CCr2AC17C C18C	-104.4(3)
C15ACr1AC17A C18A	-104.0(3)	C16CCr2AC17C C18C	-133.2(4)
C13ACr1AC17A C18A	-29.5(2)	C13CCr2AC17C C18C	-30.0(2)
C27ACr1AC17A C16A	-160.7(2)	C25CCr2AC17C C16C	8.8(6)
C25ACr1AC17A C16A	62.1(9)	C27CCr2AC17C C16C	-169.6(3)
C26ACr1AC17A C16A	-67.2(3)	C26CCr2AC17C C16C	-84.3(3)
C14ACr1AC17A C16A	66.5(2)	C14CCr2AC17C C16C	66.0(3)
C18ACr1AC17A C16A	133.1(4)	C15CCr2AC17C C16C	28.8(3)
C15ACr1AC17A C16A	29.1(2)	C18CCr2AC17C C16C	133.2(4)
C13ACr1AC17A C16A	103.6(3)	C13CCr2AC17C C16C	103.2(3)
C16AC17AC18AC13A	2.6(6)	C16CC17CC18CC13C	2.7(6)
Cr1AC17AC18A C13A	56.1(3)	Cr2AC17CC18C C13C	56.0(3)
C16AC17AC18A Cr1A	-53.5(4)	C16CC17CC18C Cr2A	-53.3(4)
C14AC13AC18AC17A	-3.1(5)	C14CC13CC18CC17C	-3.5(5)
C1A C13A C18A C17A	179.2(3)	C1C C13C C18C C17C	174.6(3)
Cr1AC13AC18A C17A	-55.8(3)	Cr2AC13CC18C C17C	-55.7(3)
C14AC13AC18A Cr1A	52.8(3)	C14CC13CC18C Cr2A	52.2(3)
C1A C13A C18A Cr1A	-125.0(3)	C1C C13C C18C Cr2A	-129.7(3)
C27ACr1AC18A C17A	-117.2(3)	C25CCr2AC18C C17C	136.7(5)
C25ACr1AC18A C17A	161.9(4)	C27CCr2AC18C C17C	-130.4(3)
C26ACr1AC18A C17A	-29.2(4)	C26CCr2AC18C C17C	-44.8(3)
C14ACr1AC18A C17A	102.2(3)	C14CCr2AC18C C17C	101.9(3)
C16ACr1AC18A C17A	28.3(3)	C15CCr2AC18C C17C	64.4(3)
C15ACr1AC18A C17A	64.6(3)	C16CCr2AC18C C17C	28.7(3)
C13ACr1AC18A C17A	132.0(4)	C13CCr2AC18C C17C	131.1(3)
C27ACr1AC18A C13A	110.8(3)	C25CCr2AC18C C13C	5.6(6)
C25ACr1AC18A C13A	29.9(4)	C27CCr2AC18C C13C	98.5(3)
C26ACr1AC18A C13A	-161.1(3)	C26CCr2AC18C C13C	-175.9(2)
C14ACr1AC18A C13A	-29.7(2)	C14CCr2AC18C C13C	-29.22(19)
C17ACr1AC18A C13A	-132.0(4)	C15CCr2AC18C C13C	-66.7(2)
C16ACr1AC18A C13A	-103.6(2)	C16CCr2AC18C C13C	-102.4(2)
C15ACr1AC18A C13A	-67.4(2)	C17CCr2AC18C C13C	-131.1(3)
B8A C2A C19A C24A	145.4(4)	B8C C2C C19C C24C	157.3(3)
B7A C2A C19A C24A	69.1(4)	B7C C2C C19C C24C	78.9(5)
B6A C2A C19A C24A	-7.1(5)	B6C C2C C19C C24C	3.5(5)
C1A C2A C19A C24A	-76.3(4)	B3C C2C C19C C24C	-132.1(3)
B3A C2A C19A C24A	-143.0(3)	C1C C2C C19C C24C	-65.8(4)
B8A C2A C19A C20A	-35.7(5)	B8C C2C C19C C20C	-25.3(5)
B7A C2A C19A C20A	-112.0(4)	B7C C2C C19C C20C	-103.7(4)
B6A C2A C19A C20A	171.8(3)	B6C C2C C19C C20C	-179.1(3)
C1A C2A C19A C20A	102.6(3)	B3C C2C C19C C20C	45.4(4)
B3A C2A C19A C20A	36.0(4)	C1C C2C C19C C20C	111.6(3)
B8A C2A C19A Cr7	53.4(5)	B8C C2C C19C Cr8	64.7(4)
B7A C2A C19A Cr7	-23.0(5)	B7C C2C C19C Cr8	-13.7(5)
B6A C2A C19A Cr7	-99.1(3)	B6C C2C C19C Cr8	-89.1(4)

C1A C2A C19A Cr7	-168.3(2)	B3C C2C C19C Cr8	135.3(3)
B3A C2A C19A Cr7	125.0(3)	C1C C2C C19C Cr8	-158.4(2)
C24AC19AC20AC21A	2.0(5)	C30C Cr8 C19C C24C	-67.3(3)
C2A C19A C20A C21A	-177.0(3)	C29C Cr8 C19C C24C	66.3(7)
Cr7 C19A C20A C21A	54.7(3)	C28C Cr8 C19C C24C	-157.9(3)
C24A C19A C20A Cr7	-52.7(3)	C20C Cr8 C19C C24C	132.4(3)
C2A C19A C20A Cr7	128.3(3)	C22C Cr8 C19C C24C	67.1(3)
C19AC20AC21AC22A	-1.1(6)	C23C Cr8 C19C C24C	30.4(3)
Cr7 C20A C21A C22A	53.7(4)	C21C Cr8 C19C C24C	102.7(3)
C19A C20A C21A Cr7	-54.8(3)	C30C Cr8 C19C C20C	160.3(3)
C20AC21AC22AC23A	1.8(7)	C29C Cr8 C19C C20C	-66.1(7)
Cr7 C21A C22A C23A	55.7(4)	C28C Cr8 C19C C20C	69.7(3)
C20A C21A C22A Cr7	-53.9(4)	C24C Cr8 C19C C20C	-132.4(3)
C21AC22AC23AC24A	-3.3(7)	C22C Cr8 C19C C20C	-65.4(3)
Cr7 C22A C23A C24A	52.8(4)	C23C Cr8 C19C C20C	-102.0(3)
C21A C22A C23A Cr7	-56.1(4)	C21C Cr8 C19C C20C	-29.7(3)
C20AC19AC24AC23A	-3.5(6)	C30C Cr8 C19C C2C	48.6(4)
C2A C19A C24A C23A	175.5(3)	C29C Cr8 C19C C2C	-177.7(6)
Cr7 C19A C24A C23A	-56.3(3)	C28C Cr8 C19C C2C	-41.9(4)
C20A C19A C24A Cr7	52.8(3)	C24C Cr8 C19C C2C	115.9(4)
C2A C19A C24A Cr7	-128.3(3)	C20C Cr8 C19C C2C	-111.7(4)
C22AC23AC24AC19A	4.2(7)	C22C Cr8 C19C C2C	-177.0(4)
Cr7 C23A C24A C19A	57.2(3)	C23C Cr8 C19C C2C	146.3(4)
C22A C23A C24A Cr7	-53.0(4)	C21C Cr8 C19C C2C	-141.4(4)
C27A Cr1A C25A O1A	70(26)	C24CC19CC20CC21C	2.7(6)
C26A Cr1A C25A O1A	-21(26)	C2C C19C C20C C21C	-174.8(4)
C14A Cr1A C25A O1A	-156(26)	Cr8 C19C C20C C21C	55.4(4)
C18A Cr1A C25A O1A	151(26)	C24C C19C C20C Cr8	-52.8(3)
C17A Cr1A C25A O1A	-152(25)	C2C C19C C20C Cr8	129.7(3)
C16A Cr1A C25A O1A	-102(26)	C30C Cr8 C20C C21C	-160.4(4)
C15A Cr1A C25A O1A	-121(26)	C29C Cr8 C20C C21C	29.4(4)
C13A Cr1A C25A O1A	169(100)	C28C Cr8 C20C C21C	115.4(3)
C27A Cr1A C26A O2A	-108(10)	C24C Cr8 C20C C21C	-102.2(3)
C25A Cr1A C26A O2A	-22(10)	C22C Cr8 C20C C21C	-28.3(3)
C14A Cr1A C26A O2A	62(10)	C23C Cr8 C20C C21C	-64.5(3)
C18A Cr1A C26A O2A	165(10)	C19C Cr8 C20C C21C	-131.3(4)
C17A Cr1A C26A O2A	148(10)	C30C Cr8 C20C C19C	-29.1(5)
C16A Cr1A C26A O2A	114(10)	C29C Cr8 C20C C19C	160.7(3)
C15A Cr1A C26A O2A	80(10)	C28C Cr8 C20C C19C	-113.2(3)
C13A Cr1A C26A O2A	111(10)	C24C Cr8 C20C C19C	29.2(2)
C25A Cr1A C27A O3A	76(35)	C22C Cr8 C20C C19C	103.1(3)
C26A Cr1A C27A O3A	163(35)	C23C Cr8 C20C C19C	66.8(2)
C14A Cr1A C27A O3A	-7(35)	C21C Cr8 C20C C19C	131.3(4)
C18A Cr1A C27A O3A	-61(35)	C19CC20CC21CC22C	-1.8(7)
C17A Cr1A C27A O3A	-95(35)	Cr8 C20C C21C C22C	53.7(4)
C16A Cr1A C27A O3A	-112(35)	C19C C20C C21C Cr8	-55.4(3)
C15A Cr1A C27A O3A	-54(35)	C30C Cr8 C21C C20C	75.8(12)
C13A Cr1A C27A O3A	-26(35)	C29C Cr8 C21C C20C	-159.3(3)
C2B B3B B4B C1B	-37.7(3)	C28C Cr8 C21C C20C	-67.9(3)

B9B B3B B4B C1B	-135.9(4)	C24C Cr8 C21C C20C	67.0(3)
B8B B3B B4B C1B	-98.2(3)	C22C Cr8 C21C C20C	132.3(4)
C1B B3B B4B B9B	135.9(4)	C23C Cr8 C21C C20C	103.9(3)
C2B B3B B4B B9B	98.1(4)	C19C Cr8 C21C C20C	29.6(2)
B8B B3B B4B B9B	37.7(3)	C30C Cr8 C21C C22C	-56.5(12)
C1B B3B B4B B10B	98.4(3)	C29C Cr8 C21C C22C	68.5(3)
C2B B3B B4B B10B	60.6(4)	C28C Cr8 C21C C22C	159.8(3)
B9B B3B B4B B10B	-37.5(3)	C24C Cr8 C21C C22C	-65.3(3)
B8B B3B B4B B10B	0.2(4)	C20C Cr8 C21C C22C	-132.3(4)
C1B B3B B4B B5B	35.3(3)	C23C Cr8 C21C C22C	-28.3(3)
C2B B3B B4B B5B	-2.4(5)	C19C Cr8 C21C C22C	-102.6(3)
B9B B3B B4B B5B	-100.5(4)	C20CC21CC22CC23C	1.6(7)
B8B B3B B4B B5B	-62.9(4)	Cr8 C21C C22C C23C	54.9(4)
B9B B4B B5B C1B	-98.1(3)	C20C C21C C22C Cr8	-53.3(4)
B10B B4B B5B C1B	-136.2(3)	C30C Cr8 C22C C23C	30.2(4)
B3B B4B B5B C1B	-35.6(3)	C29C Cr8 C22C C23C	112.8(3)
C1B B4B B5B B11B	98.5(3)	C28C Cr8 C22C C23C	-162.5(3)
B9B B4B B5B B11B	0.3(4)	C24C Cr8 C22C C23C	-29.3(3)
B10B B4B B5B B11B	-37.7(3)	C20C Cr8 C22C C23C	-104.1(3)
B3B B4B B5B B11B	62.9(4)	C19C Cr8 C22C C23C	-67.0(3)
C1B B4B B5B B6B	35.2(3)	C21C Cr8 C22C C23C	-133.6(4)
B9B B4B B5B B6B	-63.0(4)	C30C Cr8 C22C C21C	163.8(3)
B10B B4B B5B B6B	-101.0(4)	C29C Cr8 C22C C21C	-113.5(3)
B3B B4B B5B B6B	-0.5(5)	C28C Cr8 C22C C21C	-28.9(4)
C1B B4B B5B B10B	136.2(3)	C24C Cr8 C22C C21C	104.3(3)
B9B B4B B5B B10B	38.0(3)	C20C Cr8 C22C C21C	29.5(3)
B3B B4B B5B B10B	100.6(4)	C23C Cr8 C22C C21C	133.6(4)
C1B B5B B6B C2B	38.1(3)	C19C Cr8 C22C C21C	66.6(3)
B11B B5B B6B C2B	-98.8(3)	C21CC22CC23CC24C	-2.3(7)
B4B B5B B6B C2B	3.2(4)	Cr8 C22C C23C C24C	53.0(4)
B10B B5B B6B C2B	-60.1(4)	C21C C22C C23C Cr8	-55.2(4)
B11B B5B B6B C1B	-136.9(3)	C30C Cr8 C23C C22C	-158.8(3)
B4B B5B B6B C1B	-34.9(3)	C29C Cr8 C23C C22C	-69.2(3)
B10B B5B B6B C1B	-98.2(3)	C28C Cr8 C23C C22C	64.6(9)
C1B B5B B6B B7B	99.6(3)	C24C Cr8 C23C C22C	132.4(4)
B11B B5B B6B B7B	-37.3(3)	C20C Cr8 C23C C22C	64.7(3)
B4B B5B B6B B7B	64.7(4)	C19C Cr8 C23C C22C	102.0(3)
B10B B5B B6B B7B	1.3(4)	C21C Cr8 C23C C22C	27.7(3)
C1B B5B B6B B11B	136.9(3)	C30C Cr8 C23C C24C	68.8(3)
B4B B5B B6B B11B	102.0(4)	C29C Cr8 C23C C24C	158.4(3)
B10B B5B B6B B11B	38.7(3)	C28C Cr8 C23C C24C	-67.8(10)
C1B B6B B7B C2B	-37.9(3)	C20C Cr8 C23C C24C	-67.6(3)
B11B B6B B7B C2B	-136.2(3)	C22C Cr8 C23C C24C	-132.4(4)
B5B B6B B7B C2B	-99.4(3)	C19C Cr8 C23C C24C	-30.4(2)
C2B B6B B7B B11B	136.2(3)	C21C Cr8 C23C C24C	-104.7(3)
C1B B6B B7B B11B	98.3(3)	C22CC23CC24CC19C	3.1(6)
B5B B6B B7B B11B	36.8(3)	Cr8 C23C C24C C19C	56.3(3)
C2B B6B B7B B12B	98.7(4)	C22C C23C C24C Cr8	-53.2(4)
C1B B6B B7B B12B	60.8(4)	C20CC19CC24CC23C	-3.3(6)

B11B B6B B7B B12B	-37.5(4)	C2C C19C C24C C23C	174.1(3)
B5B B6B B7B B12B	-0.7(4)	Cr8 C19C C24C C23C	-56.3(3)
C2B B6B B7B B8B	34.1(3)	C20C C19C C24C Cr8	53.0(3)
C1B B6B B7B B8B	-3.8(4)	C2C C19C C24C Cr8	-129.6(3)
B11B B6B B7B B8B	-102.1(4)	C30C Cr8 C24C C23C	-114.0(4)
B5B B6B B7B B8B	-65.2(4)	C29C Cr8 C24C C23C	-30.5(4)
B11B B7B B8B C2B	-97.7(3)	C28C Cr8 C24C C23C	162.2(3)
B12B B7B B8B C2B	-134.9(3)	C20C Cr8 C24C C23C	101.2(3)
B6B B7B B8B C2B	-34.3(3)	C22C Cr8 C24C C23C	28.7(3)
C2B B7B B8B B12B	134.9(3)	C19C Cr8 C24C C23C	130.2(4)
B11B B7B B8B B12B	37.2(4)	C21C Cr8 C24C C23C	63.9(3)
B6B B7B B8B B12B	100.6(4)	C30C Cr8 C24C C19C	115.8(3)
C2B B7B B8B B3B	35.5(3)	C29C Cr8 C24C C19C	-160.7(2)
B11B B7B B8B B3B	-62.2(4)	C28C Cr8 C24C C19C	31.9(4)
B12B B7B B8B B3B	-99.4(4)	C20C Cr8 C24C C19C	-29.0(2)
B6B B7B B8B B3B	1.3(4)	C22C Cr8 C24C C19C	-101.5(3)
C2B B7B B8B B9B	97.6(4)	C23C Cr8 C24C C19C	-130.2(4)
B11B B7B B8B B9B	-0.1(4)	C21C Cr8 C24C C19C	-66.3(2)
B12B B7B B8B B9B	-37.2(3)	C27C Cr2A C25C O1C	-70(23)
B6B B7B B8B B9B	63.4(4)	C26C Cr2A C25C O1C	-155(23)
C1B B3B B8B C2B	36.9(2)	C14C Cr2A C25C O1C	55(23)
B9B B3B B8B C2B	135.6(3)	C15C Cr2A C25C O1C	93(23)
B4B B3B B8B C2B	98.1(3)	C18C Cr2A C25C O1C	23(23)
C1B B3B B8B B7B	1.7(4)	C16C Cr2A C25C O1C	117(23)
C2B B3B B8B B7B	-35.2(3)	C17C Cr2A C25C O1C	111(23)
B9B B3B B8B B7B	100.4(3)	C13C Cr2A C25C O1C	27(23)
B4B B3B B8B B7B	62.9(4)	C25C Cr2A C26C O2C	115(18)
C1B B3B B8B B12B	-60.9(4)	C27C Cr2A C26C O2C	26(18)
C2B B3B B8B B12B	-97.8(3)	C14C Cr2A C26C O2C	-161(18)
B9B B3B B8B B12B	37.8(3)	C15C Cr2A C26C O2C	-154(18)
B4B B3B B8B B12B	0.3(4)	C18C Cr2A C26C O2C	-64(18)
C1B B3B B8B B9B	-98.7(3)	C16C Cr2A C26C O2C	-126(18)
C2B B3B B8B B9B	-135.6(3)	C17C Cr2A C26C O2C	-89(18)
B4B B3B B8B B9B	-37.5(3)	C13C Cr2A C26C O2C	-70(18)
C1B B3B B9B B10B	-0.4(5)	C25C Cr2A C27C O3C	-19(10)
C2B B3B B9B B10B	-63.3(4)	C26C Cr2A C27C O3C	70(10)
B4B B3B B9B B10B	37.4(4)	C14C Cr2A C27C O3C	-105(10)
B8B B3B B9B B10B	-100.9(4)	C15C Cr2A C27C O3C	-110(9)
C1B B3B B9B B4B	-37.8(3)	C18C Cr2A C27C O3C	-170(10)
C2B B3B B9B B4B	-100.7(3)	C16C Cr2A C27C O3C	147(10)
B8B B3B B9B B4B	-138.4(3)	C17C Cr2A C27C O3C	160(10)
C1B B3B B9B B8B	100.6(3)	C13C Cr2A C27C O3C	-132(10)
C2B B3B B9B B8B	37.6(3)	C30C Cr8 C28C O4C	28(100)
B4B B3B B9B B8B	138.4(3)	C29C Cr8 C28C O4C	-60(100)
C1B B3B B9B B12B	62.6(4)	C24C Cr8 C28C O4C	111(100)
C2B B3B B9B B12B	-0.3(4)	C20C Cr8 C28C O4C	165(100)
B4B B3B B9B B12B	100.4(4)	C22C Cr8 C28C O4C	-143(100)
B8B B3B B9B B12B	-38.0(3)	C23C Cr8 C28C O4C	165(100)
C1B B4B B9B B10B	-99.4(4)	C19C Cr8 C28C O4C	131(100)

B5B B4B B9B B10B	-37.9(3)	C21C Cr8 C28C O4C	-160(100)
B3B B4B B9B B10B	-137.9(3)	C30C Cr8 C29C O5C	-98(33)
C1B B4B B9B B3B	38.5(3)	C28C Cr8 C29C O5C	-10(33)
B10B B4B B9B B3B	137.9(3)	C24C Cr8 C29C O5C	179(100)
B5B B4B B9B B3B	100.0(3)	C20C Cr8 C29C O5C	75(34)
C1B B4B B9B B8B	0.9(5)	C22C Cr8 C29C O5C	126(33)
B10B B4B B9B B8B	100.3(4)	C23C Cr8 C29C O5C	160(33)
B5B B4B B9B B8B	62.4(4)	C19C Cr8 C29C O5C	127(33)
B3B B4B B9B B8B	-37.6(3)	C21C Cr8 C29C O5C	93(33)
C1B B4B B9B B12B	-62.3(4)	C29C Cr8 C30C O6C	-86(20)
B10B B4B B9B B12B	37.1(3)	C28C Cr8 C30C O6C	-175(100)
B5B B4B B9B B12B	-0.8(4)	C24C Cr8 C30C O6C	49(20)
B3B B4B B9B B12B	-100.8(3)	C20C Cr8 C30C O6C	101(20)
C2B B8B B9B B10B	62.1(5)	C22C Cr8 C30C O6C	-4(20)
B7B B8B B9B B10B	0.7(5)	C23C Cr8 C30C O6C	14(20)
B12B B8B B9B B10B	-36.4(3)	C19C Cr8 C30C O6C	83(20)
B3B B8B B9B B10B	100.9(4)	C21C Cr8 C30C O6C	40(20)

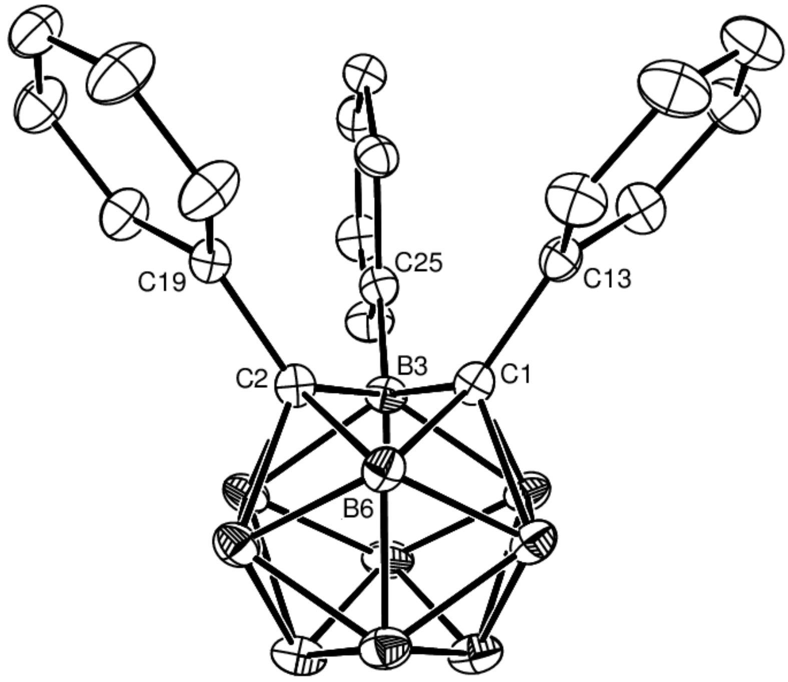

Figure S10. ORTEP drawing (30% probability for thermal ellipsoids) of **Ph3C2B** (the hydrogen atoms are omitted for clarity).

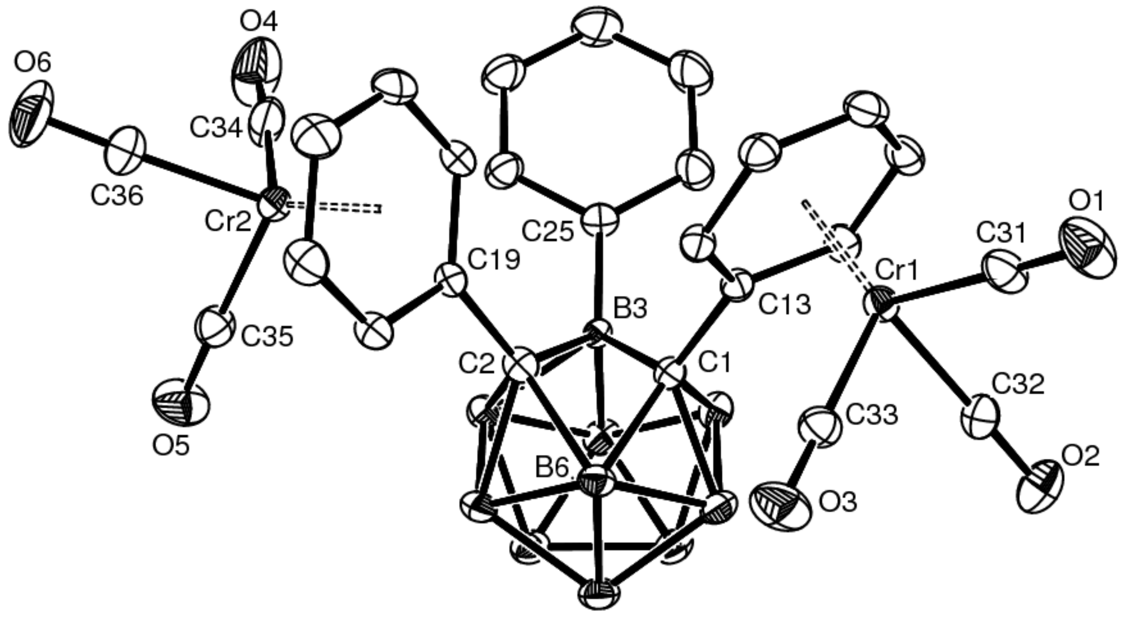

Figure S11. ORTEP drawing (30% probability for thermal ellipsoids) of **Ph3C2BCr2** (the hydrogen atoms are omitted for clarity).

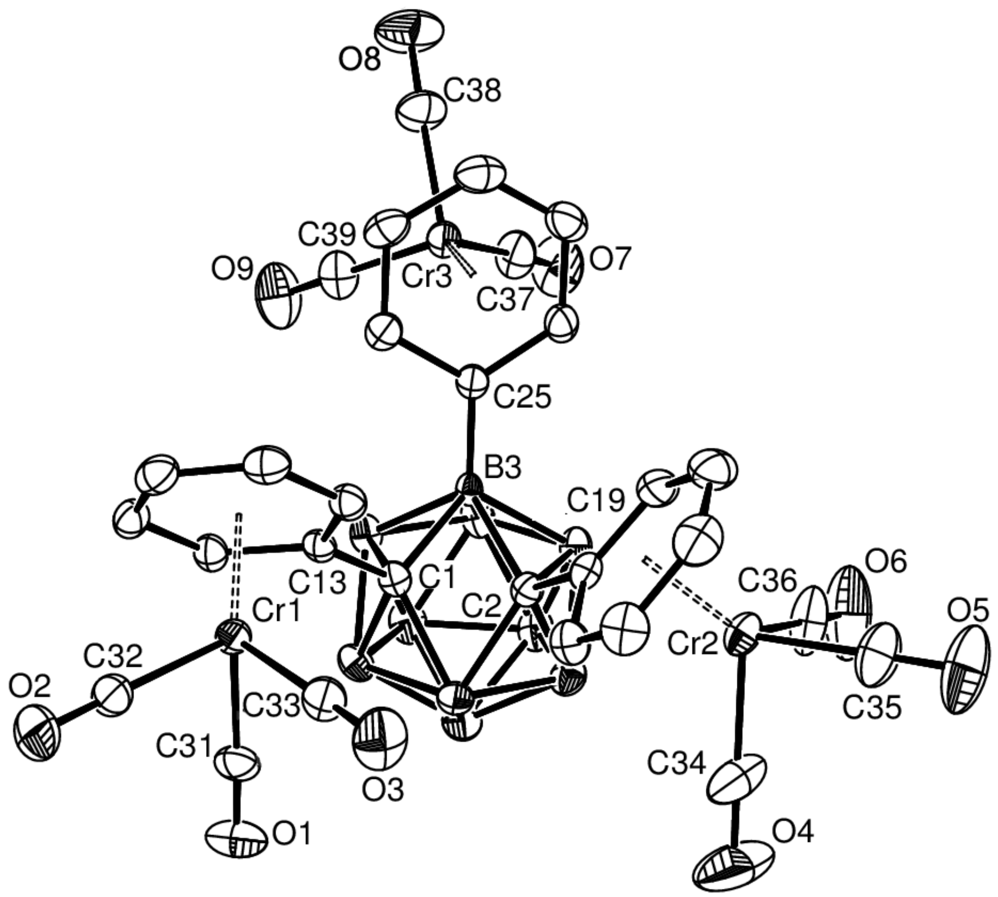

Figure S12. ORTEP drawing (30% probability for thermal ellipsoids) of **Ph3C2BCr3** (the hydrogen atoms are omitted for clarity).

Table S10. Crystal data and structure refinement of **1 – 3**, **Ph3C2B**, **Ph3C2BCr2**, and **Ph3C2BCr3**.

	**1**	**2**	**3**	**Ph3C2B**	**Ph3C2BCr2**	**Ph3C2BCr3**
Identification code	K120504	K130805	K131105	k110211	k120304	k140710
Empirical formula	$C_{14}H_{20}B_{10}$	$C_{17}H_{20}B_{10}Cr_1O_3$	$C_{20}H_{20}B_{10}Cr_2O_6$	$C_{20}H_{24}B_{10}$	$C_{26}H_{24}B_{10}Cr_2O_6$	$C_{30}H_{26}B_{10}Cr_3O_9Cl_2$
Formula weight	296.40	432.43	568.46	372.49	644.55	865.51
Temperature	293(2) K	293(2) K	293(2) K	293(2) K	293(2) K	293(2) K
Wavelength	0.71073 Å	0.71073 Å	0.71073 Å	0.71073 Å	0.71073 Å	0.71073 Å
Crystal system, space group	Monoclinic, $P2_1$/n	Monoclinic, $P2_1$/n	Triclinic, $P\bar{1}$	Monoclinic, $P2_12_12_1$	Monoclinic, $P2_1$/c	Orthorhombic, $P2_12_12_1$
Unit cell dimensions	a = 10.859(1) Å	a = 10.621(3) Å	17.540(2) Å, α = 105.746(2)º	a = 8.2745(7) Å	a = 16.531(3) Å	a = 12.783(2) Å
	b = 24.953(3) Å, β = 111.854(2)º	b = 17.056(5) Å, β = 106.622(5)º	18.060(2) Å, β = 110.226(2)º	b = 15.5626(13) Å	b = 11.276(2) Å	b = 14.909(2) Å
	c = 13.938(2) Å	c = 12.174(4) Å	19.484(3) Å, γ = 91.256(2)º	c = 16.2094(14) Å	c = 15.770(3) Å	c = 19.618(2) Å
Volume	3505.3(8)	2113.2(1)	5528.0(1)	2087.3(3) Å^3	2929.1(9) Å^3	3738.7(8) Å^3
Z, D_{calc}	8, 1.123	4, 1.359	2, 0.342	4, 1.185 g/cm^3	4, 1.462 g/cm^3	4, 1.538 g/cm^3
F(000)	1232.0	880.0	572	776	1304	1736
Crystal size	0.15, 0.13, 0.12	0.17, 0.15, 0.13	0.20, 0.20, 0.15	0.10, 0.08, 0.05	0.3, 0.2, 0.15	0.2, 0.2, 0.15
θ range for data collection	1.63 to 28.37	2.12 to 28.46	1.17 to 28.38	1.81 to 28.34,º	1.24 to 28.31	1.72 to 28.29
Limiting indices	$-14 \leq h \leq 14, -33 \leq k \leq 32, -18 \leq l \leq 18$	$-14 \leq h \leq 14, -22 \leq k \leq 22, -16 \leq l \leq 16$	$-23 \leq h \leq 23, -24 \leq k \leq 24, -26 \leq l \leq 25$	$-11 \leq h \leq 10, -20 \leq k \leq 20, -21 \leq l \leq 21$	$-22 \leq h \leq 22, -15 \leq k \leq 14, -20 \leq l \leq 21$	$-17 \leq h \leq 17, -19 \leq k \leq 19, -26 \leq l \leq 26$

Reflections collected/unique	35826/8726 [R(int) = 0.0421]	28490/5305 [R(int) = 0.0238]	44259/18014 [R(int) = 0.0398]	21438 / 5180 [R(int) = 0.0326]	29354 / 7259 [R(int) = 0.0281]	51105 / 9261 [R(int) = 0.0287]
Completeness to $\theta = 28.38$	99.3%	99.2%	65.0%	99.9 %	99.6 %	99.9 %
Refinement method	Full-matrix least-squares on F^2	Full-matrix least-squares on F^2	Full-matrix least-squares on F^2	Full-matrix least-squares on F^2	Full-matrix least-squares on F^2	Full-matrix least-squares on F^2
Data/restraints/parameters	8726/0/581	5305/0/360	18014/0/1409	5180 / 0 / 271	7259 / 0 / 406	9261 / 0 / 496
Goodness-of-fit on F^2	1.008	1.053	0.902	1.019	1.060	1.027
Final R indices [$I > 2\theta(I)$]	$^aR_1 = 0.0616$, $^bwR_2 = 0.1545$	$^aR_1 = 0.0332$, $^bwR_2 = 0.0958$	$^aR_1 = 0.0563$, $^bwR_2 = 0.1505$	$^aR_1 = 0.0427$, $^b\mathrm{w}R_2 = 0.1155$	$^aR_1 = 0.0422$, $^bwR_2 = 0.1228$	$^aR_1 = 0.0426$, $^bwR_2 = 0.1195$
R indices (all data)	$^aR_1 = 0.1037$, $^bwR_2 = 0.1897$	$^aR_1 = 0.0388$, $^bwR_2 = 0.1018$	$^aR_1 = 0.0877$, $^bwR_2 = 0.1612$	$^aR_1 = 0.0458$, $^b\mathrm{w}R_2 = 0.1190$	$^aR_1 = 0.0558$, $^bwR_2 = 0.1359$	$^aR_1 = 0.0479$, $^bwR_2 = 0.1255$
Largest diff. peak and hole	0.220 and –0.355 e.Å^{-3}	0.364 and –0.242 e.Å^{-3}	0.687 and –0.375 e.Å^{-3}	0.263 and −0.191 e.Å^{-3}	0.782 and −0.574 e.Å^{-3}	0.728 and −0.485 e.Å^{-3}

$^aR_1 = \sum||F_o|-|F_c||$ (based on reflections with $F_o^2>2\sigma F^2$), $^bwR_2 = [\sum[w(F_o^2-F_c^2)^2]/\sum[w(F_o^2)^2]]^{1/2}$; $w = 1/[\sigma^2(F_o^2)+(0.095P)^2]$; , $P = [\max(F_o^2, 0)+2F_c^2]/3$(also with $F_o^2>2\sigma F^2$)

Table S11. Comparison of selected bond lengths (Å), angles (º), and torsion angles (º) for **1 – 3**, **Ph3C2B**, **Ph3C2BCr2**, and **Ph3C2BCr3**.

	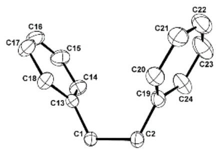 **1**	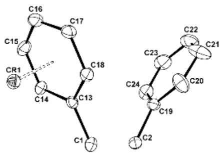 **2**	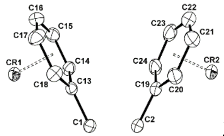 **3**	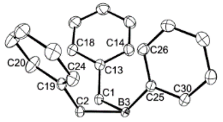 **Ph3C2B**	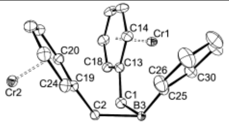 **Ph3C2BCr2**	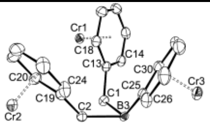 **Ph3C2BCr3**
Ph$_{C–C}$ (av)	1.376	1.400	1.406	1.387	1.382	1.407
Ph$_{C–C}$ (av)−Cr		2.210	2.212		1.410	1.407
Cab$_{C–C}$	1.726(2)	1.740(2)	1.724(4)	1.764(2)	1.776(3)	1.748(4)
Cent−Cr		1.702	1.696(av)		1.706(av)	1.705(av)
Cr–CO		1.856(av)	1.851(av)		1.849(av)	1.851(av)
C1–C13	1.507(2)	1.499(2)	1.502(4)	1.518(2)	1.581(3)	1.566(4)
C2–C19	1.501(2)	1.500(2)	1.510(4)	1.513(2)	1.511(3)	1.529(4)
B3–C25				1.568(2)	1.510(3)	1.506(4)
C13–C1–C2	118.3(1)	116.6(1)	116.4(2)	124.3(1)	122.2(2)	125.5(2)
C19–C2–C1	119.0(1)	119.6(1)	116.1(2)	122.3(9)	118.3(2)	120.3(2)
C25–B3–C1				124.2(1)	120.3(2)	119.3(2)
C25–B3–C2				121.3(1)	122.7(2)	120.6(2)
C1-C2-C19-C20	84.1(2)	86.1(2)	105.8(3)	62.3(2)	38.5(3)	65.3(3)
C1-B3-C25-C26				34.2(2)	130.4(2)	144.7(3)
B3-C1-C13-C18	–169.6(2)	–145.0(1)	4.3(4)	−143.4(1)	170.5(2)	−64.1(4)
C2-C1-C13-C14	81.7(2)	102.3(1)	112.4(3)	117.8(1)	60.9(3)	−173.4(2)
C2-B3-C25-C26				−39.2(2)	57.9(3)	75.5(3)
B3-C2-C19-C20	153.4(2)	155.5(1)	39.1(4)	133.6(1)	107.7(2)	136.1(3)
